# Future Trends in the Pharmacogenomics of Brain Disorders and Dementia: Influence of *APOE* and *CYP2D6* Variants

**DOI:** 10.3390/ph3103040

**Published:** 2010-09-29

**Authors:** Ramón Cacabelos, Lucía Fernández-Novoa, Rocío Martínez-Bouza, Adam McKay, Juan C. Carril, Valter Lombardi, Lola Corzo, Iván Carrera, Iván Tellado, Laura Nebril, Margarita Alcaraz, Susana Rodríguez, Ángela Casas, Verónica Couceiro, Antón Álvarez

**Affiliations:** 1 EuroEspes Biomedical Research Center, Institute for CNS Disorders and Genomic Medicine, Bergondo, 15165 Coruña, Spain; 2 EuroEspes Chair of Biotechnology and Genomics, Camilo José Cela University, Madrid, Spain

**Keywords:** Alzheimer’s disease, *APOE*, *CYPs*, genetics, pharmacogenomics

## Abstract

About 80% of functional genes in the human genome are expressed in the brain and over 1,200 different genes have been associated with the pathogenesis of CNS disorders and dementia. Pharmacogenetic studies of psychotropic drug response have focused on determining the relationship between variations in specific candidate genes and the positive and adverse effects of drug treatment. Approximately, 18% of neuroleptics are substrates of CYP1A2 enzymes, 40% of CYP2D6, and 23% of CYP3A4; 24% of antidepressants are substrates of CYP1A2 enzymes, 5% of CYP2B6, 38% of CYP2C19, 85% of CYP2D6, and 38% of CYP3A4; 7% of benzodiazepines are substrates of CYP2C19 enzymes, 20% of CYP2D6, and 95% of CYP3A4. 10-20% of Western populations are defective in genes of the CYP superfamily; and the pharmacogenomic response of psychotropic drugs also depends on genetic variants associated with dementia. Prospective studies with anti-dementia drugs or with multifactorial strategies have revealed that the therapeutic response to conventional drugs in Alzheimer’s disease is genotype-specific. The disease-modifying effects (cognitive performance, biomarker modification) of therapeutic intervention are APOE-dependent, with *APOE-4* carriers acting as the worst responders (*APOE-3/3* > * APOE-3/4* > * APOE-4/4*). *APOE-CYP2D6* interactions also influence the therapeutic outcome in patients with dementia.

## 1. Introduction

From an epidemiological perspective, central nervous system (CNS) disorders are the third largest health problem in developed countries, representing 10–15% of deaths, after cardiovascular disorders (25–30%) and cancer (20–25%). Approximately 127 million Europeans suffer brain disorders. The total annual cost of brain disorders in Europe is about €386 billion, with €135 billion of direct medical expenditures (€78 billion, inpatients; €45 billion, outpatients; €13 billion, pharmacological treatment), €179 billion of indirect costs (lost workdays, productivity loss, permanent disability), and €72 billion of direct non-medical costs. Mental disorders represent €240 billion (62% of the total cost, excluding dementia), followed by neurological diseases (€84 billion, 22%) [1]. In low- and middle-income countries, dementia makes the largest contribution to disability with a median population-attributable prevalence fraction (PAPF) of 25.1%, followed by stroke (11.4%), limb impairment (10.5%), arthritis (9.9%), depression (8.3%), eyesight problems (6.8%), and gastrointestinal impairments (6.5%) [2]. Alzheimer’s disease (AD) is the most frequent form of dementia (50–70%), followed by vascular dementia (30–40%), and mixed dementia (15–20%). These prevalent forms of age-related neurodegeneration affect over 25 million people at present, and probably over 75 million people will be at risk in the next 20–25 years worldwide. The prevalence of dementia increases exponentially from approximately 1% at 60–65 years of age to over 30–35% in people of over 80 years. It is likely that in patients older than 75–80 years of age most cases of dementia are mixed in nature (degenerative + vascular), whereas pure AD cases are very rare after 80 years of age. The average annual cost per person with dementia ranges from €10,000 to €40,000, depending upon disease stage and country, with a lifetime cost per patient of more than €150,000. In some countries, approximately 80% of the global costs of dementia (direct + indirect costs) are assumed by the patients and/or their families. About 10–20% of the costs in dementia are attributed to pharmacological treatment, including anti-dementia drugs, psychotropics (antidepressants, neuroleptics, anxiolytics), and other drugs currently prescribed in the elderly (antiparkinsonians, anticonvulsants, vasoactive compounds, anti-inflammatory drugs, *etc.*). In addition, during the past 20 years over 300 drugs have been partially or totally developed for AD, with the subsequent costs for the pharmaceutical industry, and only five drugs with moderate-to-poor efficacy and questionable cost-effectiveness have been approved in developed countries [3,4,5].

### 1.1. Towards a Personalized Medicine in Neuropsychiatric Disorders

Common features in CNS disorders include the following: (i) polygenic/complex disorders in which genomic and environmental factors are involved; (ii) deterioration of higher activities of the CNS; (iii) multifactorial dysfunctions in several brain circuits; and (iv) accumulation of toxic proteins in the nervous tissue in cases of neurodegeneration. For instance, the neuropathological hallmark of AD (amyloid deposition in senile plaques, neurofibrillary tangle formation, and neuronal loss) is but the phenotypic expression of a pathogenic process in which different gene clusters and their products are potentially involved [6,7]. 

Extensive molecular genetics studies carried out in the past two decades have demonstrated that most CNS disorders are multifactorial, polygenic/complex disorders in which hundreds of genes distributed across the human genome might be involved. For example, 255 genes have been associated with dementia (Table 1), 205 with schizophrenia, 106 with depression, 107 with anxiety, 103 with stroke, 385 with different types of ataxia, 155 with epilepsia, 83 with meningioma, 105 with glioblastoma, 27 with astrocytoma, 73 with Parkinson’s disease, and over 30 genes with cerebrovascular disorders. Many of these genetic associations could not be replicated in different settings and different populations due to many complex (methodological, technological) factors [8,9,10]. Furthermore, the same genomic defect can give rise to apparently diverse phenotypes, and different genomic defects can converge in an apparently common phenotype, this increasing the complexity of genomic studies (e.g., patient recruitment, pure controls, concomitant pathology, epigenetic factors, environmental factors) [10]. 

**Table 1:** Selected human genes investigated as potential candidate genes associated with dementia and age-related neurodegenerative disorders [4,5,6,9,20,21,22].

Locus	Symbol	Aliases	Title
1p21.3-p13.1	*SORT1*	*Gp95, NT3*	sortilin
1p31.3	*TM2D1*	*BBP*	TM2 domain containing 1
1p32	*ERI3*	*PINT1; PRNPIP; MGC2683; FLJ22943*	ERI1 exoribonuclease family member 3
1p32.3	*ZFYVE9*	*MADHIP, NSP, SARA, SMADIP*	zinc finger, FYVE domain containing 9
1p33-p31.1	*DHCR24*	*KIAA0018, Nbla03646, SELADIN1, seladin-1*	24-dehydrocholesterol reductase
1p34	*LRP8*	*APOER2, HSZ75190, MCI1*	low density lipoprotein receptor-related protein 8, apolipoprotein e receptor
1p36.1	*ECE1*	*RP3-329E20.1, ECE*	endothelin converting enzyme 1
1p36.13-q31.3	*APH1A*	*RP4-790G17.3, 6530402N02Rik, APH-1, APH-1A, CGI-78*	anterior pharynx defective 1 homolog A (C. elegans)
1p36.22	*TARDBP*	*RP4-635E18.2, ALS10, TDP-43*	TAR DNA binding protein
1p36.3	*MTHFR*		5,10-methylenetetrahydrofolate reductase (NADPH)
1q21	*S100A1*	*S100, S100-alpha, S100A*	S100 calcium-binding protein A1
1q21.2-q21.3	*LMNA*	*RP11-54H19.1, CDCD1, CDDC, CMD1A, CMT2B1, EMD2, FPL, FPLD, HGPS, IDC, LDP1, LFP, LGMD1B, LMN1, LMNC, PRO1*	lamin A/C
1q21.3	*CHRNB2*	*EFNL3, nAChRB2*	cholinergic receptor, nicotinic, beta 2 (neuronal)
1q21-q23	*APCS*	*MGC88159, PTX2, SAP*	amyloid P component, serum
1q22-q23	*NCSTN*	*RP11-517F10.1, APH2, KIAA0253*	nicastrin
1q25	*SOAT1*	*RP11-215I23.1, ACACT, ACAT, ACAT1, RP11-215I23.2, SOAT, STAT*	sterol O-acyltransferase 1
1q25.2-q25.3	*PTGS2*	*COX-2, COX2, GRIPGHS, PGG/HS, PGHS-2, PHS-2, hCox-2*	prostaglandin-endoperoxide synthase 2 (prostaglandin G/H synthase and cyclooxygenase)
1q31-q32	*IL10*	*CSIF, IL-10, IL10A, MGC126450, MGC126451, TGIF*	interleukin 10
1q31-q42	*AD4*	*AD3L, AD4, PS2, STM2*	presenilin 2 (alzheimer disease 4)
1q32	*CR1*	*C3BR, C4BR, CD35, KN*	complement component (3b/4b) receptor 1 (Knops blood group)
1q42-q43	*AGT*	*ANHU, FLJ92595, FLJ97926, SERPINA8*	angiotensinogen (serpin peptidase inhibitor, clade A, member 8)
2p16.3	*RTN4*	*ASY, NI220/250, NOGO, NOGO-A, NOGOC, NSP, NSP-CL, Nbla00271, Nbla10545, Nogo-B, Nogo-C, RTN-X, RTN4-A, RTN4-B1, RTN4-B2, RTN4-C*	reticulon 4
2p25	*ADAM17*	*ADAM18, CD156B, CSVP, MGC71942, TACE*	ADAM metallopeptidase domain 17
2q14	*BIN1*	*AMPH2, AMPHL, DKFZp547F068, MGC10367, SH3P9*	bridging integrator 1
2q14	*IL1A*	*IL-1A, IL1, IL1-ALPHA, IL1F1*	interleukin-1-Alpha
2q21.1	*KCNIP3*	*CSEN, DREAM, KCHIP3, MGC18289*	Kv channel interacting protein 3, calsenilin
2q21.2	*LRP1B*	*LRP-DIT, LRPDIT*	low density lipoprotein-related protein 1B (deleted in tumors)
2q34	*CREB1*	*CREB, MGC9284*	cAMP responsive element binding protein 1
3q25.1-q25.2		*CALLA, CD10, MME DKFZp686O16152, MGC126681, MGC126707, NEP*	membrane metallo-endopeptidase
3q26.1-q26.2	*BCHE*	*CHE1, E1*	butyrylcholinesterase
3q26.2-qter		*APOD*	apolipoprotein D
3q28	*SST*	*SMST*	somatostatin
4p14-p13	*APBB2*	*DKFZp434E033, FE65L, FE65L1, MGC35575*	amyloid beta (A4) precursor protein-binding, family B, member 2
5q15	*CAST*	*BS-17, MGC9402*	calpastatin
5q31	*APBB3*	*FE65L2, MGC150555, MGC87674, SRA*	amyloid beta (A4) precursor protein-binding, family B, member 3
5q35.3	*DBN1*	*D0S117E, DKFZp434D064*	drebrin 1
6p12	*VEGFA*	*RP1-261G23.1, MGC70609, MVCD1, VEGF, VPF*	vascular endothelial growth factor A
6p21.3	*AGER*	*DAMA-358M23.4, MGC22357, RAGE*	advanced glycosylation end product-specific receptor
6p21.3	*HFE*	*HFE1, HH, HLA-H, MGC103790, MVCD7, dJ221C16.10.1*	hemochromatosis
6p21.3	*HLA-A*	*DAQB-90C11.16, Aw-68, Aw-69, FLJ26655, HLAA*	major histocompatibility complex, class I, A
6p21.3	*TNF*	*DADB-70P7.1, DIF, TNF-alpha, TNFA, TNFSF2*	tumor necrosis factor (TNF superfamily, member 2)
6p22.1	*PGDB1*	*HUCEP-4, SCAND4, dJ874C20.4*	piggyBac transposable element derived 1
6p23	*ATXN1*	*ATX1, D6S504E, SCA1*	ataxin 1
7p21	*IL6*	*BSF2, HGF, HSF, IFNB2, IL-6*	interleukin 6 (interferon, beta 2)
7q21.3	*PON1*	*ESA, MVCD5, PON*	paraoxonase 1
7q22	*RELN*	*PRO1598, RL*	reelin
7q36	*AD10*		Alzheimer disease-10
7q36	*NOS3*	*ECNOS, eNOS*	nitric oxide synthase 3 (endothelial cell)
7q36	*PAXIP1*	*CAGF28, CAGF29, FLJ41049, PACIP1, PAXIP1L, PTIP, TNRC2*	PAX interacting (with transcription-activation domain) protein 1
8p21-p12	*CLU*	*AAG4, APOJ, CLI, KUB1, MGC24903, SGP-2, SGP2, SP-40, TRPM-2, TRPM2*	clusterin
8p22	*CTSB*	*APPS, CPSB*	cathepsin B
9p24.1	*IL33*	*C9orf26, DKFZp586H0523, DVS27, NF-HEV, NFEHEV, RP11-575C20.2*	interleukin 33
9q13-q21.1	*APBA1*	*D9S411E, MINT1, X11, X11A, X11ALPHA*	amyloid beta (A4) precursor protein-binding, family A, member 1
9q31.1	*GRIN3A*	*FLJ45414, NMDAR-L, NR3A*	glutamate receptor, ionotropic, N-methyl-D-aspartate 3A
9q33-q34.1	*HSPA5*	*BIP, FLJ26106, GRP78, MIF2*	heat shock 70kDa protein 5 (glucose-regulated protein, 78kDa)
9q34.1	*DAPK1*	*DAPK, DKFZp781I035*	death-associated protein kinase 1
10p13	*AD7*		Alzheimer disease 7
10p15.2	*PITRM1*	*RP11-298E9.1, KIAA1104, MGC138192, MGC141929, MP1, PreP, hMP1*	pitrilysin metallopeptidase 1
10q	*AD6*		Alzheimer disease-6
10q11.2	*ALOX5*	*RP11-67C2.3, 5-LO, 5-LOX, 5LPG, LOG5, MGC163204*	arachidonate 5-lipoxygenase
10q21	*TFAM*	*MtTF1, TCF6, TCF6L1, TCF6L2, TCF6L3, mtTFA*	transcription factor A, mitochondrial
10q23	*CH25H*	*C25H*	cholesterol 25-hydroxylase
10q23-q25	*IDE*	*RP11-366I13.1, FLJ35968, INSULYSIN*	insulin-degrading enzyme
10q23-q25	*SORCS1*	*RP11-446H13.1, FLJ41758, FLJ43475, FLJ44957*	sortilin-related VPS10 domain containing receptor 1
10q23.32	*HECTD2*	*FLJ16050*	HECT domain containing 2
10q24	*COX15*		COX15 homolog, cytochrome c oxidase assembly protein (yeast)
10q24	*PLAU*	*ATF, UPA, URK, u-PA*	plasminogen activator, urokinase
10q24.33	*CALHM1*	*FAM26C, MGC39514, MGC39617*	calcium homeostasis modulator 1
10q24.33	*SH3PXD2A*	*FISH, SH3MD1*	SH3 and PX domains 2A
10q26.3	*ADAM12*	*RP11-295J3.5, MCMP, MCMPMltna, MLTN, MLTNA*	ADAM metallopeptidase domain 12
11p13	*BDNF*	*MGC34632*	brain-derived neurotrophic factor
11p15	*APBB1*	*FE65, MGC:9072, RIR*	amyloid beta (A4) precursor protein-binding, family B, member 1 (Fe65)
11p15.1	*SAA1*	*MGC111216, PIG4, SAA, TP53I4*	serum amyloid A1
11p15.5	*CTSD*	*CLN10, CPSD, MGC2311*	cathepsin D
11q14	*PICALM*	*CALM, CLTH, LAP*	phosphatidylinositol binding clathrin assembly protein
11q14.1	*GAB2*	*KIAA0571*	GRB2-associated binding protein 2
11q23.2-q23.3	*BACE1*	*ASP2, BACE, FLJ90568, HSPC104, KIAA1149*	beta-site APP-cleaving enzyme 1
11q23.2-q24.2	*SORL1*	*C11orf32, FLJ21930, FLJ39258, LR11, LRP9, SORLA, SorLA-1, gp250*	sortilin-related receptor, L(DLR class) A repeats-containing
11q24	*APLP2*	*APPH, APPL2, CDEBP*	amyloid beta (A4) precursor-like protein 2
12p11.23-q13.12	*AD5*		Alzheimer disease 5
12p12.3-p12.1	*IAPP*	*AMYLIN, DAP, IAP*	islet amyloid polypeptide
12p13.3-p12.3	*A2M*	*CPAMD5, DKFZp779B086, FWP007, S863-7*	alpha-2-macroglobulin
12q13-q14	*LRP1*	*A2MR, APOER, APR, CD91, FLJ16451, IGFBP3R, LRP, MGC88725, TGFBR5*	low density lipoprotein-related protein 1 (alpha-2-macroglobulin receptor)
13q34	*DAOA*	*G72, LG72, SG72*	D-amino acid oxidase activator
14q24.3	*FOS*	*AP-1, C-FOS*	FBJ murine osteosarcoma viral oncogene homolog
14q24.3	*PSEN1*	*AD3, FAD, PS1, S182*	presenilin-1
14q32	*RAGE*	*MOK, RAGE1*	renal tumor antigen
14q32.1	*CYP46A1*	*CP46, CYP46*	cytochrome P450, family 46, subfamily A, polypeptide 1
14q32.1	*SERPINA3*	*AACT, ACT, GIG24, GIG25, MGC88254*	serpin peptidase inhibitor, clade A (alpha-1 antiproteinase, antitrypsin), member 3
15q21.1	*CYP19A1*	*ARO, ARO1, CPV1, CYAR, CYP19, MGC104309, P-450AROM*	cytochrome P450, family 19, subfamily A, polypeptide 1
15q22.2	*APH1B*	*APH-1B, DKFZp564D0372, FLJ33115, PRO1328, PSFL, TAAV688*	anterior pharynx defective 1 homolog B (C. elegans)
15q11-q12	*APBA2*	*D15S1518E, HsT16821, LIN-10, MGC99508, MGC:14091, MINT2, X11L*	amyloid beta (A4) precursor protein-binding, family A, member 2
16p13.3	*UBE2I*	*C358B7.1, P18, UBC9*	ubiquitin-conjugating enzyme E2I (UBC9 homolog, yeast)
16q21	*CETP*	*HDLCQ10*	cholesteryl ester transfer protein, plasma
16q22	*NAE1*	*A-116A10.1, APPBP1, HPP1, ula-1*	NEDD8 activating enzyme E1 subunit 1
17p12-p11.2	*COX10*		COX10 homolog, cytochrome c oxidase assembly protein, heme A: farnesyltransferase (yeast)
17p13	*MYH13*	*MyHC-eo*	myosin, heavy chain 13, skeletal muscle
17p13.1	*TNK1*	*MGC46193*	tyrosine kinase, non-receptor, 1
17q11.2	*BLMH*	*BH, BMH*	bleomycin hydrolase
17q11.2	*MIR144*	*MIRN144*	microRNA 144
17q21.1	*MAPT*	*DDPAC, FLJ31424, FTDP-17, MAPTL, MGC138549, MSTD, MTBT1, MTBT2, PPND, TAU*	microtubule-associated protein tau
17q21.1	*STH*	*MAPTIT, MGC163191, MGC163193*	saitohin
17q21.32	*GRN*	*GEP, GP88, PCDGF, PEPI, PGRN*	granulin
17q21-q22	*GPSC*		gliosis, familial progressive subcortical
17q21-q23	*APPBP2*	*HS.84084, KIAA0228, PAT1*	amyloid beta precursor protein (cytoplasmic tail) binding protein 2
17q23.1	*MPO*		myeloperoxidase
17q23.3	*ACE*	*ACE1, CD143, DCP, DCP1, MGC26566, MVCD3*	angiotensin I converting enzyme (peptidyl-dipeptidase A) 1
17q24.3	*BPTF*	*FAC1, FALZ, NURF301*	bromodomain PHD finger transcription factor
18q12.1	*TTR*	*HsT2651, PALB, TBPA*	transthyretin
19p13	*PIN1*	*DOD, UBL5*	peptidylprolyl cis/trans isomerase, NIMA-interacting 1
19p13.2	*AD9*		Alzheimer disease 9
19p13.2-p13.1	*NOTCH3*	*CADASIL, CASIL*	Notch homolog 3 (Drosophila)
19p13.3	*APBA3*	*MGC:15815, X11L2, mint3*	amyloid beta (A4) precursor protein-binding, family A, member 3
19p13.3	*GRIN3B*	*NR3B*	glutamate receptor, ionotropic, N-methyl-D-aspartate 3B
19p13.3-p13.2	*ICAM*	*BB2, CD54, P3.58*	intercellular adhesion molecule 1
19q13	*TOMM40*	*C19orf1, D19S1177E, PER-EC1, PEREC1, TOM40*	translocase of outer mitochondrial membrane 40 homolog (yeast)
19q13.1	*APLP1*	*APLP*	amyloid beta (A4) precursor-like protein 1
19q13.12	*PEN2*	*MDS033, MSTP064, PEN-2, PEN2*	presenilin enhancer 2 homolog (C. elegans)
19q13.2	*APOE*	*AD2, LDLCQ5, LPG, MGC1571*	apolipoprotein E
19q13.2	*APOC1*		apolipoprotein C-I
19q13.32	*BLOC1S3*	*BLOS3, FLJ26641, FLJ26676, HPS8, RP*	biogenesis of lysosomal organelles complex-1, subunit 3
19q13.32	*EXOC3L2*	*FLJ36147, MGC16332, XTP7*	exocyst complex component 3-like 2
19q13.3	*MARK4*	*FLJ90097, KIAA1860, MARKL1, Nbla00650*	MAP/microtubule affinity-regulating kinase 4
19q13.43	*GALP*		galanin-like peptide
20p	*AD8*		Alzheimer disease-8
20p11.21	*CST3*	*ARMD11, MGC117328*	cystatin C
20p13	*PRNP*	*ASCR, CD230, CJD, GSS, MGC26679, PRIP, PrP, PrP27-30, PrP33-35C, PrPc, prion*	prion protein
20q13.31	*PCK1*	*MGC22652, PEPCK-C, PEPCK1, PEPCKC*	phosphoenolpyruvate carboxykinase 1 (soluble)
21q21.3	*APP*	*AAA, ABETA, ABPP, AD1, APPI, CTFgamma, CVAP, PN2*	amyloid beta (A4) precursor protein
21q22.3	*BACE2*	*AEPLC, ALP56, ASP1, ASP21, BAE2, CDA13, CEAP1, DRAP*	beta-site APP-cleaving enzyme 2
22q11.21	*RTN4R*	*NGR, NOGOR*	reticulon 4 receptor
	*HN*		humanin
22q11.21	*COMT*		catechol-O-methyltransferase

Drug metabolism, and the mechanisms underlying drug efficacy and safety, are also genetically-regulated complex traits in which hundreds of genes cooperatively participate. Disease-associated genomics, transcriptomics, proteomics and metabolomics are essential components of the therapeutic outcome [11]. Pharmacogenetic and pharmacogenomic factors may account for 60–90% of drug variability in drug disposition and pharmacodynamics. About 10–20% of Caucasians are carriers of defective *CYP2D6* polymorphic variants which alter the metabolism of many psychotropic agents. The incorporation of pharmacogenetic/pharmacogenomic protocols into CNS research and clinical practice can foster the optimization of therapeutics by helping to develop cost-effective pharmaceuticals and improving drug efficacy and safety [6,7,8,11,12,13,14,15,16,17,18,19,20,21].

## 2. Genomics of Dementia

Approximately 5% of the human genome is structurally variant in the normal population [18]. There are roughly 7–10 million positions in the human genome that can show variability among individuals, and differences in the DNA sequence are the genetic basis of human variability and complex traits. The spectrum of variation in the human genome includes: (i) single changes (single nucleotide polymorphisms (SNPs), point mutations) (1 bp); (ii) small insertions/deletions (binary insertion/deletion events of short sequences) (1–50 bp); (iii) short tandem repeats (microsatellites) (1–500 bp); (iv) fine-scale structural variation (deletions, duplications, tandem repeats, inversions) (50 bp –5 kb); (v) retroelement insertions (SINEs, LINEs, LTRs, ERVs) (300 bp–10 kb); (vi) intermediate-scale structural variations (deletions, duplications, tandem repeats, inversions) (5 kb–0 kb); (vii) large-scale structural variation (deletions, duplications, large tandem repeats) (50 kb–5 Mb); and (viii) chromosomal variations (euchromatic variations, cytogenetic deletions, duplications, translocations, inversions, and aneuplidy) (>5 Mb) [18,19]. Segmental duplications of low copy repeats are blocks of DNA ranging from 1–400 kb in length which occur at multiple sites within the genome and typically share a high level (>95%) of sequence identity [18]. Segmental duplications frequently mediate polymorphic rearrangements of intervening sequences via non-allelic homologous recombination (NAHR) with major implications for human disease. SNPs and insertion (I)/deletion (D) events are the most frequent types of structural variation. I/D polymorphisms of several genes with functions in enzymatic pathways or in drug metabolizing enzymes (e.g., *CYP2D6*) may drastically influence a variety of common phenotypes with pathogenic and/or pharmacogenetic relevance. The differential expression of common variants is a major source of genetic variation with important repercussions in human diversity and disease heterogeneity. Prior to the completion of the Human Genome Project and the emergence of dense genetic maps, scientists used linkage studies and positional cloning to identify DNA mutations in rare diseases, but in the past two decades association study designs became more powerful compared with linkage study designs in identifying susceptibility loci and SNP variation. Currently, over 10 million DNA sequence variations have been uncovered in the human genome [19]. 

### 2.1. Structural Genomics of Alzheimer’s Disease

The genetic defects identified in AD can be classified into three main categories: (a) Mendelian mutations in AD primary genes; (b) multiple susceptibility SNPs in many different genes distributed across the human genome; and (c) mitochondrial DNA (mtDNA) mutations. 

(a) Mendelian or mutational defects in genes directly linked to AD, including (i) >30 mutations in the amyloid beta (Aβ) precursor protein (*APP*) gene (21q21) (AD1); (ii) >160 mutations in the presenilin 1 (*PSEN1*) gene (14q24.3) (AD3); and (iii) >10 mutations in the presenilin 2 (*PSEN2*) gene (1q31–q42) (AD4) [8,20,21]. *PSEN1* and *PSEN2* are important determinants of γ-secretase activity responsible for proteolytic cleavage of APP and NOTCH receptor proteins. Mendelian mutations are very rare in AD (1:1000). Mutations in exons 16 and 17 of the *APP* gene appear with a frequency of 0.30% and 0.78%, respectively, in AD patients. Likewise, *PSEN1*, *PSEN2*, and microtubule-associated protein Tau (*MAPT*) (17q21.1) mutations are present in less than 2% of the cases. Mutations in these genes confer specific phenotypic profiles to patients with dementia: amyloidogenic pathology associated with *APP*, *PSEN1* and *PSEN2* mutations; and tauopathy associated with MATP mutations, representing the two major pathogenic hypotheses for AD [8,23,24,25]. 

(b) Multiple polymorphic risk variants characterized in over 200 different genes can increase neuronal vulnerability to premature death (Table 1) [8]. Among susceptibility genes, the apolipoprotein E (*APOE*) gene (19q13.2)(AD2) is the most prevalent as a risk factor for AD, especially in those subjects harboring the *APOE-4* allele, whereas carriers of the *APOE-2* allele might be protected against dementia [8]. *APOE*-related pathogenic mechanisms are also associated with brain aging and with the neuropathological hallmarks of AD. 

In 1993 Allen Roses and co-workers found a clear association between *APOE* genotypes and AD, demonstrating that the frequency of the *APOE-4* allele was significantly higher in LOAD [26,27,28]. Since then, many other studies have confirmed the early findings of Saunders *et al.* [27,28] and Corder *et al.* [29] reporting an increased frequency of the *APOE-4* allele in AD and the association of the *APOE-4* allele with LOAD and sporadic forms of AD [26,27,28,29,30,31]. *APOE-4* may influence AD pathology interacting with APP metabolism and Aβ accumulation, enhancing hyperphosphorylation of tau protein and NFT formation, reducing choline acetyltransferase activity, increasing oxidative processes, modifying inflammation-related neuroimmunotrophic activity and glial activation, altering lipid metabolism, lipid transport and membrane biosynthesis in sprouting and synaptic remodeling, and inducing neuronal apoptosis [8,17,26,27,28,29,30,31,32,33]. Age-related changes in brain structure and function have been reported as potential indicators of premature neurodegeneration [34].

Other genes of this category are included in Table 1. One of the newest members of the AD-gene family is *SORL1*, a gene which encodes a mosaic protein with a domain structure which suggests it is a member of both the vacuolar protein sorting-10 (Vps10) domain-containing receptor family and the low density lipoprotein receptor (LDLR). Inherited variants of the *SORL1* neuronal sorting receptor are associated with late-onset AD. Polymorphisms in two different clusters of intronic sequences within the *SORL1* gene may regulate tissue-specific expression of SORL1, which directs trafficking of APP into recycling pathways. When SORL1 is underexpressed, APP is sorted into Aß-generating compartments leading to amyloid accumulation in neuronal tissues [35]. As with many other potential AD-related genes, the association of *SORL1* with AD [35,36] could not be replicated in other studies [37]. 

Sorting protein-related receptor with A-type repeats (*SORLA*) is a major risk factor in cellular processes leading to AD. It acts as a sorting receptor for the APP that regulates intracellular trafficking and processing into amyloidogenic-beta peptides (Aβ). Overexpression of *SORLA* in neurons reduces while inactivation of gene expression accelerates amyloidogenic processing and senile plaque formation. Brain-derived neurotrophic factor (BDNF) is a major inducer of *SORLA* that activates receptor gene transcription through the ERK (extracellular regulated kinase) pathway. Expression of the receptor is significantly impaired in mouse models with genetic (*Bdnf*(-/-)) or disease-related loss of BDNF activity in the brain (Huntington’s disease). Exogenous application of BDNF reduced Aβ production in primary neurons and in the brain of wild-type mice *in vivo*, but not in animals genetically deficient for *Sorla*. According to these findings reported by Rohe *et al.* [38], the beneficial effects ascribed to BDNF in APP metabolism act through induction of *Sorla* which encodes a negative regulator of neuronal APP processing. The presence of the *BDNF* Val allele in itself and in combination with the *APOE-4* allele can be risk factors for AD, Lewy body dementia and Pick’s disease [39]. 

Another interesting gene is *DHCR24* (3β-hydroxysterol-δ-24-reductase) or *Seladin-1*, a key element in the cholesterologenic pathway in which the DHCR24 enzyme catalyzes the transformation of desmosterol into cholesterol [40,41]. *Seladin-1* was originally identified as a gene whose expression was down-regulated in the AD brain, demonstrating a neuroprotective effect against neurodegeneration. Recent studies indicate that *Seladin-1*/*DHCR24* is an LXR (liver X nuclear hormone receptor) target gene potentially involved in the regulation of lipid raft formation [40]. Polymorphisms in the cholesteryl ester transfer protein (*CETP*) gene have been associated with exceptional longevity and lower cardiovascular risk, but associations with memory decline and dementia risk are unclear. Sanders *et al.* [42] tested the hypothesis that a SNP at *CETP* codon 405 (isoleucine to valine V405; SNP rs5882) is associated with a lower rate of memory decline and lower risk of incident dementia, including AD. Compared with isoleucine homozygotes, valine homozygotes had significantly slower memory decline and lower risk of dementia. 

Another gene, with potential therapeutic interest as a tau kinase, might be the *GSK3* gene. Analysis of the promoter and all 12 exons revealed that an intronic polymorphism (IVS2-68G > A) occurred at over twice the frequency among patients with frontotemporal dementia (10.8%) and patients with AD (14.6%) than in aged healthy subjects (4.1%). This is the first evidence that a gene known to be involved in tau phosphorylation is associated with risk for primary neurodegenerative dementias [43]. Promoter polymorphisms modulating HSPA5 expression might also increase susceptibility to AD. Endoplasmic reticulum chaperone heat shock 70 kDa protein 5 (HSPA5/GRP78) is known to be involved in APP metabolism and neuronal death in AD. Of the three major polymorphisms (-415G/A (rs391957), -370C/T (rs17840761), -180del/G (rs3216733)), the *HSPA5*-415G/A and -180del/G variants showed significant differences between AD cases and controls. Subjects harboring the -415AA/-180GG genotype or the -415A/-180G allele might be less susceptible to develop AD [44]. The rs5952C and rs1568566T alleles of the *APOD* rs5952T/C and rs1568566C/T variants increase the risk for AD, whereas the rs5952T-rs1568566C haplotype reduces it [45]. 

ApoD is a lipoprotein-associated glycoprotein which is increased in the hippocampus and CSF of AD patients [45]. *CALHM1* encodes a multipass transmembrane glycoprotein that controls cytosolic Ca^2+^ concentrations and Aβ levels. The *CALHM1* P86L polymorphism (rs2986017) has been associated with AD [46]. 

Harold *et al.* [47] undertook a two-stage genome-wide association study (GWAS) of AD involving over 16,000 individuals, and found association with SNPs at two loci not previously associated with the disease, at the *CLU* (Clusterine, *APOJ*) gene (rs11136000) and 5' to the *PICALM* gene (rs3851179). In another GWAS with patients from France, Belgium, Finland, Italy and Spain, Lambert *et al.* [48] found association with *CLU* and with the *CR1* gene, encoding the complement component (3b/4b) receptor 1, on chromosome 1 (rs6656401). 

Jun *et al.* [49] determined whether genotypes at *CLU*, *PICALM*, and *CR1* confer risk for Alzheimer disease (AD) and whether risk for AD associated with these genes is influenced by APOE genotypes in 7070 cases with AD, 3055 with autopsies; and 8169 elderly cognitively normal controls, 1092 with autopsies, from 12 different studies, including white, African American, Israeli-Arab, and Caribbean Hispanic individuals. They confirmed in a completely independent data set that *CR1* (rs3818361), *CLU* (rs11136000), and *PICALM* (rs3851179) are AD susceptibility loci in European ancestry populations. Genotypes at *PICALM* confer risk predominantly in *APOE-4*-positive subjects. Thus, *APOE* and *PICALM* synergistically interact. Two new loci were identified to have genome-wide significance for the first time: rs744373 near *BIN1* and rs597668 near *EXOC3L2/BLOC1S3/MARK4* [50]. 

Kramer *et al.* [51] conducted a GWAS to identify genetic mechanisms that distinguish non-demented elderly with a heavy NFT burden from those with a low NFT burden. Both a genotype test, using logistic regression, and an allele test provided consistent evidence that variants in the *RELN* gene are associated with neuropathology in the context of cognitive health. Immunohistochemical data for reelin expression in AD-related brain regions added support for these findings. Reelin signaling pathways modulate phosphorylation of tau, the major component of NFTs, either directly or through beta-amyloid pathways that influence tau phosphorylation. Up-regulation of reelin may be a compensatory response to tau-related or beta-amyloid stress associated with AD even prior to the onset of dementia [51]. Aβ induces synaptic dysfunction in part by altering the endocytosis and trafficking of AMPA and NMDA receptors. Reelin is a neuromodulator that increases glutamatergic neurotransmission by signaling through the postsynaptic ApoE receptors ApoER2 and VLDLR and thereby potently enhances synaptic plasticity. Reelin can prevent the suppression of long-term potentiation and NMDA receptors, which is induced by levels of Aβ comparable to those present in an AD-afflicted brain. This reversal is dependent upon the activation of Src family tyrosine kinases. Durakoglugil *et al.* [52] have proposed that Aβ, Reelin, and ApoE receptors modulate neurotransmission and thus synaptic stability as opposing regulators of synaptic gain control.

A variable-length, deoxythymidine homopolymer (poly-T) within intron 6 of the *TOMM40* gene was found to be associated with the age of onset of LOAD by Roses *et al.* [53]. This result was obtained with a phylogenetic study of the genetic polymorphisms that reside within the linkage disequilibrium (LD) block that contains the *TOMM40*, *APOE*, and *APOC1* genes from patients with LOAD and age-matched subjects without disease [54]. These new data explain the mean age at disease onset for patients with the APOE4/4 genotype and differentiate two forms of TOMM40 poly-T polymorphisms linked to APOE, with each form associated with a different age at disease onset distribution. When linked to APOE3 (encoding the epsilon3 isoform of APOE), the longer TOMM40 poly-T repeats (19–39 nucleotides) at the rs10524523 (hereafter, 523) locus are associated with earlier age at onset and the shorter TOMM40 523 alleles (11–16 nucleotides) are associated with later age at onset. According to Roses (2010) [55], the data suggest that the poly-T alleles are codominant, with the age at onset phenotype determined by the two inherited 523 alleles, but with variable expressivity. 

Ohe and Maeda [56] reported that overexpression of high-mobility group A protein 1a (HMGA1a) causes aberrant exon 5 skipping of the presenilin-2 (*PSEN2*) pre-mRNA, which is almost exclusively detected in patients with sporadic AD. An electrophoretic mobility shift assay confirmed aberrant U1 small nuclear ribonucleoprotein particle (snRNP)-HMGA1a complex formation (via the U1-70K component), with RNA containing a specific HMGA1a-binding site and an adjacent 5' splice site. The HMGA1a-induced aberrant exon skipping is caused by impaired dissociation of U1 snRNP from the 5' splice site, leading to a defect in exon definition. 

Kelley *et al.* [57] characterized a kindred with a familial neurodegenerative disorder associated with a mutation in progranulin (*PGRN*). *PGRN* analysis revealed a single base pair deletion in exon 2 (c.154delA), which caused a frameshift (p.Thr52HisfsX2) and, therefore, creation of a premature termination codon and a likely null allele. In this large kindred, most affected individuals had clinical presentations that resembled AD or amnestic mild cognitive impairment associated with a mutation in *PGRN* and underlying frontotemporal lobar degeneration with ubiquitin-positive neuronal cytoplasmic and intranuclear inclusions (FTLD-U). Mutation in the *PGRN* gene can cause frontotemporal dementia (FTD9). Yu *et al.* [58] identified 58 genetic variants that included 26 previously unknown changes. 24 variants appeared to be pathogenic, including eight novel mutations. The frequency of *PGRN* mutations was 6.9% of all FTD-spectrum cases, 21.4% of cases with a pathological diagnosis of FTLD-U, 16.0% of FTD-spectrum cases with a family history of a similar neurodegenerative disease, and 56.2% of cases of FTLD-U with a family history. Haploinsufficiency of *PGRN* is the predominant mechanism leading to FTD.

Polymorphisms within the promoter region of the vascular endothelial growth factor (*VEGF*) gene might elevate the risk for AD. In a Tunisian population, Smach *et al.* [59] found that the distribution of genotype and allele frequencies of the *VEGF* (-2578C/A) and (-1154G/A) polymorphisms did not differ significantly between AD and control groups. In the subgroup of *APOE-4* carriers, the -2578A was observed to be significantly higher in the AD patients than in the control individuals. Endothelin converting enzyme (*ECE-1*) is also a candidate AD susceptibility gene. Individuals homozygous for the C-338A polymorphism (AA) within the *ECE1* gene promoter region are at reduced risk of developing late onset AD (LOAD). A further polymorphism, T-839G, is present within the *ECE1* promoter region but there is no significant association between T-839G and LOAD; however the combined 839T/338A haplotype is associated with decreased risk of LOAD, suggesting that the *ECE1* 338A allele is protective against LOAD in a Chinese population [60].

Down-regulation of somatostatin (SST) expression in the human brain during early stages of aging may lead to an elevation in the steady-state levels of Aβ and therefore may be involved in AD progression. Alterations in the *SST* gene might alter its expression or function and also play a role in the pathogenesis of sporadic AD (SAD). C/T polymorphisms (rs4988514) in the 3' untranslated region of the *SST* gene were screened. The C allele of the rs4988514 polymorphism had an increased incidence in the SAD group compared to the control group in the Chinese population. In subjects with the *APOE-4* allele, the presence of both the CC genotype and the C allele of this polymorphism were elevated in the SAD group compared to the control group. The C allele of the rs4988514 polymorphism may increase the risk for AD in the Chinese population and possibly have additive effect with the *APOE-4* allele [61].

The receptor for advanced glycation end products (RAGE) is associated with several pathological states including AD pathology, while its soluble form (sRAGE) acts as a decoy receptor. Li *et al.* [62] studied a SNP in the *RAGE* gene (G82S; rs2070600), and a SNP associated with increased ligand affinity of *RAGE*. Analysis of a Chinese cohort showed a higher prevalence of the *RAGE* 82S allele and GS + SS genotype in EOAD patients. RAGE contributes to transport of Aβ from the cell surface to the intracellular space. Pretreatment of cultured neurons from wild-type mice with neutralizing antibody to RAGE, and neurons from *Rage* knockout mice displayed decreased uptake of Aβ and protection from Aβ-mediated mitochondrial dysfunction [63]. 

The TAR-DNA binding protein (TDP-43) has been postulated as the disease protein in amyotrophic lateral sclerosis and frontotemporal lobar dementia with ubiquitin-positive inclusions. TDP-43 may also play a role in the pathogenesis of AD. Shibata *et al.* [64] identified an association between a specific haplotype (G-A-A-G) of the *TDP-43* gene and risk for AD. 

Immune dysfunction and aberrant inflammatory reactions are present in AD neuropathology. Neurons express enzymes such as cyclooxygenases (COXs) and 5-lipoxygenase (5-LO) which are considered important in inflammatory cells. It has been suggested that COX-2 and 5-LO enzymes may play a role in the pathophysiology of AD. A significant difference was observed in the distribution of the -765G *COX-2* and -1708A *5-LO* alleles between AD cases and controls. *COX-2* -765G and *5-LO* -1708A alleles were overrepresented in AD patients and underrepresented in controls [65]. The HLA-A*01 variant might also be associated with AD [66]. SNPs in the regulatory regions of the cytokine genes for tumor necrosis factor alpha (*TNFalpha*), interleukin *(IL)-6* and *IL-10* have been suggested to influence the risk of AD with conflicting results. Heterozygotes (AG) or combined genotype (AG + AA) for *IL-10* -1082 were associated with approximately two-fold increase in the risk of AD. Carriers of A alleles of both *TNFalpha*-308 and *IL-10* -1082 had 6.5 times higher risk for AD in comparison with non-carriers. Concomitant presence of both mutant *TNFalpha*-308 A and IL-6 -174 C alleles raised three-fold the AD risk, whereas there was no notable risk for AD afflicted by *IL-6* -174 polymorphism alone [67,68].

Interleukin-33 (IL-33), a newly described member of the IL-1 family, is located on chromosome 9p24, a chromosomal region of interest in AD. Three intronic rs1157505, rs11792633, and rs7044343 SNPs within IL-33 have been reported to be associated with risk of AD in Caucasian and Chinese populations [69]. 

Aromatase gene polymorphisms have also been associated with AD [70]. Polymorphisms in genes encoding amyloid beta-peptide (Aβ)-degrading enzymes neprilysin (NEP) and insulin-degrading enzyme (IDE) individually affect the susceptibility to AD among the Finnish population [71]. Nicastrin (NCSTN) is a type I trans-membrane glycoprotein and an essential component of γ-secretase, a multiprotein complex required for the production of the mature form of Aβ. Overexpression of wild-type *NCSTN* increases Aβ production, indicating that the strict regulation of NCSTN expression may play a fundamental role in the pathogenesis of AD. Zhong *et al.* [72] investigated the effect of a SNP (rs10752637) located in the promoter region of the *NCSTN* gene, on *NCSTN* promoter activity. The distributions of the rs10752637 genotypes and allele frequencies were significantly different between the AD and control groups, with the -922T allele significantly associated with the occurrence of AD. Reporter assays indicated that the rs10752637 -922T allele had a significantly increased promoter activity relative to the -922G allele. The rs10752637 SNP can probably influence the expression of NCSTN, and this may be an influencing factor during the pathogenesis of AD.

The FISH (five SH3 domains) adapter protein and ADAM12 (a disintegrin and metalloprotease) may mediate the neurotoxic effect of Aβ. Both genes are located on chromosome 10, within a region linked to AD (for *SH3PXD2A*) or nearby (for *ADAM12*). Two variants of these genes (rs3740473 for *SH3PXD2A* and rs11244787 for *ADAM12*) have been associated with increased risk for developing AD, but these findings could not be confirmed in different populations [73]. 

Paraoxonase 1 (*PON1*) L55M and Q192R genetic variants might affect individual susceptibility to environmental events, such as exposure to cholinesterase inhibitors. The L55M Met allele exerts an AD risk-enhancing effect only in men, whereas both men and women carrying the M55M/Q192Q genotype exhibit increased survival and later age of onset. These genetic variants are also individually and significantly associated, sometimes in opposite directions for both genders, with beta-amyloid levels, senile plaque accumulation and choline acetyltransferase activity in brain areas [74].

Liu *et al.* [75] studied the potential association of polymorphisms in NMDA receptor subunits NR3A and NR3B, encoded by the *GRIN3A* and *GRIN3B* genes, with AD, on the basis that memantine, an N-methyl-D-aspartate (NMDA) receptor antagonist, may provide some clinical benefit in AD patients. Two SNPs, 3104G/A (rs10989563) and 3723G/A (rs3739722), in the *GRIN3A* gene, and two *GRIN3B* gene polymorphisms, 1210C/T (rs4807399) and 1730C/T (rs2240158), were analyzed. Upon genotyping of the exonic polymorphism in the *GRIN3A* gene, the G allele was present at a higher rate than the A allele at position 3723 in AD patients compared with normal groups. Three haplotypes (designated Ht1-3) were identified from these 2 polymorphisms (3104G/A and 3723G/A), and the distribution of Ht2 (AG) differed between AD patients and controls. The 2 *GRIN3B* gene variants 1210C/T and 1730C/T did not show association with AD. These observations suggest that the genetic variation of the NR3A, but not NR3B, subunit of the NMDA receptor may be a risk factor for AD pathogenesis among the Taiwanese population. di Maria *et al.* [76] reported that the occurrence of delusions and hallucinations in AD is associated with variations in the *G72/DAOA* gene (rs2153674), which is supposed to play a key role in the glutamate pathway regulated through the NMDA receptors. Martínez *et al.* [77] studied the influence of the catechol-*O*-methyltranferase (*COMT*) gene (polymorphism Val158 Met) as a risk factor for AD and mild cognitive impairment of amnesic type (MCI), and its synergistic effect with *APOE* variants in the Basque Country. Neither *COMT* alleles nor genotypes were independent risk factors for AD or MCI; however, the high activity genotypes (GG and AG) showed a synergistic effect with the *APOE-4* allele, increasing the risk of AD. 

Peptidyl-prolyl isomerase, NIMA-interacting 1 (*PIN1*) plays a significant role in the brain and is implicated in numerous cellular processes related to AD and other neurodegenerative conditions. Analysis of 18 *PIN1* common polymorphisms and their haplotypes in EOAD, LOAD and FTD individuals in comparison with the control group did not reveal their contribution to disease risk. In six unrelated familial AD patients four novel *PIN1* sequence variants were detected. The c.58 + 64C > T substitution identified in three patients, was located in an alternative exon. *In silico* analysis suggested that this variant highly increases a potential affinity for a splicing factor and introduces two intronic splicing enhancers. In the peripheral leukocytes of one living patient carrying the variant, a 2.82 fold decrease in PIN1 expression was observed [78]. 

Alzheimer’s and prion diseases are neurodegenerative disorders characterized by the abnormal processing of Aβ peptide and prion protein (PrP^C^), respectively. PrP^C^ may play a critical role in the pathogenesis of AD. PrP^C^ interacts with and inhibits the β-secretase BACE1, the rate-limiting enzyme in the production of Aβ. PrP^C^ was also identified as a receptor for Aβ oligomers and the expression of PrP^C^ appears to be controlled by the amyloid intracellular domain (AICD). PrP^C^ exerts an inhibitory effect on BACE1 to decrease both Aβ and AICD production, and the AICD upregulates PrP^C^ expression, thus maintaining the inhibitory effect of PrP^C^ on BACE1. According to Kellett and Hooper [79], this feedback loop is disrupted in AD, and the increased level of Aβ oligomers binds to PrP^C^ and prevents it from regulating BACE1 activity. It is also likely that *PRNP* gene mutations contribute to AD pathogenesis [8]. 

*HECTD2* maps to 10q and has been implicated in susceptibility to human prion disease. A *HECTD2* intronic tagging SNP, rs12249854 (A/T), has been studied in AD. The rs12249854 minor allele (A) frequency was higher (5.8%) in AD as compared to controls (3.9%). No significant difference was seen in minor allele frequency in the presence or absence of the *APOE-4* allele. According to these results, it appears that the common haplotypes of *HECTD2*, tagged by rs12249854, are not associated with susceptibility to LOAD [80].

Ubiquitin-conjugating enzyme E2I (Ubc9) ligates small ubiquitin-related modifier (SUMO) to target proteins, resulting in changes of their localization, activity, or stability. Sumoylation of APP was reported to be associated with decreased levels of Aβ aggregates, suggesting that sumoylation may play a role in the pathogenesis of AD. Ahn *et al.* [81] investigated the association between genetic variations of Ubc9 gene (*UBE2I*) and LOAD in Koreans. The genotype distribution of a polymorphism in intron 7 (rs761059) differed between AD cases and controls and one haplotype (ht2 CAGAG) was found in 14.0% of the AD patients and in 11.1% of the controls. Stratification by the *APOE-4* allele gave no significant difference between the groups. When the samples were stratified by gender, the genotypes of two SNPs (rs8052688, rs8063) were significantly associated with the risk of MCI among women. 

To gain insights into the evolution of the regulatory mechanisms of the aged brain, Persengiev *et al.* [82] compared age-related differences in microRNA (miRNA) expression levels in the cortex and cerebellum of humans, chimpanzees and rhesus macaques on a genome-wide scale. In contrast to global miRNA downregulation, a small subset of miRNAs was found to be selectively upregulated in the aging brain of all three species. miR-144 appeared to be associated with the aging progression. miR-144 plays a central role in regulating the expression of ataxin 1 (*ATXN1*), a gene which is associated with spinocerebellar ataxia type 1 (SCA1). miRNA activity, including miR-144, -101 and -130 processing, was increased in the cerebellum and cortex of SCA1 and Alzheimer’s patients relative to healthy aged brains. The activation of miRNA expression in the aging brain might serve to reduce the cytotoxic effect of polyglutamine expanded *ATXN1* and the deregulation of miRNA expression might be a risk factor for neurodegeneration. Bettens *et al.* [83] also obtained evidence for association between rs179943, an intronic SNP in *ATXN1* at 6p22.3, and AD. 

The cholesterol transporter ATP-binding cassette transporter A1 (ABCA1) moves lipids onto apolipoproteins including ApoE. Donkin *et al.* [84] reported that in amyloid mouse models of AD, ABCA1 deficiency exacerbates amyloidogenesis, whereas ABCA1 overexpression ameliorates amyloid load, suggesting a role for ABCA1 in Abeta metabolism. Agonists of Liver X Receptors (LXR), including GW3965, induce transcription of several genes including *ABCA1* and *APOE*, reduce Abeta levels and improve cognition in AD mice. Treatment of APP/PS1 mice with GW3965 increased ABCA1 and ApoE protein levels. ABCA1 was observed to require significantly elevated ApoE levels in brain tissue and CSF upon GW3965 treatment. APP/PS1 mice treated with either 2.5 mg/kg/d or 33 mg/kg/d of GW3965 showed a clear trend toward reduced amyloid burden in hippocampus and whole brain, whereas treated APP/PS1 mice lacking ABCA1 failed to display reduced amyloid load in whole brain and showed trends toward increased hippocampal amyloid. Treatment of APP/PS1 mice with either dose of GW3965 completely restored novel object recognition (NOR) memory to wild-type levels, which required ABCA1. These results reported by Donkin and co-workers suggest that ABCA1 contributes to several beneficial effects of the LXR agonist GW3965 in APP/PS1 mice.

The phospholipid transfer protein (PLTP) reduces phosphorylation of tau in human neuronal cells. Patients with AD have significantly higher levels of PLTP in brain tissue and significantly lower PLTP-mediated phospholipid transfer activity in cerebrospinal fluid. PLTP also affects ApoE secretion from glial cells. Kuerban *et al.* [85] investigated whether SNPs of the PLTP gene are associated with AD in the Japanese population, and found no genetic association between PLTP and AD. 

Genome-wide association studies (GWAS) in AD highlight over two dozen novel potential susceptibility loci beyond the well-established *APOE* association, including *GAB2* (GRB2-associated binding protein 2), galanin-like peptide (*GALP*), piggyBac transposable element derived 1 (*PGBD1*), tyrosine kinase, non-receptor 1 (*TNK1*), and at least three replicated loci in hitherto uncharacterized genomic intervals on chromosomes 14q32.13, 14q31.2 and 6q24.1, probably implicating the existence of novel AD genes in these regions [86]. 

(c) Diverse mutations located in mitochondrial DNA (mtDNA) through heteroplasmic transmission can influence aging and oxidative stress conditions, conferring phenotypic heterogeneity [8,87]. The human presequence protease (hPreP) was recently shown to be the major mitochondrial Aβ-degrading enzyme. Genetic variation in the hPreP gene *PITRM1* has been investigated by Pinho *et al.* [88]. No genetic association between any of the SNPs and the risk for AD was found; however, functional analysis of four non-synonymous SNPs in *hPreP* revealed a decreased activity compared to wild-type *hPreP*. Using Aβ, the presequence of ATP synthase F_1_β subunit and a fluorescent peptide as substrates, the lowest activity was observed for the *hPreP*(A525D) variant, corresponding to rs1224893, which displayed only 20–30% of wild-type activity. Genetic variation in the hPreP gene *PITRM1* might contribute to mitochondrial dysfunction in AD. Recent data suggest the possible contribution of heme deficiency to the progressive derangement of mitochondria in AD brain; shortage of heme, and particularly of heme-a, actually leads to loss of mitochondrial cytochrome c oxidase (COX), abnormal production of reactive oxygen species and altered amyloid precursor protein metabolism. Differences in the amount and/or functioning of COX assembly subunit 10 (COX10) and 15 (COX15), the key enzymes involved in heme-a biosynthesis, could be linked to variations of the individual risk to develop AD. Vitali *et al.* [89] analyzed mRNA expression in the hippocampus from AD patients and controls, as well as nucleotide variations in DNA sequences in AD. *COX15* mRNA was significantly more abundant in the cerebral tissue of AD patients, and the IVS-178G > A SNP in *COX10* and the c + 1120C > T SNP in *COX15* were significantly less represented in AD, suggesting a possible protective role.

Japanese AD patients are associated with the haplogroups G2a, B4c1, and N9b1. Takasaki [90] compared mitochondrial haplogroups of AD patients with those of other classes of Japanese (centenarians, Parkinson’s disease (PD), type 2 diabetes mellitus (T2D), and non-obese young males). The four classes of people were associated with the following haplogroups: Japanese centenarians with M7b2, D4b2a, and B5b; PD patients with M7b2, B4e, and B5b; T2D patients with B5b, M8a1, G, D4, and F1; and Japanese healthy non-obese young males with D4g and D4b1b. The haplogroups of the AD patients differed from those of the other four categories. 

Santoro *et al.* [91] applied for the first time a high resolution analysis to investigate the possible association between mtDNA-inherited sequence variation and AD in 936 AD patients and 776 cognitively assessed normal controls from central and northern Italy. Among over 40 mtDNA sub-haplogroups analyzed, they found that sub-haplogroup H5 is a risk factor for AD, particularly in females, independently of the *APOE* genotype. The H5a subgroup of molecules, harboring the 4336 transition in the tRNAGln gene was about threefold more represented in AD patients than in controls (2.0% *vs.* 0.8%), and it might account for the increased frequency of H5 in AD patients (4.2% *vs.* 2.3%). The complete re-sequencing of the 56 mtDNAs belonging to H5 revealed that AD patients showed a trend towards a higher number of sporadic mutations in tRNA and rRNA genes when compared with controls.

### 2.2. Gene Interactions

Although *APP* and *PSEN* mutations are considered causative factors for AD, the total number of mutations identified in the *APP*, *PSEN1* and *PSEN2* genes account for less than 3% of the cases with AD, clearly indicating that neurodegeneration associated with AD pathogenesis cannot be exclusively attributed to *APP/PSEN*-related cascades (amyloid hypothesis). Alterations in the ubiquitin-proteasome system and biochemical disarray in the chaperone machinery are alternative and/or complementary pathogenic events potentially leading to defects in protein synthesis, folding, and degradation with subsequent conformational changes, aggregation, and accumulation in cytotoxic deposits [8,11]. A more plausible explanation would seem to be that multiple susceptibility SNPs with a very subtle genetic variation cooperatively contribute, in concert with environmental factors and concomitant CNS vulnerability, to premature neurodegeneration in dementia. 

We have compared the distribution and frequency of major polymorphic variants of different genes potentially associated with AD (*i.e.*, *APOE*, *PSEN1*, *A2M*-V1001, *A2M*-I/D, *ACE*, *FOS*, *AGT*-235, *AGT*-174, *eNOS3*-E298D, *eNOS3*-27bpTR, *CETP*, *MTHRF*) in the general population, in adults (>45 years) with no family history of dementia, and in patients with dementia, and could not find any significant differences among the three groups except in the case of the *APOE* gene, which exhibits a clear accumulation of *APOE-3/4* and *APOE-4/4* genotypes (overload of the *APOE-4* allele) in AD cases [7]. If we consider that a genetic variation higher than 2% could be of significant value, then several polymorphisms clearly differ in AD as compared with the other two population clusters, including the *PSEN1*-1/2, *ACE*-D/D, *ACE*-I/I, *CEPT*-B1/B1, and *MTHFR*-T/T polymorphisms [7]. 

Defective functions of genes associated with longevity may influence premature neuronal survival, since neurons are potential pacemakers defining life span in mammals [8,16]. Hypothalamic expression of CREB-binding protein (*CBP*) and CBP-binding partner Special AT-rich sequence binding protein 1 (*SATB-1*) is highly correlated with lifespan across five strains of mice, and expression of these genes decreases with age and diabetes in mice. In a transgenic Aβ42 model of AD, cbp-1 RNAi prevents protective effects of bacterial dilution (bDR) and accelerates Aβ42-related pathology. Consistent with the function of CBP as a histone acetyltransferase, drugs that enhance histone acetylation increase lifespan and reduce Aβ42-related pathology, protective effects completely blocked by cbp-1 RNAi. Other factors implicated in lifespan extension are also *CBP*-binding partners, suggesting that *CBP* constitutes a common factor in the modulation of lifespan and disease burden by DR and the insulin/*IGF1* signaling pathway [92].

AD patients have been reported to have shorter telomeres in peripheral blood leukocytes (PBLs) than age-matched control subjects. However, it is unclear if PBL telomere length reflects brain telomere length, which might play a more direct role in AD pathogenesis. Lukens *et al.* [93] examined the correlation between PBL and cerebellum telomere length in AD patients.The PBL and cerebellum telomere lengths were directly correlated in individuals with AD. Nonetheless, cerebellum telomere lengths were not significantly different in AD patients and age-matched control subjects. Reduced PBL telomere length in AD might not reflect reduced telomere length in bulk brain tissue, but may be a marker of changes in a subset of brain tissues or other tissues that affect the pathogenesis of AD. Zekry *et al.* [94] evaluated the usefulness of telomere length alone or combined with APOE polymorphism in diagnosing mild cognitive impairment (MCI) and in differentiating AD from vascular and mixed dementia. Although APOE-4 was associated with dementia, no significant differences in telomere length were found among patients with different types of dementia. The combination of telomere length and APOE-4 did not confer a significantly higher dementia risk [94]. 

### 2.3. Functional Genomics

Over 80% of the genes which conform the structural architecture of the human genome are expressed in the brain in a time-dependent manner along the lifespan. The cellular complexity of the CNS (with 10^3^ different cell types) and synapses (with each of the 10^11^ neurons in the brain having around 10^3^–10^4^ synapses with a complex multiprotein structure integrated by 10^3^ different proteins) requires a very powerful technology for gene expression profiling, which is still in its very early stages and is not devoid of technical obstacles and limitations [95]. Transcripts of 16,896 genes have been measured in different CNS regions. Each region possesses its own unique transcriptome fingerprint which is independent of age, gender and energy intake. Less than 10% of genes are affected by age, diet or gender, with most of these changes occurring between middle and old age. Gender and energy restriction have robust influences on the hippocampal transcriptome of middle-aged animals. Prominent functional groups of age- and energy-sensitive genes are those encoding proteins involved in DNA damage responses, mitochondrial and proteasome functions, cell fate determination and synaptic vesicle trafficking. The systematic transcriptome dataset provides a window into mechanisms of neuropathogenesis and CNS vulnerability [96]. 

Functional genomics studies have demonstrated the influence of many genes on AD pathogenesis and phenotype expression. The study of genotype-phenotype correlations is essential for the evaluation of the actual impact of specific polymorphic variants of a particular gene on the clinical manifestation of the disease and/or biological markers reflecting the disease condition or different biological states of the individual. Mutations in the *APP*, *PSEN1*, *PSEN2*, and *MAPT* genes give rise to well-characterized differential neuropathological and clinical phenotypes of dementia [8,20,21]. *APP* mutations are associated with AD1, early-onset progressive autosomal recessive dementia, early-onset AD with cerebral amyloid angiopathy, and hereditary amyloidosis with cerebral hemorrhage Dutch type, Italian type, or Iowa type. *PSEN1* mutations are associated with the phenotypes of familial AD3, familial AD3 with unusual plaques, familial AD with spastic paraparesis and unusual plaques, familial AD with paraparesias and apraxia, frontotemporal dementia, Pick’s disease, and dilated cardiomyopathy. *MAPT* mutations are associated with frontotemporal dementia, frontotemporal dementia with parkinsonism, Pick’s disease, progressive supranuclear palsy, progressive atypical supranuclear palsy, tauopathy and respiratory failure [8]. 

Transgenic animals also reproduce to some extent the neuropathological hallmarks of AD in a sequential manner. The triple transgenic mouse model of AD (3xTg-AD) harbors three AD-related loci: human PS1M146V, human APPswe, and human MAPTP301L. These animals develop both amyloid plaques and NFT-like pathology in a progressive and age-dependent manner in hippocampus, amygdala, and cerebral cortex, the main foci of human AD neuropathology. The evolution of AD-related transgene expression, amyloid deposition, tau phosphorylation, astrogliosis, and microglia activation throughout the hippocampus, entorhinal cortex, primary motor cortex, and amygdala over a 26-month period has been immunohistochemically documented. Intracellular Aβ accumulation is the earliest of AD-related pathologies to be detectable, followed temporally by phospho-tau, extracellular Aβ, and finally paired helical filament and NFT pathology [97]. In the same model, a decrease in neurogenesis directly associated with the presence of amyloid plaques and an increase in the number of Aβ containing neurons in the hippocampus has been demonstrated [98].

Different *APOE* genotypes also confer specific phenotypic profiles to AD patients. Some of these profiles may add risk or benefit when the patients are treated with conventional drugs, and in many instances the clinical phenotype demands the administration of additional drugs which increase the complexity of therapeutic protocols. From studies designed to define *APOE*-related AD phenotypes [6,7,8,9,10,11,12,13,14,15,16,17,99,100,101,102,103,104], several confirmed conclusions can be drawn: (i) the age-at-onset is 5-10 years earlier in approximately 80% of AD cases harboring the *APOE-4/4* genotype; (ii) the serum levels of ApoE are lowest in *APOE-4/4*, intermediate in *APOE-3/3* and *APOE-3/4*, and highest in *APOE-2/3* and *APOE-2/4*; (iii) serum cholesterol levels are higher in *APOE-4/4* than in the other genotypes; (iv) HDL-cholesterol levels tend to be lower in *APOE-3* homozygotes than in *APOE-4* allele carriers; (v) LDL-cholesterol levels are systematically higher in *APOE-4/4* than in any other genotype; (vi) triglyceride levels are significantly lower in *APOE-4/4*; (vii) nitric oxide levels are slightly lower in *APOE-4/4*; (viii) serum Aβ levels do not differ between *APOE-4/4* and the other most frequent genotypes (*APOE-3/3*, *APOE-3/4*); (ix) blood histamine levels are dramatically reduced in *APOE-4/4* as compared with the other genotypes; (x) brain atrophy is markedly increased in *APOE-4/4* > *APOE-3/4* > *APOE-3/3*; (xi) brain mapping activity shows a significant increase in slow wave activity in *APOE-4/4* from early stages of the disease; (xii) brain hemodynamics, as reflected by reduced brain blood flow velocity and increased pulsatility and resistance indices, is significantly worse in *APOE-4/4* (and in *APOE-4* carriers, in general, as compared with *APOE-3* carriers); (xiii) lymphocyte apoptosis is markedly enhanced in *APOE-4* carriers; (xiv) cognitive deterioration is faster in *APOE-4/4* patients than in carriers of any other *APOE* genotype; (xv) occasionally, in approximately 3-8% of the AD cases, the presence of some dementia-related metabolic dysfunctions (e.g., iron, folic acid, vitamin B_12_ deficiencies) accumulate more in *APOE-4* carriers than in *APOE-3* carriers; (xvi) some behavioral disturbances (bizarre behaviors, psychotic symptoms), alterations in circadian rhythm patterns (e.g., sleep disorders), and mood disorders (anxiety, depression) are slightly more frequent in *APOE-4* carriers; (xvii) aortic and systemic atherosclerosis is also more frequent in *APOE-4* carriers; (xviii) liver metabolism and transaminase activity also differ in *APOE-4/4* with respect to other genotypes; (xix) blood pressure (hypertension) and other cardiovascular risk factors also accumulate in *APOE-4*; and (xx) *APOE-4/4* are the poorest responders to conventional drugs. These 20 major phenotypic features clearly illustrate the biological disadvantage of *APOE-4* homozygotes and the potential consequences that these patients may experience when they receive pharmacological treatment [6,7,8,9,10,11,12,13,14,15,16,17,25,99,100,101,102,103,104,105,106,107].

## 3. Dementia Phenotype and Biomarkers

The phenotypic features of the disease represent the biomarkers to be modified with an effective therapeutic intervention. Important differences have been found in the AD population as compared with healthy subjects in different biological parameters, including blood pressure, glucose, cholesterol and triglyceride levels, transaminase activity, hematological parameters, metabolic factors, thyroid function, brain hemodynamic parameters, and brain mapping activity [6,7,8,11,12,13,14,15,16]. Blood pressure values, glucose levels and cholesterol levels are higher in AD than in healthy elderly subjects. Approximately 20% of AD patients are hypertensive, 25% are diabetics, 50% are hypercholesterolemic, and 23% are hypertriglyceridemic. Over 25% of the patients exhibit high GGT activity, 5–10% show anemic conditions, 30–50% show an abnormal cerebrovascular function characterized by poor brain perfusion, and over 60% have an abnormal electroencephalographic pattern, especially in frontal, temporal, and parietal regions, as revealed by quantitative EEG (qEEG) or computerized mapping [6,7,8]. Significant differences are currently seen between females and males, indicating the effect of gender on the phenotypic expression of the disease. In fact, the prevalence of dementia is 10–15% higher in females than in males from 65 to 85 years of age. All these parameters are highly relevant when treating AD patients because some of them reflect a concomitant pathology which also needs therapeutic consideration. They can also represent general biomarkers together with regional brain atrophy and perfusion and cognitive function, which may serve as therapeutic outcome measures. Other biomarkers of potential interest include cerebrospinal fluid (CSF) and peripheral levels of Aβ42, protein tau, histamine, interleukins, and some other candidate markers [7,108,109]. In proteomic studies, several candidate CSF protein biomarkers have been assessed in neuropathologically confirmed AD, non-demented (ND) elderly controls and non-AD dementias (NADD). Markers selected included apolipoprotein A-1 (ApoA1), hemopexin (HPX), transthyretin (TTR), pigment epithelium-derived factor (PEDF), Aβ1-40, Aβ1-42, total tau, phosphorylated tau, α-1 acid glycoprotein (A1GP), haptoglobin, zinc α-2 glycoprotein (Z2GP) and apolipoprotein E (ApoE). The concentrations of Aβ1-42, ApoA1, A1GP, ApoE, HPX and Z2GP differed significantly among AD, ND and NADD subjects. The CSF concentrations of these three markers distinguished AD from ND subjects with 84% sensitivity and 72% specificity, with 78% of subjects correctly classified. By comparison, using Aβ1-42 alone gave 79% sensitivity and 61% specificity, with 68% of subjects correctly classified. For the diagnostic discrimination of AD from NADD, only the concentration of Aβ1-42 was significantly related to diagnosis, with a sensitivity of 58% and a specificity of 86% [110]. 

## 4. Therapeutic Strategies in Dementia

Modern therapeutic strategies in AD are addressed to interfering with the main pathogenic mechanisms potentially involved in AD. Major pathogenic events (drug targets) and their respective therapeutic alternatives include the following: genetic defects, β-amyloid deposition, tau-related pathology, apoptosis, neurotransmitter deficits, neurotrophic deficits, neuronal loss, neuroinflammation, oxidative stress, calcium dysmetabolism, neuronal hypometabolism, lipid metabolism dysfunction, cerebrovascular dysfunction, neuronal dysfunction associated with nutritional and/or metabolic deficits, and a miscellany of pathogenic mechanisms potentially manageable with diverse classes of chemicals or biopharmaceuticals [6,7,8,9,10,11,12,13,14,15,16,17,99,104]. Since the early 1980s, the neuropharmacology of AD was dominated by the acetylcholinesterase inhibitors, represented by tacrine, donepezil, rivastigmine, and galantamine [4,5,111]. Memantine, a partial NMDA antagonist, was introduced in the 2000s for the treatment of severe dementia [112]; and the first clinical trials with immunotherapy, to reduce amyloid burden in senile plaques, were withdrawn due to severe ADRs [113,114]. During the past few years no relevant drug candidates have been postulated for the treatment of AD, despite the initial promises of β- and γ-secretase inhibitors [11,115,116]. However, assuming that the best treatment for AD is neuronal death prevention prior to the onset of the disease, novel therapeutic options and future candidate drugs for AD might be a new generation of anti-amyloid vaccines, such as DNA Aβ42 trimer immunization [117], heterocyclic indazole derivatives [inhibitors of the serum- and glucocorticoid-inducible-kinase 1 (SGK1)] [118], NSAID-like compounds [119], IgG-single chain Fv fusion proteins [120], Hsp90 inhibitors and HSP inducers [121], inhibitors of class I histone deacetylases [122], some phenolic compounds [123], agonists of the peroxisome proliferator activated receptor gamma (PPARgamma) [124], microRNAs [125], and gene silencing (RNAi) [126]. 

## 5. Pharmacogenomics

Pharmacogenetics/pharmacogenomics relates to the application of genomic technologies, such as genotyping, gene sequencing, gene expression, genetic epidemiology, transcriptomics, proteomics, metabolomics and bioinformatics, to drugs in clinical development and on the market, applying the large-scale systematic approaches of genomics to speed up the discovery of drug response markers, whether they act at the level of drug target, drug metabolism, or disease pathways [7,15,16,17,127]. 

The potential implications of pharmacogenomics in clinical trials and molecular therapeutics is that a particular disease could be treated according to genomic and biological markers, selecting medications and diseases which are optimized for individual patients or clusters of patients with a similar genomic profile. For many medications, interindividual differences are mainly due to SNPs in genes encoding drug metabolizing enzymes, drug transporters, and/or drug targets (e.g., genome-related defective enzymes, receptors and proteins, which alter metabolic pathways leading to disease phenotype expression). 

The application of these procedures to CNS disorders is an extremely difficult task, since most neuropsychiatric diseases are complex disorders in which many different genes might be involved [6]. In addition, it is very unlikely that a single drug would be able to reverse the multifactorial mechanisms associated with neuronal dysfunction in most CNS processes with a complex phenotype affecting mood, personality, behavior, cognition, and functioning. This heterogeneous clinical picture usually requires the utilization of different drugs administered simultaneously. This is particularly important in the elderly population. In fact, the average number of drugs taken by patients with dementia ranges from 6 to over 10 per day depending upon their physical and mental conditions. Nursing home residents receive, on average, 7-8 medications each month, and over 30% of residents have monthly drug regimes of nine or more medications, including (in descending order) analgesics, antipyretics, gastrointestinal agents, electrolytic and caloric preparations, CNS agents, anti-infective agents, and cardiovascular agents [128]. In population-based studies, over 35% of patients older than 85 years are moderate or chronic antidepressant users [129]. Polypharmacy, drug-drug interactions, adverse reactions, and non-compliance are substantial therapeutic problems in the pharmacological management of elderly patients [130], adding further complications and costs to the patients and their caregivers. Over 25% of elderly individuals receive at least one of more than 30 potentially inappropriate medications in 10 health maintenance organizations (HMOs) of the USA [131]. Although drug effect is a complex phenotype which depends on many factors, it is estimated that genetics accounts for 20% to 95% of variability in drug disposition and pharmacodynamics [132]. Under these circumstances, therapeutics optimization is a major goal in neuropsychiatric disorders and in the elderly population, and novel pharmacogenetic and pharmacogenomic procedures may help in this endeavour [6,7,8,9,10,11,12,13,14,15,16,25,99,100,101,102,103,104,133].

The pharmacogenomic outcome depends upon many different determinant factors including (i) genomic profile; (ii) disease phenotype; (iii) concomitant pathology; (iv) genotype-phenotype correlations; (v) nutritional conditions; (vi) age and gender; (vii) pharmacological profile of the drugs; (viii) drug-drug interactions; (ix) gene expression profile; (x) transcriptomic cascade; (xi) proteomic profile; and (xii) metabolomic networking. The dissection and further integration of all these factors is of paramount importance for the assessment of the pharmacogenomic outcome in terms of safety and efficacy. Pharmacogenomic approaches based on genomewide sets of SNPs associated with drug response are now feasible and may offer the potential to personalize therapeutics [7].

The vast majority of drugs in current use, and many psychotropics, are metabolized by enzymes known to be genetically variable, including: (i) esterases: butyrylcholinesterase, paraoxonase/arylesterase; (ii) transferases: N-acetyltransferase, sulfotransferase, thiol methyltransferase, thiopurine methyltransferase, catechol-*O*-methyltransferase, glutathione-S-transferases, UDP-glucuronosyltransferases, glucosyltransferase, histamine methyltransferase; (iii) reductases: NADPH: quinine oxidoreductase, glucose-6-phosphate dehydrogenase; (iv) oxidases: alcohol dehydrogenase, aldehydehydrogenase, monoamine oxidase B, catalase, superoxide dismutase, trimethylamine *N*-oxidase, dihydropyrimidine dehydrogenase; and (v) cytochrome P450 enzymes, such as CYP1A1, CYP2A6, CYP2C8, CYP2C9, CYP2C19, CYP2D6, CYP2E1, CYP3A5 and many others [6,7,16]. Polymorphic variants in the genes encoding these enzymes can induce alterations in drug metabolism modifying the efficacy and safety of the prescribed drugs.

Drug metabolism includes phase I reactions (*i.e.*, oxidation, reduction, hydrolysis) and phase II conjugation reactions (*i.e.*, acetylation, glucuronidation, sulphation, methylation). The principal enzymes with polymorphic variants involved in phase I reactions are the following: CYP3A4/5/7, CYP2E1, CYP2D6, CYP2C19, CYP2C9, CYP2C8, CYP2B6, CYP2A6, CYP1B1, CYP1A1/2, epoxide hydrolase, esterases, NQO1 (NADPH-quinone oxidoreductase), DPD (dihydropyrimidine dehydrogenase), ADH (alcohol dehydrogenase), and ALDH (aldehyde dehydrogenase). Major enzymes involved in phase II reactions include the following: UGTs (uridine 5'-triphosphate glucuronosyl transferases), TPMT (thiopurine methyltransferase), COMT (catechol-O-methyltransferase), HMT (histamine methyl-transferase), STs (sulfotransferases), GST-A (glutathione S-transferase A), GST-P, GST-T, GST-M, NAT_S_ (N-acetyl transferases), and others [6,7,11,16]. 

### 5.1. Pharmacogenetics of Psychotropic Drugs

Historically, the vast majority of pharmacogenetic studies of CNS disorders have been addressed to evaluate the impact of cytochrome P450 enzymes on drug metabolism. Conventional targets for psychotropic drugs were the neurotransmitters dopamine, serotonin, noradrenaline, GABA, ion channels, acetylcholine and their respective biosynthetic and catalyzing enzymes, receptors and transporters [134]; however, in the past few years many different genes have been associated with both pathogenesis and pharmacogenomics of neuropsychiatric disorders. Some of these genes and their products constitute potential targets for future treatments. New developments in genomics, including whole genome genotyping approaches and comprehensive information on genomic variation across populations, coupled with large-scale clinical trials in which DNA collection is routine, now provide the impetus for a next generation of pharmacogenetic studies and identification of novel candidate drugs [135,136,137].

The typical paradigm for the pharmacogenetics of phase I drug metabolism is represented by the cytochrome P-450 enzymes, a superfamily of microsomal drug-metabolizing enzymes. P450 enzymes comprise a superfamily of heme-thiolate proteins widely distributed in bacteria, fungi, plants and animals. The P450 enzymes are encoded in genes of the CYP superfamily and act as terminal oxidases in multicomponent electron transfer chains which are called P450-containing monooxigenase systems. Some of the enzymatic products of the CYP gene superfamily can share substrates, inhibitors and inducers whereas others are quite specific for their substrates and interacting drugs.

The microsomal, membrane-associated, P450 isoforms CYP3A4, CYP2D6, CYP2C9, CYP2C19, CYP2E1, and CYP1A2 are responsible for the oxidative metabolism of over 90% of marketed drugs. About 60–80% of the psychotropic agents currently used for the treatment of neuropsychiatric disorders are metabolized via enzymes of the CYP family, especially CYP1A2, CYP2B6, CYP2C8/9, CYP2C19, CYP2D6 and CYP3A4. CYP3A4 metabolizes more drug molecules than all other isoforms together. Most of these polymorphisms exhibit geographic and ethnic differences [138,139,140,141]. These differences influence drug metabolism in different ethnic groups in which drug dosage should be adjusted according to their enzymatic capacity, differentiating normal or extensive metabolizers (EMs), intermediate metabolizers (IMs), poor metabolizers (PMs) and ultrarapid metabolizers (UMs). 

Most drugs act as substrates, inhibitors or inducers of CYP enzymes. Enzyme induction enables some xenobiotics to accelerate their own biotransformation (auto-induction) or the biotransformation and elimination of other drugs. A number of P450 enzymes in the human liver are inducible. Induction of the majority of P450 enzymes occurs by an increase in the rate of gene transcription and involves ligand-activated transcription factors, aryl hydrocarbon receptor, constitutive androstane receptor (CAR), and pregnane X receptor (PXR) [142,143]. In general, binding of the appropriate ligand to the receptor initiates the induction process that cascades through a dimerization of the receptors, their translocation to the nucleus and binding to specific regions in the promoters of CYPs [143]. CYPs are also expressed in the CNS, and a complete characterization of constitutive and induced CYPs in the brain is essential for understanding the role of these enzymes in neurobiological functions and in age-related and xenobiotic-induced neurotoxicity [144]. *CYP2D6* mRNA expression is detected in all regions of the human brain where it may be involved in the metabolism of amines and steroids and in the regulation of diverse CNS activities [145]. 

There are substantial differences between individuals in the effects of psychotropic drugs in the treatment of neuropsychiatric disorders. Pharmacogenetic studies of psychotropic drug response have focused on determining the relationship between variation in specific candidate genes and the positive and adverse effects of drug treatment [134,146,147]. Approximately 18% of neuroleptics are major substrates of CYP1A2 enzymes, 40% of CYP2D6, and 23% of CYP3A4; 24% of antidepressants are major substrates of CYP1A2 enzymes, 5% of CYP2B6, 38% of CYP2C19, 85% of CYP2D6, and 38% of CYP3A4; 7% of benzodiazepines are major substrates of CYP2C19 enzymes, 20% of CYP2D6, and 95% of CYP3A4 [6,7]. About 80% of patients with resistant depression, 60% of patients non-responsive to neuroleptics, and 50–70% of patients with paradoxical responses to benzodiazepines are carriers of mutant variants of the *CYP2D6*, *CYP2C9* and *CYP3A4* genes, falling within the categories of poor or ultra-rapid metabolizers [7].

The clinical impact of the cytochrome P450 (CYP) enzyme CYP2D6 poor metabolizer (PM) genotype in patients taking antipsychotic medication has been investigated in a retrospective study. The impaired metabolic capacity of the PM genotype results in higher steady-state plasma concentrations at a given dose, thus increasing the risk of toxic effects from medication. Extrapyramidal syndrome or tardive dyskinesia (EPS/TD) was significantly more frequent among PM patients than among the matched IM and EM control subjects. This finding was further supported by the significantly higher prevalence of noncompliance among the same PM patients. Genetically encoded differences in the rate of drug metabolism through CYP2D6 can predict antipsychotic side-effects and prompts the question of whether genotyping early in the course of illness to facilitate adjustment of pharmacotherapy will improve treatment outcomes and reduce side-effects [148]. 

The effects of the *CYP2D6* and *CYP3A5* genotypes on the steady-state plasma levels of risperidone (RIS), 9-hydroxyrisperidone (9-OH-RIS), and the active moiety (RIS plus 9-OH-RIS) were studied in schizophrenic patients. The patients investigated were *CYP2D6* extensive metabolizers (EMs; CYP2D6*1/*1, *1/*10, and *10/*10) and *CYP2D6* poor metabolizers (PMs; CYP2D6*1/*5 and *10/*5). For the *CYP3A5* genotype, patients were CYP3A5*1 expressors (*1/*1 and *1/*3) and *CYP3A5* nonexpressors (*3/*3). The plasma levels of RIS (2.03 ng/mL per milligram for EMs *vs.* 5.57 ng/mL per milligram for PMs) and 9-OH-RIS (5.06 ng/mL per milligram for EMs *vs.* 0.22 ng/mL per milligram for PMs) were significantly different among *CYP2D6* genotype groups, but the *CYP2D6* EMs (7.09 ng/mL per milligram) and PMs (5.79 ng/mL per milligram) did not show differences in the levels of the active moiety. *CYP3A5* nonexpressors exhibited higher plasma concentrations of both RIS and 9-OH-RIS than its expressors. In the case of 9-OH-RIS, *CYP3A5* nonexpressors exhibited significantly higher concentrations than *CYP3A5* expressors. Concentrations of the active moiety were also significantly different between the *CYP3A5* nonexpressors and expressors. According to these results reported by Kang *et al.* [149], both *CYP2D6* and *CYP3A5* genotypes affect plasma levels of RIS and 9-OH-RIS, whereas the active moiety levels are influenced only by the *CYP3A5* genotype but not by the *CYP2D6* genotype [149]. 

Risperidone is converted to 9-hydroxyrisperidone by CYP2D6. The CYP2D6*10 polymorphism, which is a prevalent mutant allele among East Asians, and the presence of co-medication exert significant influences on the pharmacokinetics of risperidone [150].

Some studies attempt to determine whether testing for cytochrome P450 (CYP) polymorphisms in adults entering antipsychotic treatment for schizophrenia (SCZ) leads to improvement in outcomes, is useful in medical, personal or public health decision-making, and is a cost-effective use of health-care resources [151].

Targets that show promise for pharmacologic focus in SCZ and psychosis include the dopamine receptors (especially D_1_) in the prefrontal cortex, the serotonin receptors in the prefrontal cortex and anterior cingulate cortex, the glutamatergic excitatory synapse, the acetylcholine nicotinic receptors in the hippocampus, the acetylcholine muscarinic receptors, and the brain gamma-aminobutyric acid (GABA) system [152,153]. In addition to cytochrome P450 enzymes, many other gene products influence both efficacy and safety of psychotropic drugs [6,7,134,137,154,155,156]. 

To detect potential predictor gene variants for risperidone response in schizophrenic subjects, Ikeda *et al.* [157] performed a convergent analysis based on (i) a genomewide (100K SNP) SNP pharmacogenetic study of risperidone response and (ii) a global transcriptome study of genes with mRNA levels influenced by risperidone exposure in mouse prefrontal cortex. Fourteen genes were highlighted as of potential relevance to risperidone activity in both studies: *ATP2B2*, *HS3ST2*, *UNC5C*, *BAG3*, *PDE7B*, *PAICS*, *PTGFRN*, *NR3C2*, *ZBTB20*, *ST6GAL2*, *PIP5K1B*, *EPHA6*, *KCNH5*, and *AJAP1*. 

Analysis of polymorphic variants in 5-HT2A receptors (*5-HT2AR*-A-1438G) revealed that schizophrenic carriers of the G/G genotype receiving olanzapine showed a significant tendency toward improvement in the PANSS positive syndrome score in comparison with patients who did not have a G gene (AA and AG) [158]. 

Human 5-HT7 receptor may be involved in the pharmacodynamics of risperidone and may influence clinical response of the drug. A pharmocogenetics study of this receptor may therefore be useful in developing individualized therapy; however, no significant correlation of *HTR7* with antipsychotic efficacy was detected in either genotype or haplotype analysis in the Chinese population [159]. 

Aripiprazole acts as a partial agonist at dopamine D_2_ (DRD2) and D_3_ (DRD3) and serotonin 1A (HTR1A) receptors and as an antagonist at serotonin 2A receptors (HTR2A) [160]. Since aripiprazole acts as an antagonist at HTR2A, genetic variants of *HTR2A* may be important in explaining variability in response to aripiprazole. The GG/CC genotype group of *HTR2A* A-1438G/T102C polymorphisms predicts poor aripiprazole response specifically for negative symptoms. In addition, the clinical factors, including dosage of aripiprazole, age, duration of illness, and diagnostic subtype, were found to influence PANSS (Positive and Negative Syndrome Scale) performance after aripiprazole treatment [161].

Chen *et al.* [160] investigated whether the efficacy of aripiprazole can be predicted by a functional *DRD3* gene polymorphism Ser9Gly (rs6280) as modified by clinical factors in Han Chinese hospitalized patients with acutely exacerbated SCZ. Although the Ser carriers have numerically larger score reductions when compared with non-carriers in almost all PANSS dimensions, the difference of their effects is statistically not significant; however, the clinical factors, including dosage of aripiprazole, age, duration of illness, and diagnostic subtype could influence PANSS performance after aripiprazole treatment, suggesting that *DRD3* Ser9Gly polymorphism may not contribute significantly to inter-individual differences in therapeutic efficacy of aripiprazole, but some clinical factors may predict treatment efficacy [160].

The effects of aripiprazole and haloperidol have been studied in SH-SY5Y human neuroblastoma cells via BDNF-mediated signaling, glycogen synthase kinase-3beta (GSK-3beta), and B cell lymphoma protein-2 (Bcl-2). The effects of both drugs on *BDNF* gene promoter activity were studied in SH-SY5Y cells transfected with a rat *BDNF* promoter fragment (-108 to +340) linked to the luciferase reporter gene. Haloperidol was not associated with a significant difference in *BDNF* promoter activity. In contrast, aripiprazole was associated with increased *BDNF* promoter activity only with a dose of 10 μM (93%). Treatment with aripiprazole at 10 μM increased the levels of BDNF by 85%, compared with control levels, whereas haloperidol had no effect. Cells treated with aripirazole effectively increased the levels of GSK-3beta phosphorylation and Bcl-2 at doses of five and 10 μM (30% and 58% and 31% and 80%, respectively); however, haloperidol had no effects on p-GSK-3beta and Bcl-2 expression. This study seems to indicate that aripiprazole, but not haloperidol, may exert neuroprotective effects on human neuronal cells. The actions of signaling systems associated with BDNF may represent key targets for both aripiprazole and haloperidol, but the differential effects of both drugs suggest that the haloperidol-mediated responses may depend on different pharmacogenomic pathways [162].

The prototypical atypical antipsychotic agent, clozapine, is more efficacious for refractory SCZ than the ‘typical’ antipsychotics. Since 2002, at least 22 association studies have shown that *DTNBP1* can be associated with the risk for SCZ, and it has also been hypothesized that *DTNBP1* might influence the response to antipsychotic treatments. Patients with diplotype ACCCTC/GTTGCC, genotypes T/T + T/C, or allele T of marker rs742105 (P1333) have better response to clozapine, and patients with diplotype ACCCTC/GCCGCC, genotype A/G, or allele A of marker rs909706 (P1583) have better response to haloperidol in European-Americans, African-Americans, and/or the combined sample. European-American patients with diplotype ACCCTC/GCCGCC have worse response to clozapine on positive symptoms. These results obtained by Zuo *et al.* [163] might indicate that *DTNBP1* gene modulates the effects of both the atypical antipsychotic clozapine and the typical antipsychotic haloperidol and that SCZ patients with different *DTNBP1* diplotypes, haplotypes, genotypes, or alleles might have different responses to these antipsychotics [163]. 

Disruption of the Reelin and GABAergic signaling systems have been observed in psychiatric disorders including autism, SCZ, bipolar disorders (BD), and major depression. Chronic administration of psychotropic medications (clozapine, fluoxetine, haloperidol, lithium, olanzapine, and valproic acid) used in the treatment of psychiatric disorders alters levels of Reelin, its receptor Vldlr, downstream molecules Gsk3beta, Dab-1, and Gad65/67 in rat prefrontal cortex as measured by qRT-PCR and SDS-PAGE and western blotting. qRT-PCR revealed that mRNAs for Reelin, Vldlr, Dab-1, Gsk3beta, and Gad65 were each significantly altered by at least one of the drugs tested, and in the case of Reelin, Dab-1, and Gsk3beta, by multiple drugs, suggest that the Reelin signaling and GABAergic systems are affected by commonly used psychotropic medications [164]. Valproic acid facilitates chromatin remodeling when it is associated with clozapine or sulpiride but not with haloperidol or olanzapine. This remodeling might contribute to Reelin- and *GAD(67)*-promoter demethylation and might reverse the GABAergic-gene-expression downregulation associated with SCZ morbidity [165].

Flavin-containing monooxygenase 3 (*FMO3*) genotype data for European-, Latin-, African- and Asian-American SCZ patients administered olanzapine were compared to age-, gender-, and race/ethnicity-matched controls. SNPs and haplotypes associated with case-control status was undertaken to determine the potential role of *FMO3* in olanzapine therapeutic response. For European Americans, significant differences in individual cases *vs.* controls were observed between *FMO3* 158 and 257 alleles and genotype frequencies and SCZ delusions, hallucinations, and weight gain/increased appetite, but this was not observed in a replicated population. For Latin Americans, a significant difference in individual cases *vs.* controls was observed for *FMO3* 158 and 257 for SCZ delusions as well as hallucinations and delusions. Sleepiness and weight gain were associated with allele 308. In African-Americans, a comparison of allele frequency and diagnosis showed a significant dependence on allele 158 in individual cases *vs.* controls. *FMO3* genotype and allele frequency was not significantly associated with auditory hallucinations or delusions. For Asian-Americans, no significant difference in allele or genotype frequency and auditory hallucination and delusions was observed in individual cases *vs.* controls. In female Asian-Americans, allele frequency for *FMO3* 257 was significantly associated with diagnosis and in males, genotype frequency for *FMO3* 257 and diagnosis was significantly associated [166]. 

Single-locus as well as detailed haplotype-based association analysis of the *COMT* gene with SCZ and antipsychotic treatment response was carried out using seven *COMT* polymorphisms in schizophrenic patients from a homogeneous south Indian population. Haplotype analysis showed highly significant association of seven *COMT* marker haplotypes with SCZ, and allelic associations of two SNPs (rs4633, rs4680) with drug response were also found. A significant association of markers located between intron 1 and intron 2 (rs737865, rs6269), and in exon 4 (rs4818, rs4680) with drug response was also detected, indicating that the interacting effects within the *COMT* gene polymorphisms may influence the disease status and response to risperidone in SCZ patients [167]. 

Olanzapine is a second-generation antipychotic that may cause weight gain and metabolic syndrome in some cases. The peroxisome proliferator-activated receptor (*PPARG*) is an important gene in the progress of type II diabetes and metabolic syndrome. Significant differences were found between pre-treatment and post-treatment body mass index and weight change in Pro12Ala polymorphism of *PPARG2* in Turkish patients [168]. 

Twenty-one loci of diverse candidate genes encoding dopamine, serotonin (5-HT), histamine, and adrenergic receptors, tumor necrosis factor-alpha, ghrelin, adiponectin, and peroxisome proliferator-activated receptor gamma-2, were analyzed as candidate genes for olanzapine-related body weight gain. Olanzapine-induced weight gain correlated negatively with baseline BMI and positively with clinical global improvement and the length of olanzapine treatment, but it did not correlate with the daily dose of olanzapine, concomitant antipsychotics, sex, age, or smoking. Four genetic variants, the 102T allele of *HTR2A*, the 825T allele of *GNB3*, the 23Cys allele of *HTR2C*, and the 64Arg/Arg genotype of *ADRB3*, were significantly associated with olanzapine-induced weight gain. Stepwise regression analysis revealed that the baseline BMI predicted 12.5% of the weight gain, and the 2 latter genetic factors added 6.8%. The patients with double and triple genetic risk factors showed 5.1% and 8.8% BMI increases, respectively, during olanzapine treatment, whereas the patients with a single or no risk factor showed approximately a 1% BMI increase [169]. 

Genetic predisposition to clozapine-induced weight gain has also been suggested. 10 genetic polymorphisms across 9 candidate genes, including the serotonin 2C, 2A, and 1A receptor genes (*HTR2C/2A/1A*); the histamine H_1_ and H_2_ receptor genes (*H1R/H2R*); the cytochrome P450 1A2 gene (*CYPIA2*); the β_3_ and α-adrenergic receptor genes (*ADRB3/ADRAIA*); and tumor necrosis factor alpha (*TNFA*) have been studied. Trends were observed for *ADRB3*, *ADRA1A*, *TNFA*, and *HTR2C* [170].

Serotonin 2C and 2A receptor (5-HT2C and 5-HT2A) antagonisms are hypothesized to play a role in the metabolic adverse effects induced by olanzapine and clozapine. Associations have been reported between polymorphisms in 5-HT2C and 5-HT2A receptor coding genes, *HTR2C* and *HTR2A*, with antipsychotic-induced weight gain. Olanzapine-treated patients with *HTR2C* haplotype C (-759C, -697C, and 23Ser) had higher BMI and C peptide levels compared with patients with haplotype B (-759T, -697C, and 23Cys). The frequency of patients homozygous for the *HTR2C* haplotype A (-759C, -697G, and 23Cys) was significantly higher among clozapine-treated patients with obesity (BMI > 30 kg/m^2^) compared with nonobese patients. Patients carrying the *HTR2A* haplotype 2 (-1438A, 102T, and 452His) had significantly higher C peptide levels compared with haplotype 3 (-1438A, 102T, and 452Tyr) carriers in the olanzapine group and in the overall study population. None of the haplotypes were associated with serum levels of insulin, triglycerides, and cholesterol or with homeostasis model assessment index for insulin resistance. Both *HTR2C* and *HTR2A* gene polymorphisms seem to be associated with the occurrence of metabolic abnormalities in patients treated with olanzapine or clozapine [171]. 

The *LEPR* Q223R polymorphism (rs1137101) and the *LEP* promoter 2548G/A polymorphism (rs7799039) were postulated as candidate genes to be associated with obesity in patients using atypical antipsychotic drugs. In females, the *LEPR* 223QR and *LEPR* 223RR genotypes were associated with a lower risk of obesity. In males, this association was not found. In females, the average body weight was 13.6 kg more in the *LEPR* 223QQ group compared with the *LEPR* 223RR group. No significant association was found between the *LEP* promoter 2548G/A polymorphism and obesity. *LEPR* Q223R polymorphism may be associated with obesity in women with a psychotic disorder treated with atypical antipsychotic drugs [172]. 

An association has been reported between 5-hydroxytryptamine receptor 2C (*HTR2C*) polymorphisms and the occurrence of the metabolic syndrome in patients using antipsychotics. Primary determinants were polymorphisms in the promoter region of the *HTR2C* gene [*HTR2C*:c.1-142948(GT)_n_, rs3813929 (-759 C/T), and rs518147 (-697 G/C)] and an intragenic polymorphism (rs1414334:C > G). The variants of *HTR2C*:c.1-142948(GT)_n_ and rs1414334 were not significantly associated with the metabolic syndrome in the replication sample but did show significance in the pooled analysis. The variant rs1414334 C allele was specifically associated with the metabolic syndrome in patients using clozapine or risperidone. The increased risk for the metabolic syndrome is particularly strong in carriers of the rs1414334 C allele using clozapine or risperidone [173]. 

Sequence variations in the glutamate transporter gene *SLC1A1* (A/C/G haplotype at rs2228622-rs3780413-rs3780412) have been associated with susceptibility to atypical antipsychotic-induced obsessive-compulsive symptoms [174]. 

Several polymorphisms previously associated with the efficacy of the novel antipsychotic iloperidone could be used together to predict clinical response and provide practical information for individualized treatment. A recent study using 6 genetic markers of iloperidone response as measured by change in the Positive and Negative Syndrome Scale-Total (PANSS-T) score demonstrated that the 6-marker genotype combinations defined 4 groups of patients with distinct probabilities of response. Over 75% of iloperidone-treated patients in the group with the optimal genotype combinations showed a 20% or greater improvement, compared with 37% for patients with other genotypes. These patients had a significant response by the first week of treatment, which was earlier than for patients with other genotype combinations. These results illustrate the combined use of genetic markers to predict enhanced response to iloperidone and support the application of pharmacogenetics to differentiate medication options and improve individualized treatments for SCZ [175]. 

Genome-wide expression profiling to study effects of typical antipsychotics and atypical antipsychotics in the post-mortem liver of SCZ patients using microarrays revealed that typical antipsychotics affected genes associated with nuclear protein, stress responses and phosphorylation, whereas atypical antipsychotics affected genes associated with Golgi/endoplasmic reticulum and cytoplasm transport. Comparison between typical antipsychotics and atypical antipsychotics further identified genes associated with lipid metabolism and mitochondrial function. Analyses on individual antipsychotics identified a set of genes (151 transcripts) that are differentially regulated by four antipsychotics, particularly by phenothiazines, in the liver of SCZ patients [176].

Growing genetic evidence has implicated a role for neuregulin-1 (NRG-1) in SCZ pathogenesis as well as alterations in SNAP receptor (SNARE) proteins at both gene and protein levels in post-mortem investigations. Clozapine has been shown to increase both NRG-1 levels and synaptic markers in rodents. Clozapine has the ability to upregulate NRG-1 (+3.58 fold change) and VAMP-1 (+1.92) while SNAP-25 remaines unchanged [177]. 

An increasing number of experiments have found anomalies in mitochondria in the brains of psychotics, which suggests that mitochondrial dysfunction or abnormal cerebral energy metabolism might play an important role in the pathophysiology of SCZ. Differential mitochondrial protein expressions were assessed using two-dimensional (2D) gel electrophoresis for three groups with chlorpromazine (CPZ), clozapine (CLZ), quetiapine (QTP) and a control group. A total of 14 proteins, of which 6 belong to the respiratory electron transport chain (ETC) of oxidative phosphorylation (OXPHOS), showed significant changes in quantity including NADH dehydrogenase (ubiquinone) 1 alpha subcomplex 10 (Ndufa10), NADH dehydrogenase (ubiquinone) flavoprotein 2 (Ndufv2), NADH dehydrogenase (ubiquinone) Fe-S protein 3 (Ndufs3), F1-ATPase beta subunit (Atp5b), ATPase, H^+^ transporting, lysosomal, beta 56/58 kDa, isoform 2 (Atp6v1b2) and ATPase, H^+^ transporting, V1 subunit A, isoform 1 (Atp6v1a1). These data show proteomic changes induced by neuroleptics in rodents [178]. Ma *et al.* [179] used label-free liquid chromatography tandem mass spectrometry (LC-MSE) to identify differentially expressed proteins in rat frontal cortex following subchronic treatment with haloperidol or olanzapine. LC-MSE profiling identified 531 and 741 annotated proteins in fractions I (cytoplasmic-) and II (membrane enriched-) in the two drug treatments. Fifty-nine of these proteins were altered significantly by haloperidol treatment, 74 by olanzapine and 21 were common to both treatments. Pathway analysis revealed that both drugs altered similar classes of proteins associated with cellular assembly/organization, nervous system development/function (particularly presynaptic function) and neurological disorders, which indicate a common mechanism of action. The top affected canonical signaling pathways differed between the two treatments. The haloperidol data set showed a stronger association with Huntington’s disease signaling, while olanzapine treatment showed stronger effects on glycolysis/gluconeogenesis [179].

Selective serotonin reuptake inhibitor (SSRI) and antipsychotic co-administration is a widely used strategy to treat both psychotic depression and depressive symptoms in SCZ. It has been suggested that co-administration of SSRIs and antipsychotics may result in molecular changes different from their individual effects. Studies have been carried out on the acute effects of two SSRIs, citalopram and escitalopram, alone or in combination with haloperidol, on the expression of *Homer1a* together with its splice variant *Ania-3*, and *p11*, two genes linked respectively to dopaminergic and serotonergic neurotransmission and involved in synaptic plasticity. *Homer1a* and *Ania-3* were induced in the striatum by haloperidol, alone and in combination with SSRIs, but not by SSRIs alone. Haloperidol + citalopram co-administration induced a stronger *Homer1a* expression than haloperidol alone in the ventrolateral caudate-putamen. *Homer1a* was significantly down-regulated in the parietal cortex by all treatments. These results show that haloperidol + citalopram combination exerts synergistic effects on *Homer* expression, suggesting that citalopram may influence the impact of haloperidol on dopaminergic neurotransmission. *Homer1a* and *Ania-3* are strongly induced in striatum by haloperidol, while they are not influenced by citalopram or escitalopram in this region. In the cortex the two transcripts are modulated by both haloperidol and SSRIs, suggesting a possible role of both dopamine and serotonin in their cortical regulation [180].

## 6. CYPs in Dementia

In dementia, as in any other CNS disorder, CYP genomics is a very important issue since in practice over 90% of patients with dementia are daily consumers of psychotropics. Furthermore, some acetylcholinesterase inhibitors (the most prescribed anti-dementia drugs worldwide) are metabolized via CYP enzymes [6,7,102]. CYP2D6, CYP2C19, CYP2C9 and CYP3A4/5 deserve special consideration.

The CYP2D6 enzyme, encoded by a gene that maps on 22q13.1-13.2, catalyzes the oxidative metabolism of over 100 clinically important and commonly prescribed drugs such as cholinesterase inhibitors, antidepressants, neuroleptics, opioids, some β-blockers, class I antiarrhythmics, analgesics and many other drug categories [181], acting as substrates, inhibitors or inducers with which many other drugs may potentially interact, this leading to the outcome of ADRs. The *CYP2D6* locus is highly polymorphic, with over 100 different *CYP2D6* alleles identified in the general population showing deficient (PM), normal (EM), intermediate (IM) or increased enzymatic activity (UM) [182,183]. Most individuals (>80%) are EMs; however, remarkable interethnic differences exist in the frequency of the PM and UM phenotypes among different societies all over the world [16,141,184]. On average, approximately 6.28% of the world population belongs to the PM category. Europeans (7.86%), Polynesians (7.27%), and Africans (6.73%) exhibit the highest rate of PMs, whereas Orientals (0.94%) show the lowest rate. The frequency of PMs among Middle Eastern populations, Asians, and Americans is in the range of 2-3%. *CYP2D6* gene duplications are relatively infrequent among Northern Europeans, but in East Africa the frequency of alleles with duplication of *CYP2D6* is as high as 29% [185]. 

The most frequent *CYP2D6* alleles in the European population are as follows: CYP2D6*1 (wild-type)(normal), CYP2D6*2 (2850C > T) (normal), CYP2D6*3 (2549A > del) (inactive), CYP2D6*4 (1846G > A) (inactive), CYP2D6*5 (gene deletion) (inactive), CYP2D6*6 (1707T > del) (inactive), CYP2D6*7 (2935A > C) (inactive), CYP2D6*8 (1758G > T) (inactive),  CYP2D6*9 (2613-2615 delAGA) (partially active), CYP2D6*10 (100C > T) (partially active), CYP2D6*11 (883G > C) (inactive), CYP2D6*12 (124G > A) (inactive), CYP2D6*17 (1023C > T) (partially active), and *CYP2D6* gene duplications (with increased or decreased enzymatic activity depending upon the alleles involved) [6,8,11,16].

In the Spanish population, where the mixture of ancestral cultures has occurred for centuries, the distribution of the *CYP2D6* genotypes differentiates 4 major categories of *CYP2D6*-related metabolizer types: (i) Extensive Metabolizers (EM) (*1/*1, *1/*2,*1/*10); (ii) Intermediate Metabolizers (IM) (*1/*3, *1/*4, *1/*5, *1/*6, *1/*7, *10/*10, *4/*10, *6/*10, *7/*10); (iii) Poor Metabolizers (PM) (*4/*4, *5/*5); and (iv) Ultra-rapid Metabolizers (UM) (*1xN/*1, *1xN/*4, Dupl). In this sample we found 51.61% EMs, 32.26% IMs, 9.03% PMs, and 7.10% UMs [8,11,25,99,100,101,102,104]. In a more recent study with 1,637 subjects and 644 patients with AD we did not find any significant difference between AD cases and the general population (GP) [7]. A variation rate higher than 2% was only found in the EM-*1/*1 genotype which is more frequent in the GP than in AD. The proportion of EMs was 59.51% in GP and 57.76% in AD; IMs were 29% in GP and 31% in AD; PMs were 4.46% in GP and 5.27% in AD; and UMs were 6.23% in GP and 5.9% in AD [7]. No major differences between females and males were found in the GP group; however, in AD, EMs are more frequent in females than in males, and PMs are more frequent in males than in females, indicating that males might be at higher risk for developing ADRs [7].

### 6.1. Association of CYP2D6 Variants with Alzheimer’s Disease-Related Genes

We have also investigated the association of *CYP2D6* genotypes with AD-related genes, such as *APP*, *MAPT*, *APOE*, *PSEN1*, *PSEN2*, *A2M*, *ACE*, *AGT*, *FOS*, and *PRNP* variants [7,11,99,100,101,102,103,104]. Homozygous *APOE-2/2* (12.56%) and *APOE-4/4* (12.50%) accumulate in UMs, and APOE-4/4 cases were also more frequent in PMs (6.66%) than in EMs (3.95%) or IMs (0%). *PSEN1-1/1* genotypes were more frequent in EMs (45%), whereas *PSEN-1/2* genotypes were over-represented in IMs (63.16%) and UMs (60%). The presence of the *PSEN1-2/2* genotype was especially high in PMs (38.46%) and UMs (20%). A mutation in the *PSEN2* gene exon 5 (*PS2*E5+) was markedly present in UMs (66.67%). About 100% of UMs were *A2M*-V100I-A/A, and the *A2M*-V100I-G/G genotype was absent in PMs and UMs. The *A2M*-I/I genotype was absent in UMs, and 100% of UMs were *A2M*-I/D and *ACE*-D/D. Homozygous mutations in the *FOS* gene (B/B) were also only present in UMs. *AGT*-T235T cases were absent in PMs, and the *AGT*-M174M genotype appeared in 100% of PMs. Likewise, the *PRNP*-M129M variant was present in 100% of PMs and UMs. These association studies clearly show that in PMs and UMs there is an accumulation of AD-related polymorphic variants of risk which might be responsible for the defective therapeutic responses currently seen in these AD clusters [11,99,100,101,102].

### 6.2. CYP2D6-Related Biochemical and Hemodynamic Phenotypes in Alzheimer’s Disease

It appears that different *CYP2D6* variants, expressing EMs, IMs, PMs, and UMs, influence to some extent several biochemical parameters, liver function, and vascular hemodynamic parameters which might affect drug efficacy and safety. Blood glucose levels are found to be elevated in EMs (*1/*1 vs. *4/*10) and in some IMs (*4/*10 vs. *1xN/*4), whereas other IMs (*1/*5 vs. *4/*4) tend to show lower levels of glucose compared with PMs (*4/*4) or UMs (*1xN/*4). The highest levels of total-cholesterol are detected in the EMs with the CYP2D6*1/*10 genotype (vs. *1/*1, *1/*4 and *1xN/*1). The same pattern has been observed with regard to LDL-cholesterol levels, which are significantly higher in the EM-*1/*10. In general, both total cholesterol levels and LDL-cholesterol levels are higher in EMs (with a significant difference between *1/*1 and *1/*10), intermediate levels are seen in IMs, and much lower levels in PMs and UMs; and the opposite occurs with HDL-cholesterol levels, which on average appear much lower in EMs than in IMs, PMs, and UMs, with the highest levels detected in *1/*3 and *1xN/*4. The levels of triglycerides are highly variable among different *CYP2D6* polymorphisms, with the highest levels present in IMs (*4/*10 vs. *4/*5 and *1xN/*1) [11,102,104]. These data clearly indicate that lipid metabolism can be influenced by *CYP2D6* variants or that specific phenotypes determined by multiple lipid-related genomic clusters are necessary to confer the character of EMs and IMs. Another possibility might be that some lipid metabolism genotypes interact with *CYP2D6*-related enzyme products leading to the definition of the pheno-genotype of PMs and UMs. No significant changes in blood pressure values have been found among *CYP2D6* genotypes; however, important differences became apparent in brain cerebrovascular hemodynamics. In general terms, the best cerebrovascular hemodynamic pattern is observed in EMs and PMs, with higher brain blood flow velocities and lower resistance and pulsatility indices, but differential phenotypic profiles are detectable among *CYP2D6* genotypes. For instance, systolic blood flow velocities (Sv) in the left middle cerebral arteries (LMCA) of AD patients are significantly lower in *1/*10 EMs, with high total cholesterol and LDL-cholesterol levels, than in IMs (*4/*10); and diastolic velocities (Dv) also tend to be much lower in *1/*10 and especially in PMs (*4/*4) and UMs (*1xN/*4), whereas the best Dv is measured in *1/*5 IMs. More striking are the results of both the pulsatility index (PI = (Sv-Dv)/Mv) and resistance index (RI = (Sv-Dv)/Sv), which are worse in IMs and PMs than in EMs and UMs. These data taken together seem to indicate that *CYP2D6*-related AD PMs exhibit a poorer cerebrovascular function which might affect drug penetration into the brain with the consequent therapeutic implications [11,99,100,101,102,103,104].

### 6.3. Influence of CYP2D6 Genotypes on Liver Transaminase Activity

In order to elucidate whether or not *CYP2D6*-related variants may influence transaminase activity, we have studied the association of GOT, GPT, and GGT activity with the most prevalent *CYP2D6* genotypes in AD [11,100,101,102]. Globally, UMs and PMs tend to show the highest GOT activity and IMs the lowest. Significant differences appear among different IM-related genotypes. The *10/*10 genotype exhibited the lowest GOT activity with marked differences as compared to UMs. GPT activity was significantly higher in PMs (*4/*4) than in EMs (*1/*10) or IMs (*1/*4, *1/*5). The lowest GPT activity was found in EMs and IMs. Striking differences have been found in GGT activity between PMs (*4/*4), which showed the highest levels, and EMs (*1/*1; *1/*10), IMs (*1/*5), or UMs (*1xN/*1) [102]. Interestingly enough, the *10/*10 genotype, with the lowest values of GOT and GPT, exhibited the second highest levels of GGT after *4/*4, probably indicating that *CYP2D6*-related enzymes differentially regulate drug metabolism and transaminase activity in the liver. These results are also clear in demonstrating the direct effect of *CYP2D6* variants on transaminase activity [11,101,102].

## 7. CYP2D6-Related Therapeutic Response to a Multifactorial Treatment in Dementia

Few prospective clinical trials have been performed to elucidate the influence of *CYP2D6* variants on the therapeutic outcome in AD in response to cholinesterase inhibitors or other anti-dementia drugs. We have performed the first prospective study in AD patients who received a combination therapy with (i) an endogenous nucleotide and choline donor, CDP-choline (500 mg/day); (ii) a nootropic substance, piracetam (1600 mg/day); (iii) a vasoactive compound, 1,6 dimethyl 8β-(5-bromonicotinoyl-oxymethyl)-10α-methoxyergoline (nicergoline) (5 mg/day); and (iv) a cholinesterase inhibitor, donepezil (5 mg/day), for one year. With this multifactorial therapeutic intervention, EMs improved their cognitive function (MMSE score) from 21.58 ± 9.02 at baseline to 23.78 ± 5.81 after 1 year of treatment. IMs also improved from 21.40 ± 6.28 to 22.50 ± 5.07 (r = + 0.96), whereas PMs and UMs deteriorate from 20.74 ± 6.72 to 18.07 ± 5.52 (r = -0.97), and from 22.65 ± 6.76 to 21.28 ± 7.75 (r = -0.92), respectively. According to these results, PMs and UMs were the worst responders, showing a progressive cognitive decline with no therapeutic effect, and EMs and IMs were the best responders, with a clear improvement in cognition after one year of treatment. Among EMs, AD patients harboring the *1/*10 genotype responded better than patients with the *1/*1 genotype. The best responders among IMs were the *1/*3, *1/*6 and *1/*5 genotypes, whereas the *1/*4, *10/*10, and *4/*10 genotypes were poor responders. Among PMs and UMs, the poorest responders were carriers of the  *4/*4 and  *1xN/*1 genotypes, respectively [7,8,11,25,99,100,101,102]. In a recent study, Pilotto *et al.* [186] have confirmed the influence of *CYP2D6* variants (rs1080985) on the efficacy of donepezil in AD. 

From all these data we can conclude the following: (i) The most frequent *CYP2D6* variants in the Southern European population (Iberian peninsula) are the *1/*1 (57.84%), *1/*4 (22.78%), *1xN/*1 (6.10%), *4/*4 (2.56%), and *1/*3 (2.01%) genotypes, accounting for more than 80% of the population; (ii) the frequency of EMs, IMs, PMs, and UMs is about 59.51%, 29,78%, 4.46%, and 6.23%, respectively, in the general population, and 57.76, 31.05%, 5.27%, and 5.90%, respectively in AD cases; (iii) EMs are more prevalent in GP (59.51%) than in AD (57.76%); IMs are more frequent in AD (31.05%) than in GP (29.78%); the frequency of PMs is slightly higher in AD (5.27%%) than in GP (4.46%); and UMs are more frequent in GP (6.23%) than in AD (5.90%); (iv) there are differences between females and males in the distribution and frequency of *CYP2D6* genotypes which might be of relevance in therapeutic terms and risk of ADRs; (v) there is an accumulation of AD-related genes of risk in PMs and UMs; (vi) PMs and UMs tend to show higher transaminase activities than EMs and IMs; (vii) EMs and IMs are the best responders, and PMs and UMs are the worst responders to a combination therapy with cholinesterase inhibitors, neuroprotectants, and vasoactive substances; and (viii) the pharmacogenetic response in AD appears to be dependent upon the networking activity of genes involved in drug metabolism and genes involved in AD pathogenesis [6,7,8,11,25,99,100,101,102].

### 7.1. CYP Clustering in Alzheimer’s Disease

Since over half of the available drugs are metabolized via different CYP enzymes and other metabolic pathways, it is convenient to understand the networking activity of CYP genes and the genomic profiles of these genes in particular groups of risk. In the case of dementia, 73.71% of AD patients are *CYP2C19*-EMs, 25.12% IMs, and 1.16% PMs. The distribution and frequency of *CYP2C9* genotypes is as follows: *1/*1-EM 60.87%, *1/*2-IM 23.98%, *1/*3-IM 10.17%, *2/*2-PM 2.54%, *2/*3-PM 2.16%, and *3/*3-PM 0.25%, globally representing 60.87% *CYP2C9*-EMs, 34.16% IMs, and 4.97% PMs [7]. This is especially important because the *CYP2C9*-Ile359Leu (CYP2C9*3 allele) and *CYP2C9*-Arg144Cys (CYP2C9*2 allele) variants are associated with warfarin sensitivity. Clustering together *CYP2C9* and *VKORC1* variants, we can estimate that approximately 30% of the elderly population is sensitive to warfarin anticoagulants. 

Concerning *CYP3A4*/5 polymorphisms, 82.75% of AD cases are EMs (CYP3A5*3/*3), 15.88% are IMs (CYP3A5*1/*3), and 1.37% are UMs (CYP3A5*1/*1) [7]. The human CYP3A subfamily plays a dominant role in the metabolic elimination of more drugs than any other biotransformation enzyme. CYP3A enzyme is localized in the liver and small intestine and thus contributes to first-pass and systemic metabolism. CYP3A expression varies as much as 40-fold in liver and small intestine donor tissues. Unlike other human P450s (*CYP2D6*, *CYP2C19*) there is no evidence of a ‘null’ allele for *CYP3A4*. Over 50 SNPs have been identified in the *CYP3A4* gene. The most common variant, CYP3A4*1B, is an A-392G transition in the 5'-flanking region with an allele frequency ranging from 0% (Chinese and Japanese) to 45% (African-Americans). *CYP3A5* is polymorphically expressed in adults with readily detectable expression in about 10-20% in Caucasians, 33% in Japanese and 55% in African-Americans. The primary causal mutation for its polymorphic expression (CYP3A5*3) confers low CYP3A5 protein expression as a result of improper mRNA splicing and reduced translation of a functional protein. The CYP3A5*3 allele frequency varies from approximately 50% in African-Americans to 90% in Caucasians. Functionally, microsomes from a CYP3A5*3/*3 liver contain very low CYP3A5 protein and display on average reduced catalytic activity towards midazolam. Additional intronic or exonic mutations (CYP3A5*5, *6, and *7) may alter splicing and result in premature stop codons or exon deletion. As *CYP3A5* is the primary extrahepatic CYP3A isoform, its polymorphic expression may be implicated in disease risk and the metabolism of endogenous steroids or xenobiotics [187]. 

The construction of a genetic map integrating the most prevalent *CYP2D6* + *CYP2C19* + *CYP2C9* polymorphic variants in a trigenic cluster yields 82 different haplotype-like profiles. The most frequent trigenic genotypes in the AD population are *1*1-*1*1-*1*1 (25.70%), *1*1-*1*2-*1*2 (10.66%), *1*1-*1*1-*1*1 (10.45%), *1*4-*1*1-*1*1 (8.09%), *1*4-*1*2-*1*1 (4.91%), *1*4-*1*1-*1*2 (4.65%), and *1*1-*1*3-*1*3 (4.33%). These 82 trigenic genotypes represent 36 different pharmacogenetic phenotypes. According to these trigenic clusters, only 26.51% of the patients show a pure 3EM phenotype, 15.29% are 2EM1IM, 2.04% are pure 3IM, 0% are pure 3PM, and 0% are 1UM2PM (the worst possible phenotype) [7].

Taking into consideration the data available, it might be inferred that at least 10-15% of the AD population may exhibit an abnormal metabolism of cholinesterase inhibitors and/or other drugs which undergo oxidation via CYP2D6-related enzymes. Approximately 50% of this population cluster would show an ultrarapid metabolism, requiring higher doses of cholinesterase inhibitors in order to reach a therapeutic threshold, whereas the other 50% of the cluster would exhibit a poor metabolism, displaying potential adverse events at low doses. If we take into account that approximately 60–70% of therapeutic outcomes depend upon pharmacogenomic criteria (e.g., pathogenic mechanisms associated with AD-related genes), it can be postulated that pharmacogenetic and pharmacogenomic factors are responsible for 75–85% of the therapeutic response (efficacy) in AD patients treated with conventional drugs [6,7,8,99,100,101]. Of particular interest are the potential interactions of cholinesterase inhibitors with other drugs of current use in patients with AD, such as antidepressants, neuroleptics, antiarrhythmics, analgesics, and antiemetics which are metabolized by the cytochrome P450 CYP2D6 enzyme. Although most studies predict the safety of donepezil and galantamine, as the two principal cholinesterase inhibitors metabolized by *CYP2D6*-related enzymes, few pharmacogenetic studies have been performed so far on an individual basis to personalize the treatment, and most studies reporting safety issues are the result of pooling together pharmacological and clinical information obtained with routine procedures. In certain cases, genetic polymorphism in the expression of *CYP2D6* is not expected to affect the pharmacodynamics of some cholinesterase inhibitors because major metabolic pathways are glucuronidation, *O*-demethylation, *N*-demethylation, *N*-oxidation, and epimerization. However, excretion rates are substantially different in EMs and PMs. For instance, in EMs, urinary metabolites resulting from *O*-demethylation of galantamine represent 33.2% of the dose compared with 5.2% in PMs, which show correspondingly higher urinary excretion of unchanged galantamine and its *N*-oxide [188]. Therefore, there are still many unanswered questions regarding the metabolism of cholinesterase inhibitors and their interaction with other drugs (potentially leading to ADRs) which require pharmacogenetic elucidation. It is also worth mentioning that dose titration (a common practice in AD patients treated with cholinesterase inhibitors, e.g., tacrine, donepezil) is an unwise strategy, since approximately 30-60% of drug failure or lack of therapeutic efficacy (and/or ADR manifestation) is not a matter of drug dosage but a problem of poor metabolizing capacity in PMs. Additionally, inappropriate drug use is one of the risk factors for adverse drug reactions (ADRs) in the elderly. The prevalence of use of potentially inappropriate medications in patients older than 65 years of age admitted to a general medical or geriatric ward ranges from 16% to 20% [189], and these numbers may double in ambulatory patients. Overall, the most prevalent inappropriate drugs currently prescribed to the elderly are amiodarone, long-acting benzodiazepines and anticholinergic antispasmodics; however, the list of drugs with potential risk also include antidepressants, antihistaminics, NSAIDs, amphetamines, laxatives, clonidine, indomethacin, and several neuroleptics [189], most of which are processed via CYP2D6 and CYP3A5 enzymes. Therefore, pre-treatment CYP screening might be of great help in order to rationalize and optimize therapeutics in the elderly, by avoiding medications of risk in PMs and UMs. 

## 8. Pharmacogenomics of AD-Related Genes

The pharmacogenomics of AD is still in a very primitive stage. In over 100 clinical trials for dementia, *APOE* has been used as the only gene of reference for the pharmacogenomics of AD [6,7,8,99,100,101,190,191]. Several studies indicate that the presence of the *APOE-4* allele differentially affects the quality and extent of drug responsiveness in AD patients treated with cholinergic enhancers (tacrine, donepezil, galantamine, rivastigmine), neuroprotective compounds (nootropics), endogenous nucleotides (CDP-choline), immunotrophins (anapsos), neurotrophic factors (cerebrolysin), rosiglitazone or combination therapies [6,7,8,99,100,101,102,190,191,192]; however, controversial results are frequently found due to methodological problems, study design, and patient recruitment in clinical trials. 

In long-term open clinical trials with a multifactorial treatment, *APOE-4/4* carriers are the worst responders [6,7,8,99,100,101,102]. With a similar therapeutic protocol, *PSEN1-1/1* homozygotes are the worst responders and *PSEN1-2/2* carriers are the best responders [7]. Significant *ACE*-related therapeutic responses to multifactorial treatments have also been reported [7,102]. Among *ACE*-I/D variants, *ACE*-D/D patients were the worst responders (r = -0.58), and *ACE*-I/D carriers were the best responders (r = + 0.26), with *ACE*-I/I showing an intermediate positive response (r = + 0.01) [7,102]. *ACE*-related biochemical and hemodynamic phenotypes have been studied in patients with AD [8,11,16]. *ACE*-I/I patients tend to be younger than *ACE*-I/D or *ACE*-D/D patients at the time of diagnosis and also to show a more severe cognitive deterioration. Serum ApoE, total cholesterol, LDL-cholesterol, HDL-cholesterol, nitric oxide, histamine, and ACE levels are higher in *ACE*-I/I carriers than in patients with the other genotypes; in contrast, serum triglyceride and VLDL levels are notably lower in *ACE*-I/I patients compared to patients harboring the *ACE*-I/D or *ACE*-D/D genotypes, whereas Aβ levels do not show any clear difference among *ACE*-related genotypes. Cerebrovascular function tends to be worse in *ACE*-D/D, with lower brain blood flow velocities and higher pulsatility and resistance indices, than in *ACE*-I/D (intermediate cerebrovascular hemodynamics) or *ACE*-I/I (almost normal cerebrovascular function) [8,11,16,102]. The correlation between lipid levels and brain hemodynamics is very similar in this study to data observed in that of *CYP2D6*-related metabolizer profiles in which EM patients with moderate cholesterol and lipoprotein levels (as well as relatively high nitric oxide, histamine, ACE, and ApoE levels) tend to show a better cerebrovascular hemodynamic profile than AD patients with lower cholesterol and lipoprotein levels [102]. This apparently paradoxical correlation appears to indicate that major influences in cerebrovascular homeostasis and hemodynamic brain blood flow are cholesterol, lipoproteins, nitric oxide, ACE, and histamine, among many other factors, in AD, and that peripheral levels of Aβ are indifferent in this concern. On the other hand, it seems likely that low triglyceride levels may facilitate cerebrovascular function. It is also worth mentioning that *ACE*-I/I patients with the highest cholesterol levels are the worst in mental performance. Other interpretations of these data might suggest an association of poor cerebrovascular function with *ACE*-D/D and *ACE*-I/D, and an association of alterations in lipid metabolism with *ACE*-I/I [11,102]. 

Both *APOE* and *ACE* variants also affect behavior and the modification of behavioral changes (mood, anxiety) in dementia after non-psychotropic pharmacological treatment [6,8,11,100,103]. At baseline, all *APOE* variants show similar anxiety and depression rates, except the *APOE-4/4* carriers who differed from the rest in significantly lower rates of anxiety and depression. Remarkable changes in anxiety were found among different *APOE* genotypes. Practically all *APOE* variants responded with a significant diminution of anxiogenic symptoms, except patients with the *APOE-4/4* genotype who only showed a slight improvement. The best responders were *APOE-2/4* ( r = -0.87) > *APOE-2/3* (r = -0.77) > *APOE-3/3* (r = -0.69) > *APOE-3/4* carriers (r = -0.45). The potential influence of *APOE* variants on anxiety and cognition in AD does not show a clear parallelism, suggesting that other more complex mechanisms are involved in the onset of anxiety in dementia. Concerning depression, all *APOE* genotypes improved their depressive symptoms with treatment except those with the *APOE-4/4* genotype, which worsen along the treatment period. The best responders were *APOE-2/4* (r = -0.85) > *APOE-2/3* (r = -0.77) > *APOE-3/3* (r = -0.73) > *APOE-3/4* (r = -0.16), and the worst responder was *APOE-4/4* (r = + 0.31) [11,102]. Patients with each one of the 3 *ACE*-I/D indel variants were equally anxiogenic and depressive at baseline and all of them responded favorably to the multifactorial protocol by gradually reducing anxiety and depressive symptoms over the 12-month treatment period. The best responders were *ACE*-I/D (r = -0.89) > *ACE*-D/D (r = -0.68) > *ACE*-I/I (r = -0.08). Depressive symptoms were also similarly improved in all *ACE*-I/D variants. The best responders were *ACE*-I/D (r = -0.88) > *ACE*-D/D (r = -0.55) > *ACE*-I/I (r = -0.13). Comparatively, the worst responders among *ACE*-I/D variants were carriers of the *ACE*-I/I genotype which were also the poorest responders in anxiety and cognition [11,102,104].

The combination of *APOE* and *ACE* polymorphic variants in bigenic clusters yielded different anxiety and depression patterns at baseline and after one year of treatment. The most anxiogenic patients at baseline were those with the 23DD, 44ID, and 34II genotypes, and the least anxiogenic patients were those harboring the 23II, 44DD, and 23ID genotypes. The most depressive clusters at baseline were those harboring the 23DD, 33ID, and 33II genotypes, with a clear accumulation of *APOE-3/3* carriers in these groups, and the least depressive clusters were those represented by carriers of the 23II, 44ID, and 23ID genotypes. All bigenic clusters showed a positive anxiolytic and anti-depressive response to the multifactorial treatment, except 44DD carriers who exhibited the worst response [11,102,104].

*APOE* influences liver function and *CYP2D6*-related enzyme activity probably via regulation of hepatic lipid metabolism. It has been observed that *APOE* may influence liver function and drug metabolism by modifying hepatic steatosis and transaminase activity. There is a clear correlation between *APOE*-related TG levels and GOT, GPT, and GGT activities in AD [11,102]. Both plasma TG levels and transaminase activity are significantly lower in AD patients harboring the *APOE-4/4* genotype, probably indicating (i) that low TG levels protect against liver steatosis; and (ii) that the presence of the *APOE-4* allele influences TG levels, liver steatosis, and transaminase activity. Consequently, it is very likely that *APOE* influences drug metabolism in the liver through different mechanisms, including interactions with enzymes such as transaminases and/or cytochrome P450-related enzymes encoded in genes of the CYP superfamily [11,101,104].

When *APOE* and *CYP2D6* genotypes are integrated in bigenic clusters and the *APOE* + *CYP2D6*-related therapeutic response to a combination therapy is analyzed in AD patients, it becomes clear that the presence of the *APOE-4/4* genotype is able to convert pure CYP2D6*1/*1 EMs into full PMs, indicating the existence of a powerful influence of the *APOE-4* homozygous genotype on the drug metabolizing capacity of pure *CYP2D6*-EMs. In addition, a clear accumulation of *APOE-4/4* genotypes is observed among *CYP2D6* PMs and UMs [7].

From these studies we can conclude the following: (i) Most studies with acetylcholinesterase inhibitors indicate that the presence or absence of the *APOE-4* allele influences the therapeutic outcome in patients with AD; (ii) Multifactorial treatments combining neuroprotectants, endogenous nucleotides, nootropic agents, vasoactive substances, cholinesterase inhibitors, and NMDA antagonists associated with metabolic supplementation on an individual basis adapted to the phenotype of the patient may be useful to improve cognition and to slow down disease progression in AD; (iii) The therapeutic response in AD seems to be genotype-specific under different pharmacogenomic conditions; (iv) In monogenic-related studies, patients harboring the *APOE-4/4* genotype are the worst responders; (v) *APP*, *PSEN1* and *PSEN2* mutations influence the therapeutic response in AD; (vi) In trigenic-related studies (*APOE* + *PSEN1* + *PSEN2*) the best responders are those patients carrying the 331222-, 341122-, 341222-, and 441112- genomic clusters; (vii) The worst responders in all genomic clusters are patients with the 441122+ genotype; (viii) The interaction of several AD-related genes seems to be determinant for drug efficacy and safety; (ix) *APOE-CYP2D6* interactions might influence the therapeutic response in AD via changes in lipid metabolism and liver function; (x) *APOE* may also interact with *PSEN1*, *ACE*, *A2M* and other genes to regulate the effect of drugs on cognition and behavioral changes in dementia; (xi) The *APOE-4/4* genotype seems to accelerate neurodegeneration anticipating the onset of the disease by 5-10 years; and, in general, *APOE-4/4* carriers show a faster disease progression and a poorer therapeutic response to all available treatments than any other polymorphic variant; (xii) Pharmacogenomic studies using monogenic, bigenic, trigenic, tetragenic or polygenic clusters as a harmonization procedure to reduce genomic heterogeneity in clinical trials are very useful in order to widen the therapeutic scope of limited pharmacological resources [6,7,8,9,10,11,12,13,14,15,16,99,100,101,102,103,104].

### 8.1. APOE-Related Therapeutic Response to a Multifactorial Therapy in Alzheimer’s Disease

**Figure 1:**
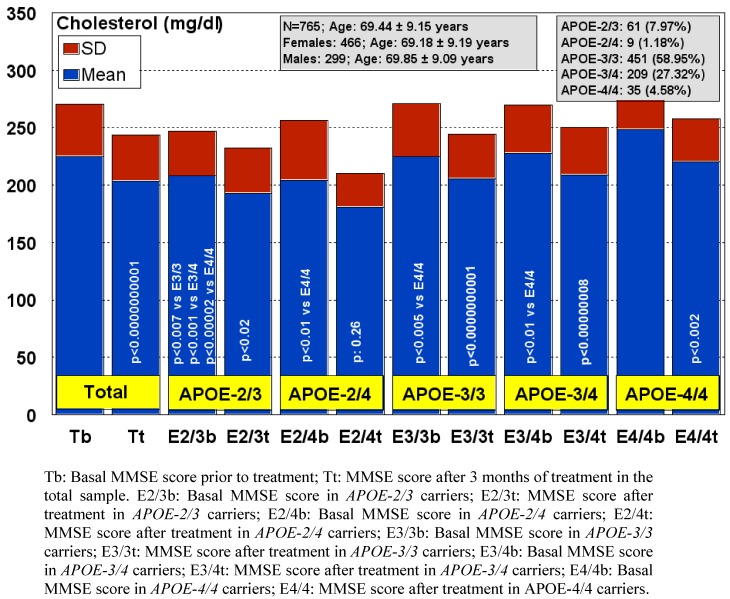
*APOE*-Related total cholesterol levels response to a multifactorial therapy in patients with dementia.

Patients with dementia (N=765, age: 69.44 ± 9.15 years, range: 50-96 years; 466 females, age: 69.18 ± 9.19 years, range: 50-96 years; and 299 males, age: 69.85 ± 9.09 years, range: 50-91 years; p < 0.01) received for three months a multifactorial therapy integrated by CDP-choline (500 mg/day, p.o.), Nicergoline (5 mg/day, p.o.), Sardilipin (E-SAR-94010)(LipoEsar^®^)(250 mg, t.i.d.), and Animon Complex^®^ (2 capsules/day), a nutraceutical compound integrated by a purified extract of *Chenopodium quinoa* (250 mg), ferrous sulphate (38.1 mg equivalent to 14 mg of iron), folic acid (200 µg), and vitamin B_12_ (1 µg) per capsule (RGS: 26.06671/C). Patients with chronic deficiency of iron (<35 µg/mL), folic acid (<2.5 ng/mL) or vitamin B_12_ (<150 pg/mL) received an additional supplementation of iron (80 mg/day), folic acid (5 mg/day) and B complex vitamins (B_1_, 15 mg/day; B_2_, 15 mg/day; B_6_, 10 mg/day; B_12_, 10 µg/day; nicotinamide, 50 mg/day), respectively, to maintain stable levels of serum iron (50–150 µg/mL), folic acid (5–20 ng/mL) and vitamin B_12_ levels (500–1000 pg/mL) in order to avoid the negative influence of all these metabolic factors on cognition [102,103]. Patients with hypertension (>150/85 mmHg) received Enalapril (20 mg/day). The frequency of *APOE* genotypes was: *APOE-2/3*, 7.97%; *APOE-2/4*, 1.18%; *APOE-3/3*, 58.95%; *APOE-3/4*, 27.32%; and *APOE-4/4*, 4.58% (Figure 1). Blood pressure, psychometric assessment (Mini-Mental State Examination, MMSE; ADAS; Hamilton Rating Scale-Depression, HAM-D; Hamilton Rating Scale-Anxiety, HAM-A), and blood parameters (glucose, total cholesterol, HDL-cholesterol, LDL-cholesterol, triglyceride, iron, folate, vitamin B_12_, TSH, T_4_) were evaluated at baseline and after 3 months of treatment.

Systolic (p < 0.0002) and diastolic blood pressure (p < 0.001), cognitive function (as assessed by MMSE, 20.51 ± 6.51 *vs.* 21.45 ± 6.95, p < 0.0000000001; ADAS-Cog, 22.94 ± 13.87 *vs.* 21.23 ± 12.84, p < 0.0001; ADAS-Non-Cog, 5.26 ± 4.18 *vs.* 4.15 ± 3.63, p < 0.0000000001; ADAS-Total, 27.12 ± 16.93 *vs.* 24.28 ± 15.06, p < 0.00009), and mood (HAM-A, 11.35 ± 5.44 *vs.* 9.79 ± 4.33, p < 0.0000000001; HAM-D, 10.14 ± 5.23 *vs.* 8.59 ± 4.30, p < 0.0000000001) improved after treatment. Glucose levels did not change. Total cholesterol levels (224.78 ± 45.53 *vs.* 203.64 ± 39.69 mg/dL, p < 0.0000000001), HDL-cholesterol levels (54.11 ± 14.54 *vs.* 52.54 ± 14.86 mg/dL, p < 0.0001), and LDL-cholesterol levels (148.15 ± 39.13 *vs.* 128.89 ± 34.83 mg/dL, p < 0.0000000001) were significantly reduced, whereas triglyceride levels increased (111.99 ± 67.14 *vs.* 120.69 ± 67.14 mg/dL, p < 0.0006) after 3 months of combined treatment. Folate (7.07 ± 3.61 *vs.* 18.14 ± 4.23 ng/mL, p < 0.000000001) and vitamin B_12_ levels (459.65 ± 205.80 *vs.* 689.78 ± 338.82 pg/mL, p < 0.000000001) also increased, and both TSH and T_4_ levels remained unchanged after treatment. The response rate in terms of cognitive improvement was as follows: 59.74% responders (RRs), 24.44% non-responders (NRs), and 15.82% stable responders (SRs)(no change in MMSE score after three months of treatment). The response rate in cholesterol levels was very similar: 57.78% RRs, 28.50% NRs, and 13.72% SRs. 

In this study, the basal MMSE score differed in *APOE-2/3* carriers with respect to *APOE-2/4* (p < 0.02), *APOE-3/4* (p < 0.004), and *APOE-4/4* carriers (p < 0.0009); in *APOE-3/3* *vs. APOE-3/4* (p < 0.0005), and *APOE-3/3* *vs. APOE-4/4* (p < 0.002). The best responders were *APOE-3/3* (p < 0.0000000001) > *APOE-3/4* (p < 0.00001) > *APOE-4/4* carriers (p < 0.05). Patients harboring the *APOE-2/3* and *APOE-2/4* genotypes did not show any significant improvement. The response rate by genotype was the following: *APOE-2/3*: 44.26% RRs, 36.07% NRs, 19.67% SRs; *APOE-2/4*: 55.56% RRs, 44.44% NRs, 0.0% SRs; *APOE-3/3*: 63.42% RRs, 21.06% NRs, 15.52% SRs; *APOE-3/4*: 56.94% RRs, 27.75% NRs, 15.31% SRs; *APOE-4/4*: 51.43% RRs, 28.57% NRs, 20.00% SRs. 

Systolic blood pressure (SBP) was significantly reduced in patients with the *APOE-3/3* (p < 0.00007) and *APOE-3/4* genotypes (p < 0.01), and diastolic blood pressure exhibited a similar pattern (*APOE-3/3*, p < 0.005; *APOE-3/4*, p < 0.01), with no changes in either SBP or DBP in *APOE-2/3*, *APOE-2/4* and *APOE-4/4* carriers.

Glucose levels tended to decrease in *APOE-4* allele carriers, but only patients with the *APOE-3/4* genotype showed a significant reduction in glucose levels (p < 0.02). In contrast, *APOE-2/3* carriers showed a tendency to increased glucose levels.

### 8.2. APOE-related blood lipid response to Sardilipin (E-SAR-94010)

Basal cholesterol levels were significantly different in patients with the *APOE-2/3* genotype *vs. APOE-3/3* (p < 0.007), *vs. APOE-3/4* (p < 0.001), *vs. APOE-4/4* (p < 0.00002); *APOE-2/4 vs. APOE-4/4* (p < 0.01); *APOE-3/3 vs. APOE-4/4* (p < 0.005); and *APOE-3/4 vs. APOE-4/4* (p < 0.01). 

The highest cholesterol levels were seen in *APOE-4/4* > *APOE-3/4* > *APOE-3/3.* All patients showed a clear reduction in cholesterol levels after treatment with Sardilipin. This was particularly significant in *APOE-3/3* (p < 0.0000000001) > *APOE-3/4* (p < 0.00000008) > *APOE-4/4* (p < 0.002) > *APOE-2/3* (p < 0.02) > *APOE-2/4* carriers (p: 0.26) (Figure 1). The response rate by genotype was as follows: *APOE-2/3*: 63.93% RRs, 29.51% NRs, 6.56% SRs; *APOE-2/4*: 44.44% RRs, 22.22% NRs, 33.34% SRs; *APOE-3/3*: 54.32% RRs, 28.16% NRs, 17.52% SRs; *APOE-3/4*: 53.59% RRs, 31.58% NRs, 14.83% SRs; *APOE-4/4*: 65.71% RRs, 20.00% NRs, 14.29% SRs. 

HDL-cholesterol levels significantly decreased in *APOE-3/3* (p < 0.001) > *APOE-3/4* (p < 0.05), with no significant changes in patients with other genotypes. In contrast, LDL-cholesterol levels showed identical changes to those observed in total cholesterol, with similar differences among genotypes at baseline and almost identical decreased levels after treatment (*APOE-3/3*, p > 0.0000000001; > *APOE-3/4*, p < 0.00001; > *APOE-2/3*, p < 0.0004; > *APOE-4/4*, p < 0.001; > *APOE-2/4*, p:0.31).

Paradoxically, triglyceride levels tended to increase in all *APOE* genotypes (*APOE-3/3*, p < 0.01; > *APOE-4/4*, p < 0.03; > *APOE-2/3*, p:0.12; > *APOE-3/4*, p:0.17), except in *APOE-2/4* carriers, who showed a tendency to decrease. Basal triglyceride levels were significantly lower in *APOE-4/4* carriers than in *APOE-2/3* (p < 0.03) and *APOE-3/4* carriers (p < 0.04).

Sardilipin (E-SAR-94010, LipoEsar^®^, LipoSea^®^) is a natural product extracted from the marine species *Sardina pilchardus*, by means of non-denaturing biotechnological procedures [193]. The main chemical compounds of LipoEsar^®^ are lipoproteins (60–80%) whose micelle structure probably mimics that of physiological lipoproteins involved in lipid metabolism. In preclinical studies, sardilipin has shown to be effective in (i) reducing blood cholesterol (CHO), triglyceride (TG), uric acid (UA), and glucose (Glu) levels, as well as liver alanine aminotransferase (ALT), and aspartate aminotransferase (AST) activity; (ii) enhancing immunological function by regulating both lymphocyte and microglia activity; (iii) inducing antioxidant effects mediated by superoxide dismutase activity; and (iv) improving cognitive function [16,193,194]. 

According to these results, it appears that the therapeutic response of patients with dyslipidemia to sardilipin is *APOE*-related. The best responders were patients with *APOE-3/3* > *APOE-3/4* > *APOE-4/4*. Patients with the other *APOE* genotypes (*2/2*, * 2/3*, *2/4*) did not show any hypolipemic response to this novel compound [16,194]. In patients with dementia, the effects of sardilipin were very similar to those observed in patients with chronic dyslipidemia, suggesting that the lipid-lowering properties of sardilipin are *APOE*-dependent (Figure 1).

Clinical studies have revealed that sardilipin reduces blood total cholesterol (T-CHO) (20–30%), Glu (5–10%), UA (10–15%), TG (30–50%), ALT and AST, after 1–3 months of treatment at a daily dose of 250-500 mg (t.i.d). The effect on T-CHO is the result of decreasing LDL-CHO levels and increasing HDL-CHO levels in parallel with an improvement in hepatic protection reflected by reduction in ALT, AST, and GGT activity, as the result of reducing liver steatosis. Both LDL and HDL levels are modulated by dietary, behavioral and genetic factors [16]. Most of these therapeutic effects on the regulation of lipid metabolism tend to show an age-dependent pattern and are also associated with specific genomic profiles in the population. In addition, sardilipin diminishes the size of xanthelasma plaques by 30–60% after 6–9 months of treatment, and specifically protects against the hepatotoxicity induced by statins. Similar effects can be observed on atheromatous plaques on the aortic wall of patients with familial and sporadic dyslipidemia/hyperlipidemia. The daily administration of 1000–1500 mg/day of E-SAR p.o. for three months tends to reduce the average size of atherosclerotic plaques on the aortic wall by 10%. This effect is more significant in patients harboring the *APOE-3/3* than in *APOE-3/4* carriers in whom the size of the plaque is approximately 30–40% larger than in *APOE-3/3* carriers [16,195].

## 9. Practical Considerations

The great variability in the therapeutic response of patients with CNS disorders to conventional treatments (<20% effective responders), the heterogeneity of the disease and its complex pathogenesis, the occurrence of neuropsychiatric disorders associated with cognitive deterioration, as well as the presence of other age-related disorders, seem to suggest that: (i) it is very unlikely that a single drug may be able to halt disease progression after the onset of the disease; (ii) multifactorial interventions (as in other complex disorders, such as cardiovascular disease, cancer, AIDS, *etc*.) might be an alternative strategy; however, drug-drug interactions in patients who receive over 6 different drugs per day can represent a serious drawback in terms of safety; (iii) the co-administration of many different drugs in patients with concomitant pathologies (*i.e.*, coronary disease, hypertension, atherosclerosis, hyperlipidemia, dementia) may represent an obstacle for an effective pharmacological management of CNS disorders since some drugs effective for a peripheral medical condition can exert a deleterious effect on brain function and brain perfusion with severe effects on cognition, behavior and psychomotor function; (iv) the fact that approximately 50–60% of patients with dementia exhibit a marked cerebrovascular dysfunction recommends that cerebrovascular protection should not be neglected in the treatment of AD; (v) the co-administration of psychotropic drugs should be carried out with extreme care as most psychotropics deteriorate cognitive function, psychomotor activity, and cerebrovascular function; (vi) the conventional procedures currently used in drug development (*i.e.*, trial-and-error) and serendipity are not cost-effective nowadays; (vii) the bimodal fashion of the amyloid-tau hypothesis of AD as a major target for future drug developments is a focus of controversy with unpredictable consequences for the industry and the public; (viii) the reluctant attitude of the medical community to incorporate genomic procedures as diagnostic aids and disease biomarkers is not contributing to accelerating our understanding of CNS disorders and their biological diversity; and (ix) the underdeveloped field of pharmacogenetics and pharmacogenomics is delaying the possibility of optimizing our limited therapeutic resources for the treatment of neuropsychiatric disorders and dementia [7]. 

The introduction of novel procedures into an integral genomic medicine protocol for CNS disorders is an imperative requirement in drug development and in clinical practice in order to improve diagnostic accuracy and to optimize therapeutics. This kind of protocol should integrate the following components: (i) clinical history; (ii) laboratory tests; (iii) neuropsychological assessment; (iv) cardiovascular evaluation; (v) conventional X-ray technology; (vi) structural neuroimaging; (vii) functional neuroimaging; (viii) computerized brain electrophysiology; (ix) cerebrovascular evaluation; (x) structural genomics; (xi) functional genomics; (xii) pharmacogenetics; (xiii) pharmacogenomics; (xiv) nutrigenetics; (xv) nutrigenomics; (xvi) bioinformatics for data management; and (xvii) artificial intelligence procedures for diagnostic assignments and probabilistic therapeutic options [6]. All these procedures, under personalized strategies adapted to the complexity of each case, are essential in order to depict a clinical profile based on specific biomarkers correlating with individual genomic profiles.

Our understanding of the pathophysiology of CNS disorders has advanced dramatically during the last 30 years, especially in terms of their molecular pathogenesis and genetics. The drug treatment of CNS disorders has also taken remarkable strides, with the introduction of many new drugs for the treatment of SCZ, depression, anxiety, epilepsy, Parkinson’s disease, and AD, among many other quantitatively and qualitatively important neuropsychiatric disorders. Improvement in terms of clinical outcome, however, has fallen short of expectations, with up to one third of the patients continuing to experience clinical relapse or unacceptable medication-related side-effects in spite of efforts to identify optimal treatment regimes with one or more drugs. Potential reasons to explain this historical setback might be that: (i) the molecular pathology of most CNS disorders is still poorly understood; (ii) drug targets are inappropriate, not fitting into the real etiology of the disease; (iii) most treatments are symptomatic, but not anti-pathogenic; (iv) the genetic component of most CNS disorders is poorly defined; and (v) the understanding of genome-drug interactions is very limited [6,7].

The optimization of CNS therapeutics requires the establishment of new postulates regarding (i) the costs of medicines; (ii) the assessment of protocols for multifactorial treatment in chronic disorders; (iii) the implementation of novel therapeutics addressing causative factors; and (iv) the setting-up of pharmacogenetic/pharmacogenomic strategies for drug development [7].

The cost of medicines is a highly important issue in many countries due to (i) the growth of the aging population (>5% disability), (ii) neuropsychiatric and demented patients (>5% of the population) belonging to an unproductive sector with low income, and (iii) the high cost of healthcare systems and new health technologies in developed countries. Despite the efforts of the pharmaceutical industry to demonstrate the benefits and cost-effectiveness of available drugs, the general impression in the medical community and in some governments is that some psychotropics and most anti-dementia drugs present in the market are not cost-effective [4]. Conventional drugs for neuropsychiatric disorders are relatively simple compounds with unreasonable prices. Some new products are not superior to conventional antidepressants, neuroleptics, and anxiolytics. There is an urgent need to assess the costs of new trials with pharmacogenetic and pharmacogenomic strategies, and to implement pharmacogenetic procedures for the prediction of drug-related adverse events. Pharmacogenomics can also help to reduce costs in drug development as well as the number of patients in clinical trials with high risk of toxicity. It has been suggested that the two critical strategies for pipeline genetics must make use of fewer patients: (i) the early identification of efficacy signals so that they can be applied early in development for targeted therapies; and (ii) identification of safety signals which can subsequently be validated prospectively during development using the least number of patients with adverse responses [190]. 

Cost-effectiveness analysis has been the most commonly applied framework for evaluating pharmacogenetics. Pharmacogenetic testing is potentially relevant to large populations which incur in high costs. For instance, the most common drugs metabolized by *CYP2D6* account for 189 million prescriptions and US$12.8 billion annually in expenditures in the US, which represent 5–10% of total utilization and expenditures for outpatient prescription drugs [196]. Pharmacogenomics offer great potential to improve patients’ health in a cost-effective manner; however, pharmacogenetics/pharmacogenomics will not be applied to all drugs available in the market, and careful evaluations should be made prior to investing resources in R&D of pharmacogenomic-based therapeutics and making reimbursement decisions [197].

In performing pharmacogenomic studies in dementia, it is necessary to rethink the therapeutic expectations of novel drugs, redesign the protocols for drug clinical trials, and incorporate biological markers as assessable parameters of efficacy and prevention. In addition to the characterization of genomic profiles, phenotypic profiling of responders and non-responders to conventional drugs is also important (and currently neglected). 

An important issue in AD therapeutics is that anti-dementia drugs should be effective in covering the clinical spectrum of dementia symptoms represented by memory deficits, behavioral changes, and functional decline. It is difficult (or impossible) for a single drug to be able to fulfil these criteria. A potential solution to this problem is the implementation of cost-effective, multifactorial (combination) treatments integrating several drugs, taking into consideration that traditional neuroleptics and novel antipsychotics (and many other psychotropics) deteriorate both cognitive and psychomotor functions in the elderly and may also increase the risk of stroke [198]. Few studies with combination treatments have been reported and most of them are poorly designed. We must also realize that the vast majority of dementia cases in people older than 75–80 years are of a mixed type, in which the cerebrovascular component associated with neurodegeneration cannot be therapeutically neglected. In most cases of dementia, the multifactorial (combination) therapy appears to be the most effective strategy [6,7,99,100,101,102]. The combination of several drugs increases the direct costs (e.g., medication) by 5–10%, but in turn, annual global costs are reduced by approximately 18-20% and the average survival rate increases by about 30% (from 8 to 12 years post-diagnosis) [4,7]. 

There are major concerns regarding the validity of clinical trials in patients with severe dementia. If we assume that AD is a complex disorder where genomic and environmental factors interact to induce the premature death of neurons (which begins 30 years prior to the onset of the disease), it seems clear that future therapeutic strategies must be addressed towards the prevention of neurodegeneration because when the first symptoms appear thousands of millions of neurons have already died, and under these circumstances the possibility of being therapeutically effective is very remote. 

Major impact factors associated with drug efficacy and safety include the following: (i) the mechanisms of action of drugs; (ii) drug-specific adverse reactions; (iii) drug-drug interactions; (iv) nutritional factors; (v) vascular factors; (vi) social factors; and (vii) genomic factors (nutrigenetics, nutrigenomics, pharmacogenetics, pharmacogenomics). Among genomic factors, nutrigenetics/nutrigenomics and pharmacogenetics/pharmacogenomics account for over 80% of efficacy-safety outcomes in current therapeutics [6,7,11,102,104].

To achieve a mature discipline of pharmacogenetics and pharmacogenomics in CNS disorders and dementia it would be convenient to accelerate the following processes: (i) to educate physicians and the public on the use of genetic/genomic screening in the daily clinical practice; (ii) to standardize genetic testing for major categories of drugs; (iii) to validate pharmacogenetic and pharmacogenomic procedures according to drug category and pathology; (iv) to regulate ethical, social, and economic issues; and (v) to incorporate pharmacogenetic and pharmacogenomic procedures to both drugs in development and drugs on the market in order to optimize therapeutics [6,7,8,9,10,11,12,13,14,15,16,100,101,102,104].

## 10. Future Trends

The globalization of the present economic crisis will negatively affect future investments in CNS research and drug development; however, for the first half of the coming decade, after an initial period with some programmes put on standby, it is expected that progress in the pathogenesis of CNS disorders, molecular diagnosis, and therapeutics will evolve favorably. Genome-wide family-based association studies, using single SNPs or haplotypes, will help to identify associations with genome-wide significance [47,48,199,200]; similarly, genome-wide expression analysis will be useful for the discovery of new drug targets. Some studies will try to elucidate the weight of genome-environment interactions in the pathogenesis and clinical course of CNS disorders, and also the emerging role of epigenetics. The validation of protocols for genomic screening will contribute to introducing structural genomics (genotyping, genome-wide analysis), functional genomics (genotype-phenotype correlations), and proteomics as diagnostic aids and therapeutic targets [201]. New initiatives for the prevention of dementia will also emerge [202], together with new insights into the role of nutrition and nutrigenomics in brain function and neurodegeneration [203].

Priority areas for pharmacogenetic research are to predict serious adverse reactions (ADRs) and to establish variation in efficacy [204]. Both requirements are necessary in psychotic disorders and dementia to cope with efficacy and safety issues associated with current antipsychotics and anti-dementia drugs, and new CNS drugs as well. Since drug response is a complex trait, genome-wide approaches (oligonucleotide microarrays, proteomic profiling) may provide new insights into drug metabolism and drug response. Of paramount importance is the identification of polymorphisms affecting gene regulation and mRNA processing in genes encoding cytochrome P450s and other drug-metabolizing enzymes, drug transporters, and drug targets and receptors, with broad implication in pharmacogenetics since functional polymorphisms which alter gene expression and mRNA processing appear to play a critical role in shaping human phenotypic variability [205]. It is also most relevant, from a practical point of view, to understand the pharmacogenomics of drug transporters, especially *ABCB1* (P-glycoprotein/MDR1) variants, due to the pleiotropic activity of this gene on a large number of drugs [206]. There are over 170 human solute carrier transporters which transport a variety of substrates, including amino acids, lipids, inorganic ions, peptides, saccharides, metals, drugs, toxic xenobiotics, chemical compounds, and proteins [207].

In approximately 3–5 years novel data on clinical trials with anti-amyloid vaccines will be delivered and AD immunotherapy will face new vaccine models (active and passive immunization) and new therapeutic challenges regarding the amyloid burden in AD [208]. Other expected developments in AD therapeutics include γ-secretase inhibitors, β-secretase inhibitors, β-sheet breakers and chaperone inhibitors, regulators of the ubiquitin-proteasome system, small molecule activators (non-peptide neurotrophic factors) of the Trk receptors, p38α mitogen-activated protein kinase (MAPK) regulators, ADNP (activity-dependent neuroprotective protein) derivatives (NAP peptides), GSK-3β modulators, phospholipase A2 inhibitors, the medium-chain triglyceride AC-1202, inhibitors of insulin-regulated aminopeptidase, amphiphilic pyridinium salts, and some other novel compounds, still in a preclinical stage, most of which are intended to be Aβ lowering agents. There will be some initiatives for nanotechnology approaches to crossing the blood-brain barrier and drug delivery to the CNS, as well as for new transdermal and intranasal delivery systems. 

Another important issue in the pathogenesis and therapeutics of CNS disorders and dementia is the role of microRNAs (miRNAs), RNA interference (RNAi) and gene silencing. Double-stranded RNA-mediated interference (RNAi) is a simple and rapid method of silencing gene expression in different organisms. The silencing of a gene is a consequence of degradation of RNA into short RNAs that activate ribonucleases to target homologous mRNA. Genetic and biochemical studies revealed a two-step mechanism of RNAi-induced gene silencing: (i) degradation of dsRNA into small interfering RNAs (siRNAs), 21 to 25 nucleotides long, by an RNase III-like activity; (ii) the siRNAs join an RNase complex, RISC (RNA-induced silencing complex), which acts on the cognate mRNA and degrades it. Key components such as Dicer, RNA-dependent RNA polymerase, helicases, and dsRNA endonucleases play important roles in RNAi. Some of these components also control the development of many organisms by processing many noncoding RNAs, called micro-RNAs. In the context of RNAi, the genome also undergoes alterations in the form of DNA methylation, heterochromatin formation, and programmed DNA elimination. RNAi is being considered as an important tool for functional genomics and for gene-specific therapeutic activities that target the mRNAs of disease-related genes [209,210,211].

Nearly 97% of the human genome is non-coding DNA, and introns occupy most of it around the gene-coding regions. Numerous intronic sequences have been found to encode microRNAs, which are responsible for RNA-mediated gene silencing through RNA interference (RNAi)-like pathways. microRNAs (miRNAs), small single-stranded regulatory RNAs capable of interfering with intracellular messenger RNAs (mRNAs) that contain either complete or partial complementarity, are useful for the design of new therapies. miRNAs were firstly discovered in *Caenorhabditis elegans* as native RNA fragments that modulate a wide range of genetic regulatory pathways during embryonic development. Intronic microRNA is a new class of miRNAs derived from the processing of gene introns. The intronic miRNAs differ uniquely from previously described intergenic miRNAs in the requirement of type II RNA polymerases (Pol-II) and spliceosomal components for their biogenesis. There is an evolutionary preservation of the intron-mediated gene silencing through miRNA functionality in cell and *in vivo*, suggesting the existence of an intracellular miRNA-mediated gene regulatory system, fine-tuning the degradation of protein-coding messenger RNAs [212]. New inventories of miRNA expression profiles from CNS regions will be reported in the near future. These inventories of CNS miRNA profiles will provide an important step toward further elucidation of miRNA function and miRNA-related gene regulatory networks in the mammalian CNS. RNAi has led in recent years to powerful approaches to silencing targeted genes in a sequence-specific manner with potential therapeutic applications in neurodegenerative diseases. RNAi procedures for gene-selective inhibition must improve (i) cytoplasmic delivery of short sdRNA oligonucleotides (siRNA), which mimics an active intermediate of an endogenous RNAi mechanism; and (ii) nuclear delivery of gene expression cassettes which express a short hairpin RNA (shRNA), which mimics the micro interfering RNA (miRNA) active intermediate of a different endogenous RNAi mechanism. 

These technologies, complemented by non-viral gene delivery systems and ligand-targeted plasmid-based nanoparticles for RNAi agents, will bring new hopes for the treatment of different complex disorders [213,214]. We need more information about the feasibility of targeting AD genes (e.g., *APP*-London mutation, *APP*-Swedish mutation, *PS1*, *APLP1*, *APLP2*, *PEN-2*, *APH-1a*, *Nicastrin*, *BACE*, *MAPT*-V337M) with RNAi and making sure that gene silence in CNS disorders does not affect proteomic and/or metabolomic networks which are fundamental for a correct brain function [215]. 

Another area of growing interest is the role of adult neurogenesis and stem cells in AD. Stem cell therapy has been suggested as a possible strategy for replacing damaged circuitry and restoring learning and memory abilities in patients with AD and other neurodegenerative disorders; however, there is a long path ahead from the promising investigations which are raising hopes, and the challenges behind translating underlying stem cell biology into an effective therapy for CNS disorders and dementia [216].

## References

[1] Andlin-Sobocki P., Jönsson B., Wittchen H.-U., Olesen J. (2005). Costs of disorders of the brain in Europe. Executive summary. Eur. J. Neurol..

[2] Sousa R.M., Ferri C.P., Acosta D., Albanese E., Guerra M., Huang Y., Jacob K.S., Jotheeswaran A.T., Rodríguez J.J., Pichardo G.R. (2009). Contribution of chronic diseases to disability in elderly people in countries with low and middle incomes: a 10/66 Dementia Research Group population-based survey. Lancet.

[3] Cacabelos R. (2001). Psychogeriatric research. A conceptual introduction to geriatric neuroscience. Psychogeriatrics.

[4] Loveman E., Green C., Kirby J., Takeda A., Picot J., Payne E., Clegg A. (2006). The clinical and cost-effectiveness of donepezil, rivastigmine, galantamine and memantine for Alzheimer’s disease. Health Technol. Assess..

[5] Cacabelos R., Álvarez A., Lombardi V., Fernández-Novoa L., Corzo L., Pérez P., Laredo M., Pichel V., Hernández A., Varela M. (2000). Pharmacological treatment of Alzheimer disease: From psychotropic drugs and cholinesterase inhibitors to pharmacogenomics. Drugs Today.

[6] Cacabelos R., Ritsner M.S. (2009). Pharmacogenomic biomarkers in neuropsychiatry: The path to personalized medicine in mental disorders. The Handbook of Neuropsychiatric Biomarkers, Endophenotypes and Genes.

[7] Cacabelos R. (2009). Pharmacogenomics and therapeutic strategies for dementia. Expert Rev. Mol. Diag..

[8] Cacabelos R. (2005). Molecular genetics of Alzheimer’s disease and aging. Meth. Find. Exper. Clin. Pharmacol..

[9] Berry N., Jobanputra V., Pal H. (2003). Molecular genetics of schizophrenia: a critical review. J Psychiatry Neurosci..

[10] Kato T. (2007). Molecular genetics of bipolar disorder and depression. Psychiatry Clin. Neurosci..

[11] Cacabelos R. (2008). Pharmacogenomics in Alzheimer’s disease. Meth. Mol. Biol..

[12] Cacabelos R. (2002). Pharmacogenomics for the treatment of dementia. Ann. Med..

[13] Cacabelos R. (2003). The application of functional genomics to Alzheimer’s disease. Pharmacogenomics.

[14] Cacabelos R. (2005). Pharmacogenomics and therapeutic prospects in Alzheimer’s disease. Exp. Opin. Pharmacother..

[15] Cacabelos R. (2005). Pharmacogenomics, nutrigenomics and therapeutic optimization in Alzheimer’s disease. Aging Health.

[16] Cacabelos R., Takeda M. (2006). Pharmacogenomics, nutrigenomics and future therapeutics in Alzheimer’s disease. Drugs of the Future.

[17] Roses A.D. (2004). Pharmacogenetics and drug development: the path to safer and more effective drugs. Nat. Rev. Genet..

[18] Sharp A.J., Cheng Z., Eichler E.E. (2006). Structural variation of human genome. Annu. Rev. Genomics Hum. Genet..

[19] Crawford D.C., Akey D.T., Nickerson D.A. (2005). The patterns of natural variation in human genes. Annu. Rev. Genomics Hum. Genet..

[20] NCBI. http://www.ncbi.nlm.nih.gov/OMIM/.

[21] Alzheimer Research Forum. http://www.alzgene.org/.

[22] National Human Genome Research Institute. HUGO Gene Nomenclature Committee. http://www.genenames.org.

[23] Selkoe D.J., Podlisny M.B. (2002). Deciphering the genetic basis of Alzheimer’s disease. Annu. Rev. Genomics Hum. Genet..

[24] Suh Y.-H., Checler F. (2002). Amyloid precursor protein, presenilins, and α-synuclein: Molecular pathogenesis and pharmacological applications in Alzheimer’s disease. Phamacol. Rev..

[25] Cacabelos R. (2007). Donepezil in Alzheimer’s disease: From conventional trials to pharmacogenetics. Neuropsychiat. Dis. Treat..

[26] Strittmatter W.J., Saunders A.M., Schmechel D., Pericak-Vance M., Enghild J., Salvesen G.S., Roses A.D. (1993). Apolipoprotein E: high-avidity binding to beta-amyloid and increased frequency of type 4 allelein late-onset familial Alzheimer disease. Proc. Nat. Acad. Sci. USA.

[27] Saunders A.M., Schmader K., Breitner J.C., Benson M.D., Brown W.T., Goldfarb L., Goldgaber D., Manwaring M.G., Szymanski M.H., McCown N. (1993). Apolipoprotein E epsilon-4 allele distributions in late-onset Alzheimer’s disease and in other amyloid-forming diseases. Lancet.

[28] Saunders A.M., Strittmatter W.J., Schamechel D., St. George-Hyslop M.D., Pericak-Vance M.A., Joo S.H., Rosi B.L., Gusella J.F., Crapper-MacLachlan D.R., Alberts M.J. (1993). Association of apolipoprotein E allele E4 with late-onset familial and sporadic Alzheimer’s disease. Neurology.

[29] Corder E.H., Saunders A.M., Risch N.J., Strittmatter W.J., Schmechel D.E., Gaskell P.C., Rimmler J.B., Locke P.A., Conneally P.M., Schmader K.E. (1993). Gene dosage of apolipoprotein E type 4 allele and risk of Alzheimer’s disease in late onset families. Science.

[30] Corder E.H., Saunders A.M., Risch N.J., Strittmatter W.J., Schmechel D.E., Gaskell P.C., Rimmler J.B., Locke P.A., Conneally P.M., Schmader K.E. (1994). Protective effect of apolipoprotein E type 2 allele for late onset Alzheimer disease. Nature Genet..

[31] Schächter F., Faure-Delanef L., Guénot F., Rouger H., Froguel P., Lesueur-Ginot L., Cohen D. (1994). Genetic association with human longevity at the APOE and ACE loci. Nature Genet..

[32] Roses A.D. (2000). Pharmacogenetics and future drug development and delivery. Lancet.

[33] Roses A.D. (2000). Pharmacogenetics and the practice of medicine. Nature.

[34] Filippini N., Ebmeier K.P., Macintosh B.J., Trachtenberg A.J., Frisoni G.B., Wilcock G.K., Beckmann C.F., Smith S.M., Matthews P.M., Mackay C.E. (2010). Differential effects of the APOE genotype on brain function across the lifespan. Neuroimage.

[35] Rogaeva E., Meng Y., Lee J.H., Gu Y., Kawarai T., Zou F., Katayama T., Baldwin C.T., Cheng R., Hasegawa H. (2007). The neuronal sortilin-related receptor SORL1 is genetically associated with Alzheimer’s disease. Nat. Genet..

[36] Meng Y., Lee J.H., Cheng R., St George-Hyslop P., Mayeux R., Farrer L.A. (2007). Association between SORL1 and Alzheimer disease in a genome-wide study. Neuroreport.

[37] Shibata N., Ohnuma T., Baba H., Higashi S., Nishioka K., Arai H. (2008). Genetic association between SORL1 polymorphisms and Alzheimer’s disease in a Japanese population. Dement. Geriatr. Cogn. Disord..

[38] Rohe M., Synowitz M., Glass R., Paul S.M., Nykjaer A., Willnow T.E. (2009). Brain-derived neurotrophic factor reduces amyloidogenic processing through control of SORLA gene expression. J. Neurosci..

[39] Fehér A., Juhász A., Rimanóczy A., Kálmán J., Janka Z. (2009). Association between BDNF Val66Met polymorphism and Alzheimer disease, dementia with Lewy bodies, and Pick disease. Alzheimer Dis. Assoc. Disord..

[40] Wang Y., Rogers P.M., Stayrook K.R., Su C., Varga G., Shen Q., Nagpal S., Burris T.P. (2008). The selective Alzheimer’s disease indicator-1 gene (Seladin-1/DHCR24) is a liver X receptor target gene. Mol. Pharmacol..

[41] Peri A., Serio M. (2008). Neuroprotective effects of the Alzheimer’s disease gene seladin-1. J. Mol. Endocrinol..

[42] Sanders A.E., Wang C., Katz M., Derby C.A., Barzilai N., Ozelius L., Lipton R.B. (2010). Association of a functional polymorphism in the cholesteryl ester transfer protein (CETP) gene with memory decline and incidence of dementia. JAMA.

[43] Schaffer B.A., Bertram L., Miller B.L., Mullin K., Weintraub S., Johnson N., Bigio E.H., Mesulam M., Wiedau-Pazos M., Jackson G.R. (2008). Association of GSK3B with Alzheimer’s disease and frontotemporal dementia. Arch. Neurol..

[44] Hsu W.C., Wang H.K., Lee L.C., Fung H.C., Lin J.C., Hsu H.P., Wu Y.R., Ro L.S., Hu F.J., Chang Y.T. (2008). Promoter polymorphisms modulating HSPA5 expression may increase susceptibility to Taiwanese Alzheimer’s disease. J. Neural. Transm..

[45] Chen Y., Jia L., Wei C., Wang F., Lv H., Jia J. (2008). Association between polymorphisms in the apolipoprotein D gene and sporadic Alzheimer’s disease. Brain Res..

[46] Dreses-Werringloer U., Lambert J.C., Vingtdeux V., Zhao H., Vais H., Siebert A., Jain A., Koppel J., Rovelet-Lecrux A., Hannequin D. (2008). A polymorphism in CALHM1 influences Ca^2+^ homeostasis, Aβ levels, and Alzheimer’s disease risk. Cell.

[47] Harold D., Abraham R., Hollingworth P., Sims R., Gerrish A., Hamshere M.L., Pahwa J.S., Moskvina V., Dowzell K., Williams A. (2009). Genome-wide association study identifies variants at CLU and PICALM associated with Alzheimer’s disease. Nat. Genet..

[48] Lambert J.C., Heath S., Even G., Campion D., Sleegers K., Hiltunen M., Combarros O., Zelenika D., Bullido M.J., Tavernier B. (2009). Genome-wide association study identifies variants at CLU and CR1 associated with Alzheimer’s disease. Nat. Genet..

[49] Jun G., Naj A.C., Beecham G.W., Wang L.S., Buros J., Gallins P.J., Buxbaum J.D., Ertekin-Taner N., Fallin M.D., Friedland R. (2010). Meta-analysis Confirms CR1, CLU, and PICALM as Alzheimer Disease Risk Loci and Reveals Interactions With APOE Genotypes. Arch. Neurol..

[50] Seshadri S., Fitzpatrick A.L., Ikram M.A., DeStefano A.L., Gudnason V., Boada M., Bis J.C., Smith A.V., Carassquillo M.M., Lambert J.C. (2010). Genome-wide analysis of genetic loci associated with Alzheimer disease. JAMA.

[51] Kramer P.L., Xu H., Woltjer R.L., Westaway S.K., Clark D., Erten-Lyons D., Kaye J.A., Welsh-Bohmer K.A., Troncoso J.C., Markesbery W.R. (2010). Alzheimer disease pathology in cognitively healthy elderly: A genome-wide study. Neurobiol. Aging..

[52] Durakoglugil M.S., Chen Y., White C.L., Kavalali E.T., Herz J. (2009). Reelin signaling antagonizes beta-amyloid at the synapse. Proc. Natl. Acad. Sci. U.S.A.

[53] Roses A.D., Lutz M.W., Amrine-Madsen H., Saunders A.M., Crenshaw D.G., Sundseth S.S., Huentelman M.J., Welsh-Bohmer K.A., Reiman E.M. (2009). A TOMM40 variable-length polymorphism predicts the age of late-onset Alzheimer’s disease. Pharmacogenomics J..

[54] Lutz M.W., Crenshaw D.G., Saunders A.M., Roses A.D. (2010). Genetic variation at a single locus and age of onset for Alzheimer’s disease. Alzheimers Demen..

[55] Roses A.D. (2010). An inherited variable poly-T repeat genotype in TOMM40 in Alzheimer disease. Arch. Neurol..

[56] Ohe K., Mayeda A. (2010). HMGA1a trapping of U1 snRNP at an authentic 5' splice site induces aberrant exon skipping in sporadic Alzheimer’s disease. Mol. Cell. Biol..

[57] Kelley B.J., Haidar W., Boeve B.F., Baker M., Shiung M., Knopman D.S., Rademakers R., Hutton M., Adamson J., Kuntz K.M., Dickson D.W., Parisi J.E., Smith G.E., Petersen R.C. (2010). Alzheimer disease-like phenotype associated with the c.154delA mutation in progranulin. Arch. Neurol..

[58] Yu C.E., Bird T.D., Bekris L.M., Montine T.J., Leverenz J.B., Steinbart E., Galloway N.M., Feldman H., Woltjer R., Miller C.A. (2010). The spectrum of mutations in progranulin: a collaborative study screening 545 cases of neurodegeneration. Arch. Neurol..

[59] Smach M.A., Charfeddine B., Othman L.B., Lammouchi T., Ltaief A., Nafati S., Dridi H., Bennamou S., Limem K. (2010). -1154G/A and -2578C/A polymorphisms of the vascular endothelial growth factor gene in Tunisian Alzheimer patients in relation to beta-amyloid (1-42) and total tau protein. Neurosci. Lett..

[60] Jin Z., Luxiang C., Huadong Z., Yanjiang W., Zhiqiang X., Hongyuan C., Lihua H., Xu Y. (2009). Endothelin-converting enzyme-1 promoter polymorphisms and susceptibility to sporadic late-onset Alzheimer’s disease in a Chinese population. Dis. Markers.

[61] Xue S., Jia L., Jia J. (2009). Association between somatostatin gene polymorphisms and sporadic Alzheimer’s disease in Chinese population. Neurosci. Lett..

[62] Li K., Dai D., Zhao B., Yao L., Yao S., Wang B., Yang Z. (2010). Association between the RAGE G82S polymorphism and Alzheimer’s disease. J. Neural. Transm..

[63] Takuma K., Fang F., Zhang W., Yan S., Fukuzaki E., Du H., Sosunov A., McKhann G., Funatsu Y., Nakamichi N. (2009). RAGE-mediated signaling contributes to intraneuronal transport of amyloid-beta and neuronal dysfunction. Proc. Natl. Acad. Sci. USA.

[64] Shibata N., Ohnuma T., Baba H., Arai H. (2009). Genetic association analysis between TDP-43 polymorphisms and Alzheimer’s disease in a Japanese population. Dement. Geriatr. Cogn. Disord..

[65] Listì F., Caruso C., Lio D., Colonna-Romano G., Chiappelli M., Licastro F., Candore G. (2010). Role of cyclooxygenase-2 and 5-lipoxygenase polymorphisms in Alzheimer’s disease in a population from northern Italy: implication for pharmacogenomics. J. Alzheimers Dis..

[66] Guerini F.R., Tinelli C., Calabrese E., Agliardi C., Zanzottera M., de Silvestri A., Franceschi M., Grimaldi L.M., Nemni R., Clerici M. (2009). HLA-A*01 is associated with late onset of Alzheimer’s disease in Italian patients. Int. J. Immunopathol. Pharmaco..

[67] Vural P., Değirmencioğlu S., Parildar-Karpuzoğlu H., Doğru-Abbasoğlu S., Hanagasi H.A., Karadağ B., Gürvit H., Emre M., Uysal M. (2009). The combinations of TNFalpha-308 and IL-6 -174 or IL-10 -1082 genes polymorphisms suggest an association with susceptibility to sporadic late-onset Alzheimer’s disease. Acta Neurol. Scand..

[68] Capurso C., Solfrizzi V., Colacicco A.M., D’Introno A., Frisardi V., Imbimbo B.P., Lorusso M., Vendemiale G., Denitto M., Santamato A. (2010). Interleukin 6-174 G/C promoter and variable number of tandem repeats (VNTR) gene polymorphisms in sporadic Alzheimer’s disease. Prog. Neuropsychopharmacol. Biol. Psychiatry.

[69] Yu J.T., Song J.H., Wang N.D., Wu Z.C., Zhang Q., Zhang N., Zhang W., Xuan S.Y., Tan L. (2010). Implication of IL-33 gene polymorphism in Chinese patients with Alzheimer's disease. Neurobiol. Aging.

[70] Butler H.T., Warden D.R., Hogervorst E., Ragoussis J., Smith A.D., Lehmann D.J. (2010). Association of the aromatase gene with Alzheimer’s disease in women. Neurosci. Lett..

[71] Vepsäläinen S., Helisalmi S., Mannermaa A., Pirttilä T., Soininen H., Hiltunen M. (2009). Combined risk effects of IDE and NEP gene variants on Alzheimer disease. J. Neurol. Neurosurg. Psychiatry.

[72] Zhong L., Dong-hai Q., Hong-ying L., Qing-feng L. (2009). Analysis of the nicastrin promoter rs10752637 polymorphism and its association with Alzheimer’s disease. Eur. J. Neurosci..

[73] Laumet G., Petitprez V., Sillaire A., Ayral A.M., Hansmannel F., Chapuis J., Hannequin D., Pasquier F., Scarpini E., Galimberti D. (2010). A study of the association between the ADAM12 and SH3PXD2A (SH3MD1) genes and Alzheimer’s disease. Neurosci. Lett..

[74] Leduc V., Théroux L., Dea D., Robitaille Y., Poirier J. (2009). Involvement of paraoxonase 1 genetic variants in Alzheimer’s disease neuropathology. Eur. J. Neurosci..

[75] Liu H.P., Lin W.Y., Liu S.H., Wang W.F., Tsai C.H., Wu B.T., Wang C.K., Tsai F.J. (2009). Genetic variation in N-methyl-D-aspartate receptor subunit NR3A but not NR3B influences susceptibility to Alzheimer’s disease. Dement. Geriatr. Cogn. Disord..

[76] di Maria E., Bonvicini C., Bonomini C., Alberici A., Zanetti O., Gennarelli M. (2009). Genetic variation in the G720/G30 gene locus (DAOA) influences the occurrence of psychotic symptoms in patients with Alzheimer’s disease. J. Alzheimers Dis..

[77] Martínez M.F., Martín X.E., Alcelay L.G., Flores J.C., Valiente J.M., Juanbeltz B.I., Beldarraín M.A., López J.M., Gonzalez-Fernández M.C., Salazar A.M. (2009). The COMT Val158 Met polymorphism as an associated risk factor for Alzheimer disease and mild cognitive impairment in APOE 4 carriers. BMC Neurosc..

[78] Maruszak A., Safranow K., Gustaw K., Kijanowska-Haładyna B., Jakubowska K., Olszewska M., Styczyńska M., Berdyński M., Tysarowski A., Chlubek D. (2009). PIN1 gene variants in Alzheimer’s disease. BMC Med. Genet..

[79] Kellett K.A., Hooper N.M. (2009). Prion protein and Alzheimer disease. Prion.

[80] Lloyd S.E., Rossor M., Fox N., Mead S., Collinge J. (2009). HECTD2, a candidate susceptibility gene for Alzheimer’s disease on 10q. BMC Med. Genet..

[81] Ahn K., Song J.H., Kim D.K., Park M.H., Jo S.A., Koh Y.H. (2009). Ubc9 gene polymorphisms and late-onset Alzheimer’s disease in the Korean population: a genetic association study. Neurosci. Lett..

[82] Persengiev S., Kondova I., Otting N., Koeppen A.H., Bontrop R.E. (2010). Genome-wide analysis of miRNA expression reveals a potential role for miR-144 in brain aging and spinocerebellar ataxia pathogenesis. Neurobiol. Aging.

[83] Bettens K., Brouwers N., van Miegroet H., Gil A., Engelborghs S., de Deyn P.P., Vandenberghe R., van Broeckhoven C., Sleegers K. (2010). Follow-up study of susceptibility Loci for Alzheimer’s disease and onset age identified by genome-wide association. J. Alzheimers Dis..

[84] Donkin J.J., Stukas S., Hirsch-Reinshagen V., Namjoshi D., Wilkinson A., May S., Chan J., Fan J., Collins J., Wellington C.L. (2010). ATP-binding cassette transporter A1 mediates the beneficial effects of the liver-X-receptor agonist GW3965 on object recognition memory and amyloid burden in APP/PS1 mice. J. Biol. Chem..

[85] Kuerban B., Shibata N., Komatsu M., Ohnuma T., Arai H. (2010). Genetic Association between PLTP Gene Polymorphisms and Alzheimer's Disease in a Japanese Population. Dement. Geriatr. Cogn. Disord..

[86] Bertram L., Tanzi R.E. (2009). Genome-wide association studies in Alzheimer’s disease. Hum. Mol. Genet..

[87] Lin M.T., Simon D.K., Ahn C.H., Kim L.M., Beal M.F. (2002). High aggregate burden of somatic mtDNA point mutations in aging and Alzheimer’s disease brain. Hum. Mol. Genet..

[88] Pinho C.M., Björk B.F., Alikhani N., Bäckman H.G., Eneqvist T., Fratiglioni L., Glaser E., Graff C. (2010). Genetic and biochemical studies of SNPs of the mitochondrial A beta-degrading protease, hPreP. Neurosci. Lett..

[89] Vitali M., Venturelli E., Galimberti D., Benerini Gatta L., Scarpini E., Finazzi D. (2009). Analysis of the genes coding for subunit 10 and 15 of cytochrome c oxidase in Alzheimer’s disease. J. Neural. Transm..

[90] Takasaki S. (2009). Mitochondrial haplogroups associated with Japanese Alzheimer’s patients. J. Bioenerg. Biomembr..

[91] Santoro A., Balbi V., Balducci E., Pirazzini C., Rosini F., Tavano F., Achilli A., Siviero P., Minicuci N., Bellavista E. (2010). Evidence for sub-haplogroup h5 of mitochondrial DNA as a risk factor for late onset Alzheimer's disease. PLoS One.

[92] Zhang M., Poplawski M., Yen K., Cheng H., Bloss E., Zhu X., Patel H., Mobbs C.V. (2009). Role of CBP and SATB-1 in aging, dietary restriction, and insulin-like signaling. PLoS Biol..

[93] Lukens J.N., van Deerlin V., Clark C.M., Xie S.X., Johnson F.B. (2009). Comparisons of telomere lengths in peripheral blood and cerebellum in Alzheimer’s disease. Alzheimers Dement..

[94] Zekry D., Herrmann F.R., Irminger-Finger I., Graf C., Genet C., Vitale A.M., Michel J.P., Gold G., Krause K.H. (2010). Telomere length and ApoE polymorphism in mild cognitive impairment, degenerative and vascular dementia. J. Neurol. Sci..

[95] Anderson C.N.G., Grant S.G.N. (2006). High throughput protein expression screening in the nervous system-needs and limitations. J. Physiol..

[96] Xu X., Zhan M., Duan W., Prabhu V., Brenneman R., Wood W., Firman J., Li H., Zhang P., Ibe C. (2007). Gene expression atlas of the mouse central nervous system: impact and interactions of age, energy intake and gender. Genome Biol..

[97] Mastrangelo M.A., Bowers W.J. (2008). Detailed immunohistochemical characterization of temporal and spatial progression of Alzheimer’s disease-related pathologies in male triple-transgenic mice. BMC Neurosci..

[98] Rodríguez J.J., Jones V.C., Tabuchi M., Allan S.M., Knight E.M., LaFerla F.M., Oddo S., Verkhratsky A. (2008). Impaired adult neurogenesis in the dentate gyrus of a triple transgenic mouse model of Alzheimer’s disease. PLoS One.

[99] Cacabelos R. (2008). Pharmacogenomics and therapeutic prospect in dementia. Eur Arch Psychiatry Clin. Neurosci..

[100] Cacabelos R. (2007). Pharmacogenetic basis for therapeutic optimization in Alzheimer’s disease. Mol. Diag. Ther..

[101] Cacabelos R., Llovo R., Fraile C., Fernández-Novoa L. (2007). Pharmacogenetic aspects of therapy with cholinesterase inhibitors: the role of CYP2D6 in Alzheimer’s disease pharmacogenetics. Curr. Alzheimer Res..

[102] Cacabelos R. (2007). Molecular pathology and pharmacogenomics in Alzheimer’s disease: polygenic-related effects of multifactorial treatments on cognition, anxiety, and depression. Meth. Find. Exper. Clin. Pharmacol..

[103] Cacabelos R., Fernández-Novoa L., Pichel V., Lombardi V., Kubota Y., Takeda M., Takeda M., Tanaka T., Cacabelos R. (2004). Pharmacogenomic studies with a combination therapy in Alzheimer’s disease. Molecular Neurobiology of Alzheimer Disease and Related Disorders.

[104] Cacabelos R., Cohen N. (2008). Pharmacogenomics in Alzheimer’s disease. Pharmacogenomics and Personalized Medicine.

[105] Thomann P.A., Roth A.S., Dos Santos V., Toro P., Essig M., Schröder J. (2008). Apolipoprotein E polymorphism and brain morphology in mild cognitive impairment. Dement. Geriatr. Cogn. Disord..

[106] Sando S.B., Melquist S., Cannon A., Hutton M.L., Sletvold O., Saltvedt I., White L.R., Lydersen S., Aasly J.O. (2008). APOEε4 lowers age at onset and is a high risk factor for Alzheimer’s disease; A case control study from central Norway. BMC Neurology.

[107] Thambisetty M., Beason-Held L., An Y., Kraut M.A., Resnick S.M. (2010). APOE epsilon4 genotype and longitudinal changes in cerebral blood flow in normal aging. Arch. Neurol..

[108] Cacabelos R., Fernández-Novoa L., Corzo L., Pichel V., Lombardi V., Kubota Y. (2004). Genomics and phenotypic profiles in dementia: Implications for pharmacological treatment. Meth. Find. Exper. Clin. Pharmacol..

[109] Cacabelos R., Fernández-Novoa L., Lombardi V., Corzo L., Pichel V., Kubota Y. (2003). Cerebrovascular risk factors in Alzheimer’s disease: Brain hemodynamics and pharmacogenomic implications. Neurol. Res..

[110] Roher A.E., Maarouf C.L., Sue L.I., Hu Y., Wilson J., Beach T.G. (2009). Proteomics-derived cerebrospinal fluid markers of autopsy-confirmed Alzheimer’s disease. Biomarkers.

[111] Giacobini E., Giacobini E., Pepeu G. (2006). Cholinesterases in human brain: the effect of cholinesterase inhibitors on Alzheimer’s disease and related disorders. The Brain Cholinergic System in Health and Disease.

[112] Reisberg B., Doody R., Stoffler A., Schmitt F., Ferris S., Möbius H.J. (2003). Memantine in moderate-to-severe Alzheimer’s disease. N. Engl. J. Med..

[113] Schenk D.B., Seubert P., Grundman M., Black R. (2005). Aβ immunotherapy: lessons learned for potential treatment of Alzheimer’s disease. Neurodegener. Dis..

[114] Wisniewski T., Boutajangout A. (2010). Vaccination as a therapeutic approach to Alzheimer’s disease. Mt. Sinai J. Med..

[115] de Strooper B., Vassar R., Golde T. (2010). The secretases: enzymes with therapeutic potential in Alzheimer disease. Nat. Rev. Neurol..

[116] Shelton C.C., Zhu L., Chau D., Yang L., Wang R., Djaballah H., Zheng H., Li Y.M. (2009). Modulation of gamma-secretase specificity using small molecule allosteric inhibitors. Proc. Natl. Acad. Sci. USA.

[117] Lambracht-Washington D., Qu B.X., Fu M., Eagar T.N., Stüve O., Rosenberg R.N. (2009). DNA beta-amyloid(1-42) trimer immunization for Alzheimer disease in a wild-type mouse model. JAMA.

[118] Lang F., Görlach A. (2010). Heterocyclic indazole derivatives as SGK1 inhibitors, WO2008138448. Expert Opin. Ther. Pat..

[119] Sala Frigerio C., Kukar T.L., Fauq A., Engel P.C., Golde T.E., Walsh D.M. (2009). An NSAID-like compound, FT-9, preferentially inhibits gamma-secretase cleavage of the amyloid precursor protein compared to its effect on amyloid precursor-like protein 1. Biochemistry.

[120] Boado R.J., Lu J.Z., Hui E.K., Pardridge W.M. (2010). IgG-single chain Fv fusion protein therapeutic for Alzheimer’s disease: Expression in CHO cells and pharmacokinetics and brain delivery in the rhesus monkey. Biotechnol. Bioeng..

[121] Adachi H., Katsuno M., Waza M., Minamiyama M., Tanaka F., Sobue G. (2009). Heat shock proteins in neurodegenerative diseases: pathogenic roles and therapeutic implications. Int. J. Hyperthermia.

[122] Kilgore M., Miller C.A., Fass D.M., Hennig K.M., Haggarty S.J., Sweatt J.D., Rumbaugh G. (2010). Inhibitors of class 1 histone deacetylases reverse contextual memory deficits in a mouse model of Alzheimer’s disease. Neuropsychopharmacology.

[123] Hamaguchi T., Ono K., Murase A., Yamada M. (2009). Phenolic compounds prevent Alzheimer’s pathology through different effects on the amyloid-beta aggregation pathway. Am. J. Pathol..

[124] Kalinin S., Richardson J.C., Feinstein D.L. (2009). A PPARdelta agonist reduces amyloid burden and brain inflammation in a transgenic mouse model of Alzheimer’s disease. Curr. Alzheimer Res..

[125] Roshan R., Ghosh T., Scaria V., Pillai B. (2009). MicroRNAs: novel therapeutic targets in neurodegenerative diseases. Drug Discov. Today.

[126] Maxwell M.M. (2009). RNAi applications in therapy development for neurodegenerative disease. Curr. Pharm. Des..

[127] Verrils N.M. (2006). Clinical proteomics: Present and future prospects. Clin. Biochem. Rev..

[128] Doshi J.A., Shaffer T., Briesacher B.A. (2005). National estimates of medication use in nursing homes: Findings from 1997 Medicare Current Beneficiary Survey and the 1996 Medical Expenditure Survey. J. Am. Geriatr. Soc..

[129] Percudani M., Barbui C., Fortino I., Petrovich L. (2005). Antidepressant drugs prescribing among elderly subjects: a population-based study. Int. J. Geriat. Psychiat..

[130] Fialová D., Topinková E., Gambassi G., Finne-Soveri H., Jónsson P.V., Carpenter I., Schroll M., Onder G., Sørbye L.W., Wagner C. (2005). Potentially inappropriate medication use among elderly home care patients in Europe. JAMA.

[131] Simon S.R., Chan K.A., Soumerai S.B., Wagner A.K., Andrade S.E., Feldstein A.C., Lafata J.E., Davis R.L., Gurwitz J.H. (2005). Potentially inappropriate medication use by elderly persons in U.S. Health Maintenance Organizations, 2000-2001. J. Am. Geriatr. Soc..

[132] Weinshilboum R. (2003). Inheritance and drug response. N. Engl. J. Med..

[133] Zhou S.F., Di Y.M., Chan E., Du Y.M., Chow V.D., Xue C.C., Lai X., Wang J.C., Li C.G., Tian M., Duan W. (2008). Clinical pharmacogenetics and potential application in personalized medicine. Curr. Drug Metab..

[134] Malhotra A.K., Lencz T., Correll C.U., Kane J.M. (2007). Genomics and the future of pharmacotherapy in psychiatry. Int. Rev. Psychiatry.

[135] Nnadi C.U., Malhorta A.K. (2007). Individualizing antipsychotic drug therapy in schizophrenia: the promise of pharmacogenetics. Curr. Psychiatry Rep..

[136] Foster A., Miller D., Buckley P.F. (2007). Pharmacogenetics and schizophrenia. Psychiatr. Clin. North Am..

[137] Arranz M.J., de Leon J. (2007). Pharmacogenetics and pharmacogenomics of schizophrenia: A review of last decade of research. Mol. Psychiatry.

[138] Mizutani T. (2003). PM frequencies of major CYPs in Asians and Caucasians. Drug Metab. Rev..

[139] Xie H.G., Prasad H.G., Kim R.B., Stein C.M. (2002). CYP2C9 allelic variants: ethnic ditribution and functional significance. Adv. Drug Deliv. Rev..

[140] Isaza C.A., Henao J., López A.M., Cacabelos R. (2000). Isolation, sequence and genotyping of the drug metabolizer CYP2D6 gene in the Colombian population. Meth. Find. Exp. Clin. Pharmacol..

[141] Ozawa S., Soyama A., Saeki M., Fukushima-Uesaka H., Itoda M., Koyano S., Sai K., Ohno Y., Saito Y., Sawada J. (2004). Ethnic differences in genetic polymorphisms of CYP2D6, CYP2C19, CYP3As and MDR1/ABCB1. Drug Metab. Pharmacokin..

[142] Xie H.G., Kim R.B., Wood A.J., Stein C.M. (2001). Molecular basis of ethnic differences in drug disposition and response. Annu. Rev. Pharm. Toxicol..

[143] Madan A., Graham R.A., Carroll K.M., Mudra D.R., Burton L.A., Krueger L.A., Downey A.D., Czerwinski M., Forster J., Ribadeneira M.D. (2003). Effects of prototypical microsomal enzyme inducers on cytochrome P450 expression in cultured human hepatocytes. Drug Metab. Dispos..

[144] Hedlund E., Gustafsson J.A., Warner M. (2001). Cytochrome P450 in the brain: a review. Curr. Drug Metabol..

[145] Funae Y., Kishimoto W., Cho T., Niwa T., Hiroi T. (2003). CYP2D in the brain. Drug Metab. Pharmacokin..

[146] Scordo M.G., Spina E. (2002). Cytochrome P450 polymorphisms and response to antipsychotic therapy. Pharmacogenomics.

[147] Ingelman-Sundberg M., Sim S.C., Gomez A., Rodríguez-Antona C. (2007). Influence of cytochrome P450 polymorphisms on drug therapies: pharmacogenetic, pharmacoepigenetic and clinical aspects. Pharmacol. Ther..

[148] Kobylecki C.J., Jakobsen K.D., Hansen T., Jakobsen I.V., Rasmussen H.B., Werge T. (2009). CYP2D6 genotype predicts antipsychotic side effects in schizophrenia inpatients: a retrospective matched case-control study. Neuropsychobiology.

[149] Kang R.H., Jung S.M., Kim K.A., Lee D.K., Cho H.K., Jung B.J., Kim Y.K., Kim S.H., Han C., Lee M.S., Park J.Y. (2009). Effects of CYP2D6 and CYP3A5 genotypes on the plasma concentrations of risperidone and 9-hydroxyrisperidone in Korean schizophrenic patients. J. Clin. Psychopharmacol..

[150] Yagihashi T., Mizuno M., Chino B., Sato Y., Sakuma K., Takebayashi T., Takao T., Kosaki K. (2009). Effects of the CYP2D6*10 alleles and co-medication with CYP2D6-dependent drugs on risperidone metabolism in patients with schizophrenia. Hum. Psychopharmacol..

[151] Fleeman N., McLeod C., Bagust A., Beale S., Boland A., Dundar Y., Jorgensen A., Payne K., Pirmohamed M., Pushpakom S. (2010). The clinical effectiveness and cost-effectiveness of testing for cytochrome P450 polymorphisms in patients with schizophrenia treated with antipsychotics: a systematic review and economic evaluation. Health Technol. Assess..

[152] Buchanan R.W., Freedman R., Javitt D.C., Abi-Dargham A., Lieberman J.A. (2007). Recent advances in the development of novel pharmacological agents for the treatment of cognitive impairments in schizophrenia. Schizophr. Bull..

[153] Tamminga C.A. (2006). The neurobiology of cognition in schizophrenia. J. Clin. Psychiatry.

[154] Malhotra A.K., Murphy G.M., Kennedy J.L. (2004). Pharmacogenetics of psychotropic drug response. Am. J. Psychiatry.

[155] de León J. (2009). The future (or lack of future) of personalized prescription in psychiatry. Pharmacol. Res..

[156] Need A.C., Keefe R.S., Ge D., Grossman I., Dickson S., McEvoy J.P., Goldstein D.B. (2009). Pharmacogenetics of antipsychotic response in the CATIE trial: a candidate gene analysis. Eur. J. Hum. Genet..

[157] Ikeda M., Tomita Y., Mouri A., Koga M., Okochi T., Yoshimura R., Yamanouchi Y., Kinoshita Y., Hashimoto R., Williams H.J. (2010). Identification of Novel Candidate Genes for Treatment Response to Risperidone and Susceptibility for Schizophrenia: Integrated Analysis Among Pharmacogenomics, Mouse Expression, and Genetic Case-Control Association Approaches. Biol. Psychiatry.

[158] Iwahashi K., Murayama O., Aoki J., Watanabe M., Ishigouoka J. (2009). Influence of serotonin (5-HT) 2A-receptor and transporter (5HTT) gene polymorphism upon the effect of olanzapine. Nihon Shinkei Seishin Yakurigaku Zasshi.

[159] Wei Z., Wang L., Xuan J., Che R., Du J., Qin S., Xing Y., Gu B., Yang L., Li H. (2009). Association analysis of serotonin receptor 7 gene (HTR7) and risperidone response in Chinese schizophrenia patients. Prog. Neuropsychopharmacol. Biol. Psychiatry.

[160] Chen S.F., Shen Y.C., Chen C.H. (2009). Effects of the DRD3 Ser9Gly polymorphism on aripiprazole efficacy in schizophrenic patients as modified by clinical factors. Prog. Neuropsychopharmacol. Biol. Psychiatry.

[161] Chen S.F., Shen Y.C., Chen C.H. (2009). HTR2A A-1438G/T102C polymorphisms predict negative symptoms performance upon aripiprazole treatment in schizophrenic patients. Psychopharmacology (Berlin).

[162] Park S.W., Lee J.G., Ha E.K., Choi S.M., Cho H.Y., Seo M.K., Kim Y.H. (2009). Differential effects of aripiprazole and haloperidol on BDNF-mediated signal changes in SH-SY5Y cells. Eur. Neuropsychopharmacol..

[163] Zuo L., Luo X., Krystal J.H., Cramer J., Charney D.S., Gelernter J. (2009). The efficacies of clozapine and haloperidol in refractory schizophrenia are related to DTNBP1 variation. Pharmacogenet. Genomics.

[164] Fatemi S.H., Reutiman T.J., Folsom T.D. (2009). Chronic psychotropic drug treatment causes differential expression of Reelin signaling system in frontal cortex of rats. Schizophr. Res..

[165] Guidotti A., Dong E., Kundakovic M., Satta R., Grayson D.R., Costa E. (2009). Characterization of the action of antipsychotic subtypes on valproate-induced chromatin remodeling. Trends Pharmacol. Sci..

[166] Cashman J.R., Zhang J., Nelson M.R., Braun A. (2008). Analysis of flavin-containing monooxygenase 3 genotype data in populations administered the anti-schizophrenia agent olanzapine. Drug Metab. Lett..

[167] Gupta M., Bhatnagar P., Grover S., Kaur H., Baghel R., Bhasin Y., Chauhan C., Verma B., Manduva V., Mukherjee O. (2009). Association studies of catechol-O-methyltransferase (COMT) gene with schizophrenia and response to antipsychotic treatment. Pharmacogenomics.

[168] Herken H., Erdal M., Aydin N., Sengul C., Karadag F., Barlas O., Akin F. (2009). The association of olanzapine-induced weight gain with peroxisome proliferator-activated receptor-gamma2 Pro12Ala polymorphism in patients with schizophrenia. DNA Cell Biol..

[169] Ujike H., Nomura A., Morita Y., Morio A., Okahisa Y., Kotaka T., Kodama M., Ishihara T., Kuroda S. (2008). Multiple genetic factors in olanzapine-induced weight gain in schizophrenia patients: a cohort study. J. Clin. Psychiatry.

[170] Basile V.S., Masellis M., McIntyre R.S., Meltzer H.Y., Lieberman J.A., Kennedy J.L.  (2001). Genetic dissection of atypical antipsychotic-induced weight gain: novel preliminary data on the pharmacogenetic puzzle. J. Clin. Psychiatry.

[171] Gunes A., Melkersson K.I., Scordo M.G., Dahl M.L. (2009). Association between HTR2C and HTR2A polymorphisms and metabolic abnormalities in patients treated with olanzapine or clozapine. J. Clin. Psychopharmacol..

[172] Gregoor J.G., van der Weide J., Mulder H., Cohen D., van Megen H.J., Egberts A.C., Heerdink E.R. (2009). Polymorphisms of the LEP- and LEPR gene and obesity in patients using antipsychotic medication. J. Clin. Psychopharmacol..

[173] Mulder H., Cohen D., Scheffer H., Gispen-de Wied C., Arends J., Wilmink F.W., Franke B., Egberts A.C. (2009). HTR2C gene polymorphisms and the metabolic syndrome in patients with schizophrenia: a replication study. J. Clin. Psychopharmacol..

[174] Kwon J.S., Joo Y.H., Nam H.J., Lim M., Cho E.Y., Jung M.H., Choi J.S., Kim B., Kang D.H., Oh S. (2009). Association of the glutamate transporter gene SLC1A1 with atypical antipsychotics-induced obsessive-compulsive symptoms. Arch. Gen. Psychiatry.

[175] Volpi S., Potkin S.G., Malhotra A.K., Licamele L., Lavedan C. (2009). Applicability of a genetic signature for enhanced iloperidone efficacy in the treatment of schizophrenia. J. Clin. Psychiatry.

[176] Choi K.H., Higgs B.W., Weis S., Song J., Llenos I.C., Dulay J.R., Yolken R.H., Webster M.J. (2009). Effects of typical and atypical antipsychotic drugs on gene expression profiles in the liver of schizophrenia subjects. BMC Psychiatry.

[177] Chana G., Lucero G., Salaria S., Lozach J., Du P., Woelk C., Everall I. (2009). Upregulation of NRG-1 and VAMP-1 in human brain aggregates exposed to clozapine. Schizophr. Res..

[178] Ji B., La Y., Gao L., Zhu H., Tian N., Zhang M., Yang Y., Zhao X., Tang R., Ma G. (2009). A comparative proteomics analysis of rat mitochondria from the cerebral cortex and hippocampus in response to antipsychotic medications. J. Proteome Res..

[179] Ma D., Chan M.K., Lockstone H.E., Pietsch S.R., Jones D.N., Cilia J., Hill M.D., Robbins M.J., Benzel I.M., Umrania Y. (2009). Antipsychotic treatment alters protein expression associated with presynaptic function and nervous system development in rat frontal cortex. J. Proteome Res..

[180] Dell’Aversano C., Tomasetti C., Iasevoli F., de Bartolomeis A. (2009). Antipsychotic and antidepressant co-treatment: effects on transcripts of inducible postsynaptic density genes possibly implicated in behavioural disorders. Brain Res. Bull..

[181] Cacabelos R. (2010). World Guide for Drug Use and Pharmacogenomics.

[182] Home Page of the Human Cytochrome P450 (CYP) Allele Nomenclature Committee. http://www.cypalleles.ki.se.

[183] PharmGKB. http://www.pharmgkb.org.

[184] Sachse C., Brockmoller J., Bauer S., Roots I. (1997). Cytochrome P450 2D6 variants in a Caucasian population: allele frequencies and phenotypic consequences. Am. J. Hum. Genet..

[185] Weinshilboum R.M., Wang L. (2006). Pharmacogenetics and pharmacogenomics: Development, science, and translation. Annu. Rev. Genomics Hum. Genet..

[186] Pilotto A., Franceschi M., D’Onofrio G., Bizzarro A., Mangialasche F., Cascavilla L., Paris F., Matera M.G., Pilotto A., Daniele A. (2009). Effect of a CYP2D6 polymorphism on the efficacy of donepezil in patients with Alzheimer disease. Neurology.

[187] Lamba J.K., Lin Y.S., Schuetz E.G., Thummel K.E. (2002). Genetic contribution to variable human CYP3A-mediated metabolism. Adv. Drug Deliv. Rev..

[188] Mannens G.S., Snel C.A., Hendrickx J., Verhaeghe T., Le Jeune L., Bode W., van Beijsterveldt L., Lavrijsen K., Leempoels J., van Osselaer N. (2002). The metabolism and excretion of galantamine in rats, dogs, and humans. Drug Metab. Dispos..

[189] Egger S.S., Bachmann A., Hubmann N., Schlienger R.G., Krähenbühl S. (2006). Prevalence of potentially inappropriate medication use in elderly patients. Comparison between general medicine and geriatric wards. Drugs Aging.

[190] Roses A.D. (2009). The medical and economic roles of pipeline pharmacogenetics: Alzheimer’s disease as a model of efficacy and HLA-B(*)5701 as a model of safety. Neuropsychopharmacology.

[191] Roses A.D., Saunders A.M., Huang Y., Strum J., Weisgraber K.H., Mahley R.W. (2007). Complex disease-associated pharmacogenetics: drug efficacy, drug safety, and confirmation of a pathogenic hypothesis (Alzheimer’s disease). Pharmacogenomics J..

[192] Risner M.E., Saunders A.M., Altman J.F., Ormandy G.C., Craft S., Foley I.M., Zvartau-Hind M.E., Hosford D.A., Roses A.D., Rosiglitazone in Alzheimer’s Disease Study Group (2006). Efficacy of rosiglitazone in a genetically defined population with mild-to-moderate Alzheimer’s disease. Pharmacogenomics J..

[193] Lombardi V.R.M., Cacabelos R. (1999). E-SAR-94010: a marine fish extract obtained by advanced biotechnological methods. Drugs Future.

[194] Cacabelos R., Vallejo A.I., Lombardi V., Fernández-Novoa L., Pichel V. (2004). E-SAR-94010 (LipoEsar®): A pleiotropic lipoprotein compound with powerful anti-atheromatous and lipid lowering effects. CNS Drug Rev..

[195] Cacabelos R. (2004). Pharmacogenomics and Nutraceuticals. Scientific Progress and Pharmaceutical Development.

[196] Philips K.A., van Bebber S.L. (2005). Measuring the value of pharmacogenomics. Nat. Rev. Drug Discovery.

[197] Veenstra D.L., Higashi M.K. (2000). Assessing the cost-effectiveness of pharmacogenomics. AAPS PharmSci..

[198] Sink K.M., Holden K.F., Yaffe K. (2005). Pharmacological treatment of neuropsychiatric symptoms of dementia. A review of the evidence. JAMA.

[199] van Steen K., McQueen M.B., Herbert A., Raby B., Lyon H., Demeo D.L., Murphy A., Su J., Datta S., Rosenow C. (2005). Genomic screening and replication using the same data set in family-based association testing. Nat. Genet..

[200] Eichelbaum M., Ingelman-Sundberg M., Evans W.E. (2006). Pharmacogenomics and individualized drug therapy. Annu. Rev. Med..

[201] Sowell R.A., Owen J.B., Butterfield D.A. (2009). Proteomics in animal models of Alzheimer’s and Parkinson’s disease. Ageing Res. Rev..

[202] Khachaturian Z.S., Petersen R.C., Gauthier S., Buckholtz N., Corey-Bloom J.P., Evans B., Fillit H., Foster N., Greenberg B., Grundman M. (2008). A roadmap for the prevention of dementia: The inaugural Leon Thal Symposium. Alzheimers Dement..

[203] Cacabelos R. (2005). Role of nutrition in the prevention of Alzheimer’s disease. Aging Health.

[204] Need A.C., Motulsky A.G., Goldstein D.B. (2005). Priorities and standards in pharmacogenetic research. Nat. Genet..

[205] Johnson A.D., Wang S., Sadee W. (2005). Polymorphisms affecting gene regulation and mRNA processing: broad implications for pharmacogenetics. Pharmacol. Ther..

[206] Ishikawa T., Onishi Y., Hirano H., Oosumi K., Nagakura M., Tarui S. (2004). Pharmacogenomics of drug transporters: a new approach to functional analysis of the genetic polymorphisms of ABCB1 (P-glycoprotein/MDR1). Biol. Pharm. Bull..

[207] Nishimura M., Naito S. (2008). Tissue-specific mRNA expression profiles of human solute carrier transporter superfamilies. Drug Metab. Pharmacokin..

[208] Wilcock D.M., Colton C.A. (2008). Anti-Aß immunotherapy in Alzheimer’s disease: relevance of transgenic mouse studies to clinical trials. J. Alzheimer Dis..

[209] Agrawal N., Dasaradhi P.V., Mohammed A., Malhotra P., Bhatnagar R.K., Mukherjee S.K. (2003). RNA interference: biology, mechanism, and applications. Microbiol. Mol. Biol. Rev..

[210] Spänkuch B., Strebhardt K. (2005). RNA interference-based gene silencing in mice: the development of a novel therapeutical strategy. Curr. Pharm. Des..

[211] Leung R.K., Whittaker P.A. (2005). RNA interference: from gene silencing to gene-specific therapeutics. Pharmacol. Ther..

[212] Ying S.Y., Lin S.L. (2009). Intron-mediated RNA interference and microRNA biogenesis. Methods Mol. Biol..

[213] González-Alegre P. (2007). Therapeutic RNA interference for neurodegenerative diseases: From promise to progress. Pharmacol. Ther..

[214] Aagaard L., Rossi J.J. (2007). RNA therapeutics: principles, prospects and challenges. Adv. Drug Deliv. Rev..

[215] Hébert S.S., Horré K., Nicolaï L., Bergmans B., Papadopoulou A.S., Delacourte A., de Strooper B. (2009). MicroRNA regulation of Alzheimer’s amyloid precursor protein expression. Neurobiol. Dis..

[216] Feng Z., Zhao G., Lei Y. (2009). Neural stem cells and Alzheimer’s disease: challenges and hope. Am. J. Alzheimers Dis. Other Demen..

